# Hexabenzocoronene–Benzimidazole Hybrid Architectures and Faraday Rotation of the First Hexabenzocoronene–Phthalocyanine

**DOI:** 10.1002/anie.202522494

**Published:** 2025-12-16

**Authors:** Antonia Rocha‐Ortiz, Abdusalom A. Suleymanov, Pascal Puhlmann, Dustin Krischer, Carolin Müller, Dirk Zahn, Timothy M. Swager, Andreas Hirsch

**Affiliations:** ^1^ Department of Chemistry & Pharmacy Chair of Organic Chemistry II Friedrich‐Alexander‐Universität Erlangen‐Nürnberg Nikolaus‐Fiebiger‐Straße 10 91058 Erlangen Germany; ^2^ Department of Chemistry Massachusetts Institute of Technology Cambridge Massachusetts 02139 USA; ^3^ Interdisciplinary Center for Molecular Materials (ICMM) and Computer Chemistry Center (CCC) Friedrich‐Alexander‐Universität Erlangen‐Nürnberg Nägelsbachstraße 25 91052 Erlangen Germany; ^4^ Computer Chemistry Center (CCC) Friedrich‐Alexander‐Universität Erlangen‐Nürnberg Nägelsbachstraße 25 91052 Erlangen Germany

**Keywords:** Absorption spectroscopy, Electronic structure, Faraday rotation, Hexabenzocoronene, Phthalocyanine

## Abstract

The synthesis and characterization of a first example of a Ni‐phthalocyanine (Pc) hybrid molecule carrying four hexa‐*peri‐*hexabenzocoronene (HBC) substituents and of three HBC‐benzimidazole derivatives is reported. These π‐electron rich chromophores exhibit a high molar absorption coefficient with extended absorption up to 460 nm. Particularly, HBC‐Pc absorbs throughout most of the UV–vis range with the Pc Q‐band located at 672 nm. This enables its investigation as a Faraday rotator where it displayed a remarkable Verdet constant of −1.4 × 10^5^ deg T^−1^m^−1^ at 700 nm assigned to Faraday A‐term activity. Electrochemical measurements supported by DFT calculations elucidated the electronic structure of the molecules together with excited state molecular orbital analysis using TDDFT. Here, HBC‐Pc showed a HOMO/LUMO gap considerably lower than the other HBC‐based compounds due to the introduction of the Pc scaffold.

## Graphical abstract

The synthesis of four hexabenzocoronene (HBC) benzimidazole hybrid molecules is shown, culminating in tetra‐HBC‐Ni‐phthalocyanine (Pc). Its application as a Faraday rotator reveals a remarkable Verdet constant. The compounds are fully characterized and their optoelectronic properties investigated by electrochemistry, UV–vis, and fluorescence spectroscopy. Density functional theory (DFT) calculations support the experimental data.

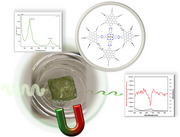

## Introduction

**Scheme 1:**
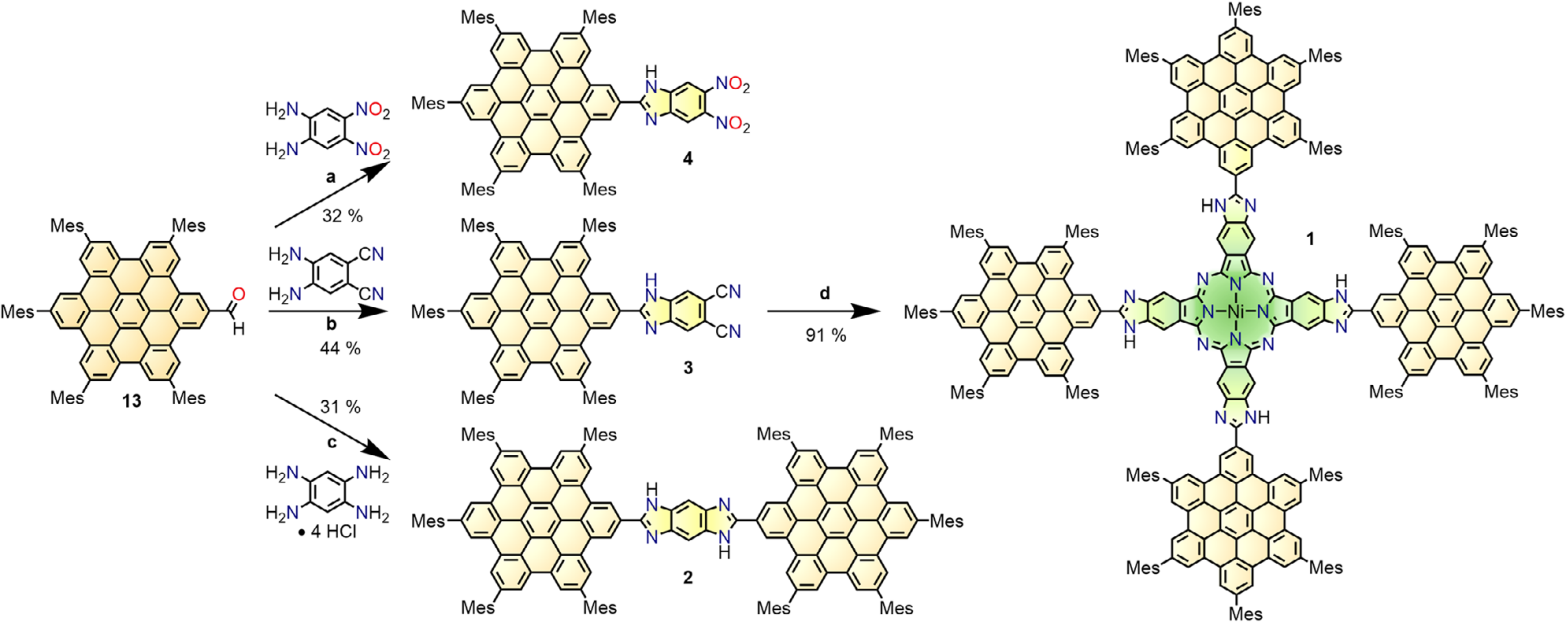
Synthetic approach toward target HBC hybrids **1**‐**4**: **a**) Zn(OTf)_2_, THF, 120°C, 7 h; **b**) Zn(OTf)_2_, DMF, 170°C, 8 h; **c**) 1. TEA, DMF, 30 min, rt; 2. Zn(OTf)_2_, DMF, 170° C, 15 h; **d**) NiCl_2_, DBU, 130°C, 14 d.

When considering new functional materials for organic electronics, nanographenes are very promising building blocks.^[^ ^1^, ^2^ ^]^ One of the most prominent examples is hexa‐*peri*‐hexabenzocoronene (HBC), valued for its facile, geometrically accurate synthesis, versatile functionalization potential, its optoelectronic properties, and its ability to stack into organized columnar structures and even liquid crystals.^[^ ^3^, ^4^, ^5^ ^]^ The selective synthesis of HBCs with various different functional groups and their arrangement in the HBCs periphery has been successfully realized and optimized.^[^ ^6^, ^7^ ^]^ Additionally, also monofunctionalized HBCs have revealed outstanding properties in applications such as organic electronics and photovoltaics.^[^ ^8^, ^9^ ^]^ In photovoltaic applications, HBCs have taken the role of electron donors both in donor‐acceptor (D‐A) blends as well as in covalently linked D‐A hybrid molecules.^[^ ^10^, ^11^, ^12^, ^13^, ^14^, ^15^ ^]^ Apart from their direct activity in applications, HBC‐based D‐A hybrid systems have been investigated with different types of linkers between the units in elaborated nanoscopic systems: covalently bonded systems, assemblies connected by acetylene spacers, linearly π‐extended, or fused aromatic systems have been synthesized and exhibit useful properties such as charge transfer and directed self‐assembly.^[^ ^7^, ^16^, ^17^, ^18^ ^]^ In the instance of hybrid dye systems with porphyrins, HBC can not only be employed as the central unit between covalently bonded porphyrins but has also been shown surrounding a central benzoporphyrin utilizing several binding motifs.^[^ ^19^, ^20^ ^]^ Among reported D‐A architectures, HBCs can be connected to an electron‐poor moiety leading to a D–A dyad or on either side of a bridging unit to yield linear motifs. All three aforementioned molecular designs are presented in this paper: two HBC hybrid molecules substituted with electron‐poor benzimidazole moieties (HBCs **3** and **4**) and a linearly benzodiimidazole‐bridged HBC dimer **2** (Scheme 1). Thirdly, HBC‐phthalonitrile **3** can be converted into tetra‐HBC Ni‐phthalocyanine HBC‐Pc **1** *via* cyclotetramerization (Scheme 1). Thus, the former D‐A motif is removed and a new set of properties and functions based on the central phthalocyanine (Pc) unit can be combined with those of the connected HBCs. Phthalocyanines continue to reveal new versatility in their tunability and applications even after decades of research.^[^ ^21^, ^22^ ^]^ Their utility ranges from usage as pigments,^[^ ^23^ ^]^ to use as conducting materials^[^ ^24^, ^25^ ^]^ in modern appliances like organic photovoltaics^[^ ^26^, ^27^ ^]^ and organic thin‐film transistors,^[^ ^28^ ^]^ as well as very recently, as elements of metal–organic frameworks and covalent organic frameworks.^[^ ^29^, ^30^, ^31^, ^32^ ^]^ Another emerging application of phthalocyanines stems from their large Faraday rotations – the magneto‐optical phenomenon in which the plane of linearly polarized light rotates as it passes through a material subjected to a magnetic field parallel to the direction of light propagation as described by Equation (1).^[^ ^33^ ^]^ The Faraday rotation angle *φ* (in degrees) is determined in a proportional fashion by the Verdet constant *V* (in deg T^−1^m^−1^), an intrinsic property of every material, the magnetic field strength *B* (in T) and the optical path length *d* (in m), according to

(1) φ=VBd

A typical Verdet constant of the conventional Faraday rotator terbium gallium garnet (TGG), an inorganic crystal, lies in the range of 10^4^ deg T^−1^m^−1^ at 532 nm.^[^ ^34^ ^]^ Recently, organic materials such as phthalocyanines have emerged as promising alternatives due to their highly symmetric π‐conjugated frameworks, thermal stability, tunable electronic properties as well as their ability to form light and flexible thin films.^[^ ^33^ ^]^ In symmetric phthalocyanines, Faraday rotation arises from magnetic‐field‐induced circular birefringence, linked to Zeeman splitting of excited states (Faraday A‐term), which are associated with the optical transitions linked to the Q‐band absorption.^[^ ^35^, ^36^, ^37^, ^38^ ^]^ By fusing four thianthrene units to a phthalocyanine (ZnPc‐OT), an exceptionally high Verdet constant of −3.3 × 10⁵ deg T^−1^m^−1^ at 800 nm has been reported by some of us, which is attributed to enhanced polarizability from sulfur atoms.^[^ ^35^ ^]^ We have wondered if similarly high rotations can be achieved by increased polarizability through the attachment of large aromatic moieties and an associated extended π‐system. To test this strategy, we investigated the new phthalocyanine derivative HBC‐Pc **1** bearing four hexabenzocoronene (HBC) units. Synthetically, symmetric phthalocyanines are easily accessible *via* cyclotetramerization of a phthalonitrile. Depending on the functionalization of the phthalonitrile, an abundance of substituents can be introduced into the periphery of the phthalocyanine. Particularly imidazo‐moieties fused to the phthalocyanine´s periphery show interesting properties such as bathochromically shifted Q‐bands and pH‐sensing abilities; however, their representation in literature remains limited.^[^ ^39^, ^40^, ^41^, ^42^, ^43^, ^44^ ^]^ An additional advantage of fusing imidazole derivatives to phthalocyanines is the possibility to introduce further functional scaffolds *via* the 2‐position of the imidazo‐moiety. Herein, we apply this approach to connect HBC to Pc by first forming HBC‐benzimidazophthalonitrile **3**, which is consecutively cyclotetramerized to HBC‐Pc **1**. To the best of our knowledge, this is the first time a closed HBC derivative is covalently connected to a phthalocyanine.^[^ ^45^, ^46^ ^]^ Successively, this highly symmetrical nanographene‐phthalocyanine hybrid compound, which additionally benefits from good solubility in common organic solvents, is investigated as a Faraday rotator, where it is shown to display a large Verdet constant.

## Results and Discussion

### Synthesis

The target HBC‐benzimidazole derivatives **2**, **3**, and **4** are synthesized *via* condensation reactions from HBC‐ aldehyde **13** with the respective diamines as depicted in Scheme 1. Subsequently, HBC‐imidazophthalo‐nitrile **3** itself serves as a precursor to form tetra‐HBC Ni‐phthalocyanine **1** *via* cyclotetramerization (Scheme 1).

A schematic display of the precursor synthesis is shown in Scheme S1. The key precursor HBC‐aldehyde **13** was synthesized by the generation of monosubstituted HBCs *via* Diels–Alder cycloaddition of a symmetric cyclopentadienone (molecule **8**) with an asymmetric diphenyl acetylene (molecule **11**) carrying the functional group.^[^ ^47^, ^48^, ^49^ ^]^ Mesityl moieties were chosen since they proved superior relative to the literature known *tert‐*butyl analogs for increasing the solubility of HBCs.^[^ ^50^ ^]^ The components of cyclopentadienone **8** were synthesized first by attaching mesityl boronic acid to 4,4′‐dibromobenzil and to 1,3‐bis(4‐bromophenyl)‐acetone **6** in double Suzuki cross‐coupling reactions, which were then combined in a Knoevenagel condensation to yield molecule **8**. Secondly, diphenyl acetylene **11** was achieved in a Sonogashira cross‐coupling of iodobenzaldehyde with literature‐known (4‐mesitylphenyl)acetylene **10**. Next, the aforementioned Diels‐–Alder cycloaddition between molecules **8** and **11** was carried out, leading to HPB‐aldehyde **12**, the direct precursor of HBC‐aldehyde **13**, which, in turn, was achieved in a Scholl reaction employing DDQ and triflic acid.^[^ ^7^, ^49^ ^]^ The characterization of these precursors by means of proton and carbon NMR spectroscopy as well as high resolution mass spectrometry (HRMS) is reported in Figures S5–S17 and S22–S34.

With HBC‐aldehyde **13** at hand, various condensation reactions could be carried out such as the coupling of the HBC‐building block to differently substituted *ortho*‐diamino benzenes under benzimidazole formation (Scheme 1). First, HBC‐dinitrobenzimidazole **4** was designed as a straightforward, linear donor‐acceptor system, its electron‐withdrawing effect being introduced by the nitro groups. Since the solvent used in literature for similar condensation reactions (ethanol) provided no solubility for our HBC derivatives, THF was chosen instead.^[^ ^51^ ^]^ However, despite extended reaction duration, a reflux temperature of ≈ 70°C afforded unsatisfactory amounts of product. Under microwave irradiation and a reaction vessel that allowed for elevated temperatures up to 120°C, HBC‐benzimidazole **4** could be obtained in 32% yield and sufficient amounts for characterization and investigation of its optoelectronic properties. HBC‐imidazophthalonitrile **3** was required in somewhat larger quantities because it is intended to not only be studied for its own properties but also to be used to produce another material. Therefore, the solvent system was again adjusted to DMF to increase the reaction temperature under microwave irradiation to 170°C. After overnight reaction and purification, the compound was obtained in 44% yield, providing a pleasing improvement compared to HBC **4**. In the proton NMR spectrum, all expected signals are resolved and assigned to their respective protons (Figure S3‐1). In comparison to HBC **4**, the ^1^H NMR spectrum is similar for HBC **3** with different chemical shifts (Figure S4‐1), and the carbon spectrum displays an additional peak at 117.0 ppm that is assigned to the nitrile substituents (**3**: Figure S3‐2; HMBC: S3‐6; **4**: Figure S4‐2). Additionally, formation of both compounds was confirmed by HRMS (Figures S20, S21).

Penultimately, in di‐HBC‐benzodiimidazole **2**, an electron donating HBC building block is placed on either side of the connecting benzodiimidazole bridge. For the synthesis, first, 1,2,4,5‐tetraaminobenzene had to be released from its tetrahydrochloride salt in situ, only after which HBC‐aldehyde **13** was added. DMF was again employed as solvent and the reaction was microwave‐heated for 15 h to ensure double condensation of the HBC moieties to the bridging unit, thus forming a central benzodiimidazole. Due to the linear connection of two HBC moieties on a benzodiimidazole, the solubility was reduced compared to the monomeric HBCs **3** and **4**. This feature resulted in losses during several column chromatographies and the product was isolated in 31% yield. In the proton NMR spectrum, all expected signals of the molecule's protons were detected (Figures S2‐1 to S2‐3), also the signals caused by the protons of the bridging unit of the *cis*‐isomer can be observed. Additionally, HBC **2** was characterized by carbon NMR spectroscopy (Figure S2‐4) and also confirmed by MS analysis (Figure S19).

Finally, HBC‐imidazophthalonitrile **3** was subjected to cyclotetramerization to create tetra‐HBC Ni‐phthalocyanine **1**, in which four HBC groups surround the central phthalocyanine unit with imidazo moieties fused to the phthalocyanine in β‐position. Nickel as a central atom was chosen due to its stability in phthalocyanines and porphyrins and diamagnetism facilitating NMR spectroscopy. The reaction was performed in dry 1‐pentanol with NiCl_2_ and DBU at 130 °C. During the reaction, which lasted 14 days, a continuously intensifying color change occurred from the greyish–yellow starting reaction mixture and at the point of ending the reaction the color was dark green. Monitoring *via* TLC (SiO_2_, DCM + 1% triethylamine (TEA)) revealed only negligible remains of starting material HBC **3** after two weeks when the reaction was stopped. Apart from silica plug filtration in the above‐mentioned solvent mixture to remove the catalyst, the product was purified by gel permeation chromatography (BioBeads SX1, CHCl_3_) and obtained as a dark green, almost black solid with a metallic green luster in 91% yield. The MALDI MS matched the peak profile calculated for HBC‐Pc **1** (Figure S18), and the molecule was further characterized by NMR spectroscopy (Figure S1). The latter measurement proved to be challenging despite the surprisingly good solubility of HBC‐Pc **1** since the 280 protons are shared with the precursor HBC **3**. All signals of the previously identified protons appear as expected, with the eight HBC‐protons “f” giving rise to the singlet at 9.59 ppm, while the signals associated with the eight phthalocyanine protons likely overlap with the remaining ones of the HBC units at 8.83 ppm and the respective shoulder at 8.77 ppm, as is characteristic for the phthalocyanine system.^[^ ^52^ ^]^ The proton signals were resolved in tetrachloroethane‐d_2_ at elevated temperatures; however, these conditions were unsuitable for generating a resolved carbon spectrum. Instead, the DEPTq135 NMR spectrum depicted in Figure S1‐2 was recorded in DCM‐d_2_ at room temperature. The characteristic Pc signals around 130–140 ppm suffer from signal broadening and too much overlap with the HBC‐related carbon signals to be distinguishable.^[^ ^53^ ^]^

The characterized four HBC‐benzimidazole hybrid molecules were then investigated for their optoelectronic properties by means of steady‐state absorption and emission spectroscopy complemented by electrochemical measurements. Furthermore, HBC‐Pc **1** was tested for its properties as a Faraday rotator. Additionally, density functional theory calculations (DFT and TDDFT) were employed for geometry optimization and molecular orbital analysis. The optimized geometry of HBC‐Pc **1** in vacuum.

### Steady‐State Absorption and Emission Properties

**Figure 1:**
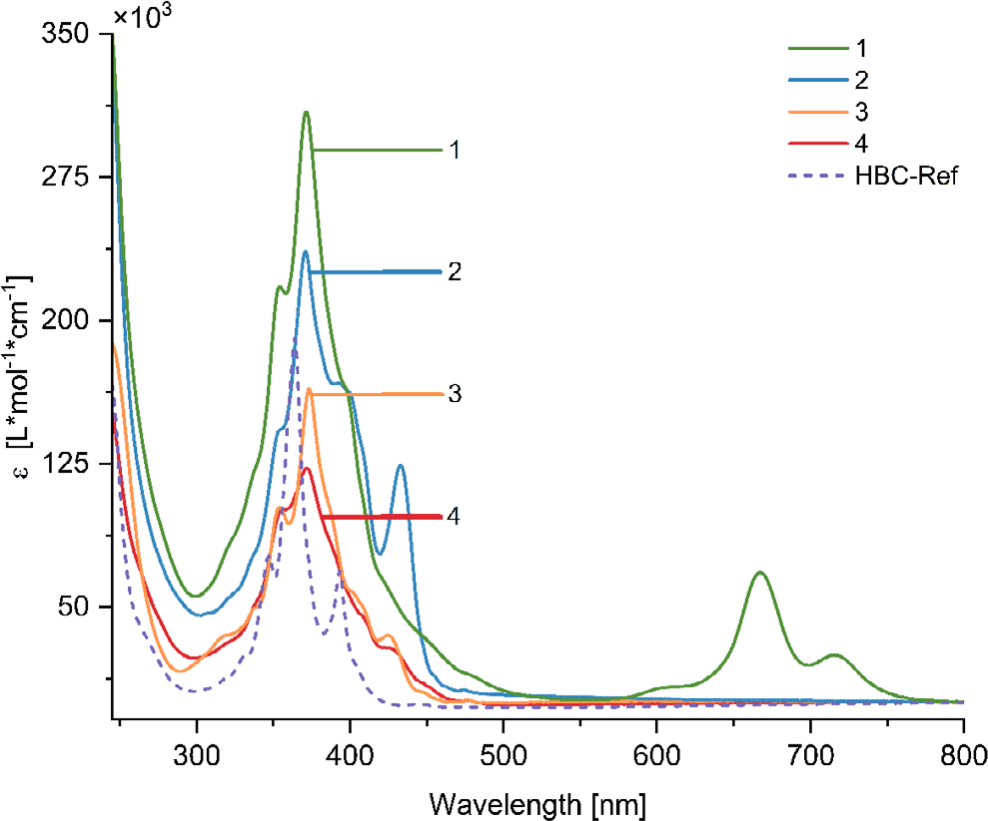
Steady‐state absorption spectra of compounds **1‐4** and **HBC‐Ref** in DCM at room temperature.

All steady‐state absorption and emission measurements of the target compounds **1**, **2**, and **4**, as well as target‐precursor **3** and the hexamesityl‐HBC reference (**HBC‐Ref**) were performed in DCM to ensure complete and equal solubility of the molecules, as well as comparability between measurements (Figure 1). As HBC derivatives, all four compounds exhibit the trident‐shaped β′‐, β‐, and p‐bands typical for HBCs and weak α‐band absorptions above 450 nm. The β′‐ and β‐bands appear in all four compounds around 355 nm and above 370 nm, respectively, with the global absorption maximum corresponding to the β‐band. These, particularly, are bathochromically shifted by 7–9 nm (0.06–0.08 eV) with respect to **HBC‐Ref** (364 nm) and by over 10 nm compared to the literature known wavelength of 360 nm for HBC β‐bands.^[^ ^54^ ^]^ At these wavelengths, the molar absorption coefficients ε of HBCs **3** and **4,** which consist of only one HBC moiety each lie at 1.60 × 10^5^ L mol^−1^ cm^−1^ and 1.20 × 10^5^ L mol^−1^ cm^−1^, slightly lower than the typical range for single‐HBC derivatives, with HBC **3** being the more absorbent compound.^[^ ^47^ ^]^ This becomes apparent as well when compared to **HBC‐Ref**, whose molar absorption coefficient at 364 nm is 1.95 × 10^5^ L mol^−1 ^cm^−1^, a higher value than is observed for HBCs **3** and **4**. Since both HBC **2** and HBC‐Pc **1** comprise two and four HBC moieties, respectively, they also exhibit larger ε‐values. However, this trend does not increase linearly with addition of HBC subunits to the scaffold, but values of 2.30 × 10^5^ L mol^−1 ^cm^−1^ (**2**) and 3.20 × 10^5^ L mol^−1 ^cm^−1^ (**1**) are observed instead (Table 1). This decrease in absorption upon inclusion of further HBC moieties compared to single HBCs is likely due to intermolecular stacking, which has been described as well by Lungerich *et al*.^[^ ^47^ ^]^

**Table 1:** Steady‐state absorption and fluorescence spectroscopy of molecules **1‐4** and **HBC‐Ref** measured in DCM solution.

Molecule:	**1**	**2**	**3**	**4**	**HBC‐Ref**
*λ*_max_Abs. (ε)^a)^	371 nm (3.24) 672 nm (0.55)	371 nm (2.32) 433 nm (1.21)	373 nm (1.58)	372 nm (1.22)	364 nm (1.96)
*λ*_max_ Fl.^b)^	481 nm^c)^	476 nm^c)^	479 nm^c)^	481 nm^c)^	469 nm^d)^ 489 nm^e)^

^a)^ Molar absorption coefficient ε × 10^5^ [L mol^−1^ cm^−1^];

^b)^ upon excitation into the respective absorption maximum;

^c)^ both 1st and maximum intensity emission band;

^d)^ 1^st^ emission band;

^e)^ maximum intensity emission band.

Going to longer wavelengths, the p‐band is located in the expected region between 390 and 395 nm, although it becomes apparent rather as a shoulder than as a local maximum in all four spectra.^[^ ^54^ ^]^ In general, the trident‐shape of β′‐, β‐, and p‐bands is less defined for HBCs **1‐4** than for **HBC‐Ref**, which can be attributed to the overlap with the benzimidazole‐/benzodiimidazole absorption in the same region of around 360 nm.^[^ ^55^, ^56^ ^]^ In case of HBCs **2‐4**, additional absorption bands are observed at 433 nm (**2**) and 425 nm (**3**, **4**) with the former one being particularly intense. For all four target molecules, weak α‐band absorptions appear around 450 nm as the related transitions are symmetry‐forbidden.^[^ ^54^ ^]^ These aspects, including the range of ε‐values, can be related to the presence of benzimidazole units in **3** and **4** (benzodiimidazole bridge in **2**), which can introduce additional charge‐transfer absorption features. These features alter the shape and fine structure of the absorption band, affecting both the position and relative intensity of local absorption maxima.

It has to be noted, however, that HBC‐Pc **1** exhibits enhanced absorption throughout the entire region between 300–500 nm with comparatively unresolved bands. This can be associated with aggregation as well as an overlap with the phthalocyanine Soret‐band (B‐band), which characteristically appears between 300–400 nm.^[^ ^43^, ^52^, ^53^ ^]^

Although HBCs **2‐4** do not further absorb above 500 nm, the phthalocyanine's Q‐band absorption appears with a maximum at 672 nm with a shoulder located around 605 nm, and a further local maximum can be observed at 715 nm. Compared to unsubstituted Ni‐Pc (*λ*_max_ = 670 nm in chloronaphthalene), the latter absorption maximum is bathochromically shifted by 45 nm (0.12 eV), a typical observation for imidazo‐substituted phthalocyanines.^[^ ^40^, ^42^ ^]^ The occurrence of two Q‐bands is likely related to the presence of aggregated‐ and non‐aggregated species in solution, respectively, manifesting in the absorption bands at 672 nm and 715 nm (see also TDDFT calculations).^[^ ^43^, ^44^ ^]^ Additional steady‐state absorption measurements in 1,2‐dichlorobenzene as a more disaggregating solvent show a concentration dependence of the lower energy band, which decreases with respect to the higher energy Q‐band in lower concentrations, thus underlining the likelihood of a relation to aggregated species (Figure S39‐3). As anticipated, no symmetry‐induced splitting of the Q‐band is observed for the symmetric HBC‐Pc **1**.^[^ ^43^, ^52^ ^]^

**Figure 2:**
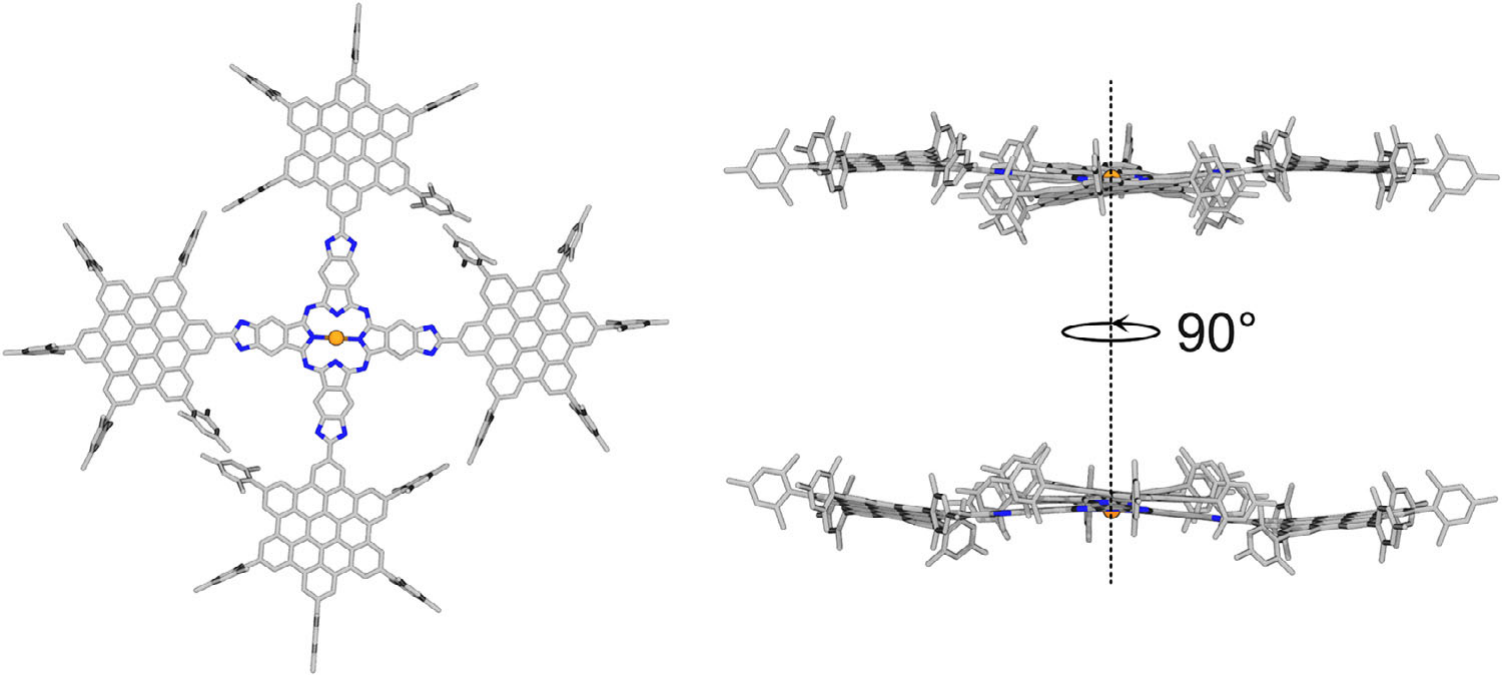
Geometry optimized structure of HBC‐Pc **1** (only nickel, carbon, and nitrogen atoms are shown, while hydrogen atoms are omitted). Left: top view, right: side view. The structure shows high symmetry in a C2 axis through the nickel center. The opposing HBC paddles are bent downwards and upwards respectively. The mesityl groups are in general perpendicular to the rest of the molecule but show some twisting when being in contact to another mesityl group of a neighboring HBC paddle. The bottom of the side views is 90° rotated to the top side view.

**Figure 3:**
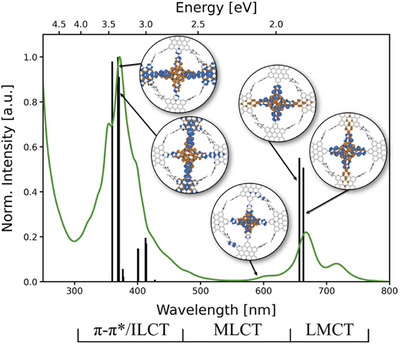
Comparison between the experimental absorption spectrum for HBC‐Pc **1** (green line) and the calculated spectrum (black bars) with charge density differences plots for selected transitions (excitation goes from blue/dark (negative) to orange/light (positive); only nickel, carbon, and nitrogen atoms are shown). The calculation shows reasonably good matching with the measured absorption spectrum (redshifted by 0.25 eV). In the low energy region, we find ligand‐to‐metal charge‐transfers (LMCT) at high intensity. Transitions were also found between 460 and 630 nm but with very low oscillator strength (*f* < 0.0002). Those weak signals show metal‐to‐ligand charge‐transfers (MLCTs). The signals with highest intensity are π–π* and inter‐ligand charge transfers (ILCT). For illustration, a series of charge difference plots are shown for selected excitations (Figure S46).

For a better understanding of the geometry and electronic structure of the HBC derivates, DFT calculations were performed. Geometry optimizations were accomplished at the BP86‐D4/DEF2‐SVP level. The optimized geometry of HBC‐Pc **1** in vacuum is shown in Figure 2, while the relation between absorption bands and respective transitions is depicted in Figure 3, where it is also compared to the experimental results and discussed in detail.

To compute the electronic absorption spectra of HBC's **1–4**, TDDFT calculations at the CAM‐B3LYP‐D4/DEF2‐SVP level was performed using the 30 lowest singlet states in the Franck–Condon region. The resulting absorption spectru of HBC‐Pc **1** shows a reasonably good matching with the experiment (see calculated transitions vs. green line in Figure 3), with the calculated vacuum spectrum being redshifted by 0.25 eV. The highest intensities are also in the high energy region. Excitation to S_1_ and S_2_ states are found in the low energy region (663 nm and 657 nm after shifting) and are mainly HOMO → LUMO and HOMO → LUMO + 1 transitions.

With the help of charge density difference plots, we found that excitation from the HOMO to the states S_1_ and S_2_ are ligand‐to‐metal charge‐transfers (LMCT). In turn, excitation to the states S_3‐7_ have a low oscillator frequency (*f* < 0.0002; as compared to *f* = 1.3779 and f = 1.4953 for S_1_ and S_2_, respectively) and show metal‐to‐ligand charge‐transfers (MLCT) between 460 and 630 nm, i.e., can be associated with the comparably very weak absorption band observed between 500 and 600 nm.

In the measured spectrum, we find low absorption between 450 and 600 nm, which is in line with the low intensities predicted from TDDFT in this region. In the TDDFT calculations, we were unable to find transitions at higher wavelengths than experimental peak at 672 nm. We thus argue that the second peak in the experiment at 715 nm stems from aggregate formation or reflects a vibrational shift (the difference of the corresponding wavenumbers is only 895 cm^−1^); however, given the experimental results measured in 1,2‐dichlorobenzene, aggregate formation seems likely. Both charge density difference plots and calculated absorption spectra for compounds **2**‐**4** are reported in Figures S47–S54.

**Figure 4:**
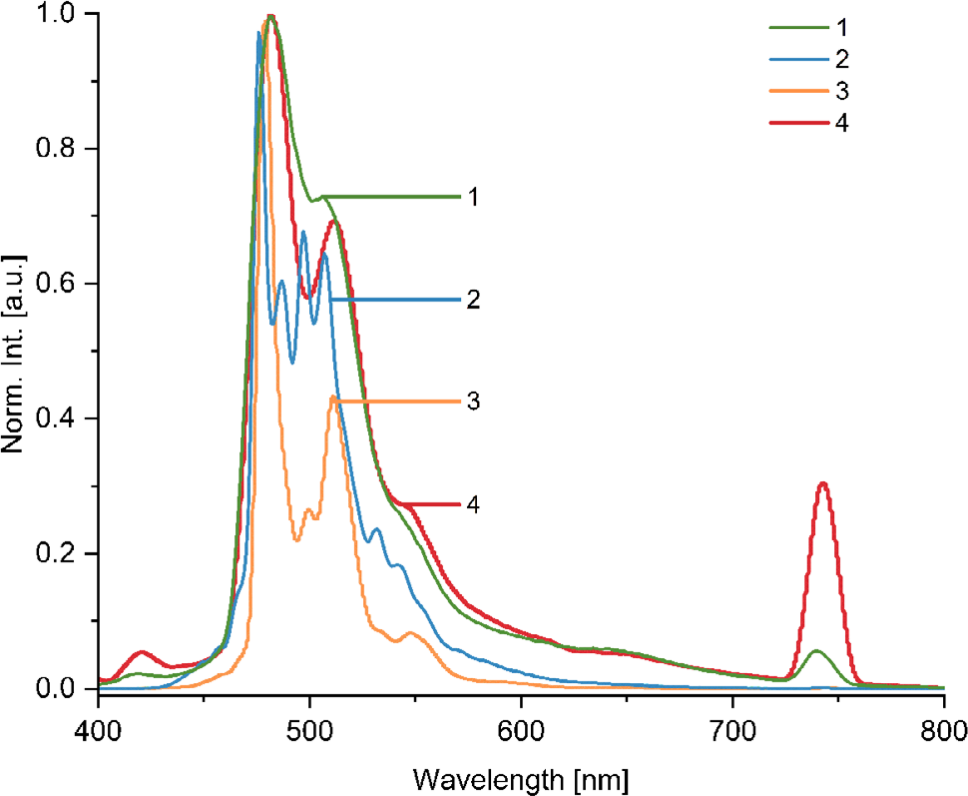
Normalized steady‐state emission spectra of compounds **1‐4** in DCM at room temperature.

Furthermore, steady‐state fluorescence of all four compounds as well as **HBC‐Ref** was measured in DCM; the spectra were obtained upon irradiation into the absorption maximum. First, different emission intensities were apparent that required various settings to obtain the spectra and the results depicted are in normalized form (Figure 4). Although HBCs **2** and **3** exhibit relatively intense fluorescence that is visible by bare eye as well, the emission from HBC **4** and HBC‐Pc **1** could only be detected utilizing enhanced device sensitivity achieved by a broadened excitation‐ and emission slit width of the spectrometer. Note the compared intensity of 2nd order diffraction is strongly enhanced in compounds **1** and **4** with regard to the maximum emission band, while not appearing in the spectra of HBC **2** and **3**. In case of HBC‐Pc **1**, the measured fluorescence stems entirely from the HBC moieties, while the phthalocyanine´s emission is expectedly quenched by the Ni‐center. This is demonstrated as well in the excitation spectrum, which mimics only the HBC region of the spectrum (300–500 nm), whereas no excitation can be observed in the Q‐band region between 600 and 800 nm. The excitation spectra of HBCs **2‐4** correspond directly to their absorption spectra (Figure S44).

The global emission maxima appear always on the high energy side for all four molecules, unlike **HBC‐Ref** with a first emission maximum located at 469 nm, the global maximum lies at 489 nm, and a fine‐structure including six sharp emission bands is resolved (Figure S45). When considering the four target molecules, the highest energy emission appears in HBC **2** at 476 nm, which is still bathochromically shifted by 10 nm compared to **HBC‐Ref**. Further three emission bands of lower intensity can be observed at 487, 497, and 507 nm. Consecutively, the emission of HBC **3** reached its maximum at 489 nm with additional bands arising at 500 nm and 511 nm. Finally, the emission maxima of HBC **4** and HBC‐Pc **1** coincide at 481 nm; both exhibit each another distinguished emission band at 506 nm (HBC‐Pc **1**) and 511 nm (HBC **4**). In the emission spectra of all four compounds, a shoulder can be observed around 550 nm.

### Electrochemical Investigations

**Figure 5:**
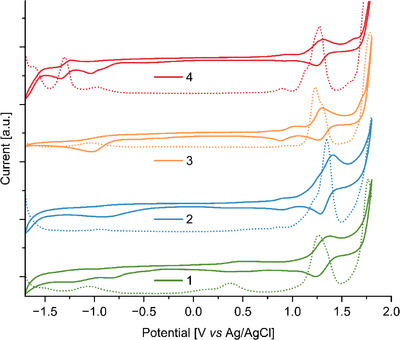
Cyclic voltammograms (CV – solid line) and differential pulse voltammograms (DPV – dashed line) of HBCs **1**‐**4** at 100 mV s^−1^ in deoxygenized 0.1 M *n*‐Bu_4_NPF_6_/DCM solution at 21 ± 1°C (V versus Ag/AgCl).

Experimental results were obtained from CV and DPV. Samples were prepared as solutions in DCM with 0.1 M *n*‐Bu_4_NPF_6_ as electrolyte *ve* *rsus* Ag/AgCl as reference electrode, Au working electrode and Pt counter electrode. The numerical results related to the first reduction and oxidation potentials are summarized in Table 2, while the voltammograms obtained for molecules **1‐4** are depicted in Figure 5. In general, it is noteworthy that the oxidations in all molecules are either of reversible or quasi‐reversible character, while the reductions tend to be irreversible, except for the second reduction of HBC **4**, which is associated with the nitro‐functionality and located at –1.30 V. The first reduction of HBC **4** as well as the two oxidations occur at –0.975, 0.898, and 1.28 V, respectively. A shoulder can be discerned at 1.13 V in the DPV spectrum. HBC **3**, which lacks nitro groups, produces only one observable reduction that is anodically shifted by 0.075 V with regard to HBC **4**, at ‐–1.05 V. However, the oxidations of HBCs **3** and **4** appear similarly at 0.944 V and 1.23 V with a shoulder visible in the DPV spectrum at 1.29 V. In case of HBC **2**, a cathodic shift of both the reduction potential and the second oxidation potential can be observed, as they occur at –0.930 V and 1.35 V, respectively. The first oxidation potential, however, experiences an anodic shift to 0.863 V. Furthermore, a shoulder can be distinguished in both the CV and DPV voltammograms at 1.22 V, again suggesting only quasi‐reversibility of the second oxidation. Finally, HBC‐Pc **1** experiences two reductions at –1.06 V and –0.839 V, a much lower first oxidation potential at 0.370 V as well as a second oxidation at a potential of 1.26 V, which again is analogous to those of the other HBC derivatives **2‐4**. Both the first reduction and oxidation are linked to the Ni‐phthalocyanine moiety.^[^ ^38^ ^]^

**Table 2:** First oxidation and reduction potentials of the analyzed compounds as measured by CV/DPV (V versus Ag/AgCl) in 0.1 M *n*‐Bu_4_NPF_6_/DCM at 21 ± 1°C, energy levels of the frontier orbitals calculated from the electrochemical values as well as electrical and calculated HOMO/LUMO gap.

Molecule:	**1**	**2**	**3**	**4**
E^ox^_1/2_ [V] ^a)^	0.370	0.863	0.944	0.898
E^red^_1/2_ [V] ^a)^	−0.839	−0.930	−1.050	−0.975
E_HOMO_ [eV] ^b)^	−4.974	−5.467	−5.548	−5.502
E_LUMO_ [eV] ^b)^	−3.765	−3.674	−3.554	−3.629
E_g_^elect^ [eV] ^c)^	1.209	1.793	1.994	1.873
E_g_^calc^ [eV] ^d)^	1.316	2.035	2.111	1.816

^a)^ Determined from cyclic voltammetry measurements versus (Ag/AgCl) reference electrode (0.164 V versus SHE at 20 °C);

^b)^ E_HOMO/LUMO_ [eV] = –(E^ox/red^_1/2_ + 4.604(*))^[^ ^57^, ^58^ ^]^ (*)calculation of conversion constant see Supporting Information;

^c)^ The electrical bandgap was calculated according to E_g_^elec^ [eV] = E_LUMO_ – E_HOMO_;

^d)^ determined from calculations of the ground states for all molecules.

Using these values, the experimental HOMO/LUMO gap is calculated. HBC‐Pc **1** shows the smallest gap of 1.209 eV, whereas those of HBCs **2‐4** lie at 1.793 eV (2), 1.994 eV (3), and 1.873 eV (4). Thus, the HOMO/LUMO gap of HBC‐Pc **1** is on average 0.67 eV smaller than those of the other compounds. The results determined by theoretical calculations are in agreement with the experimental ones and follow the same trend.

### Faraday Rotation Measurements

**Figure 6:**
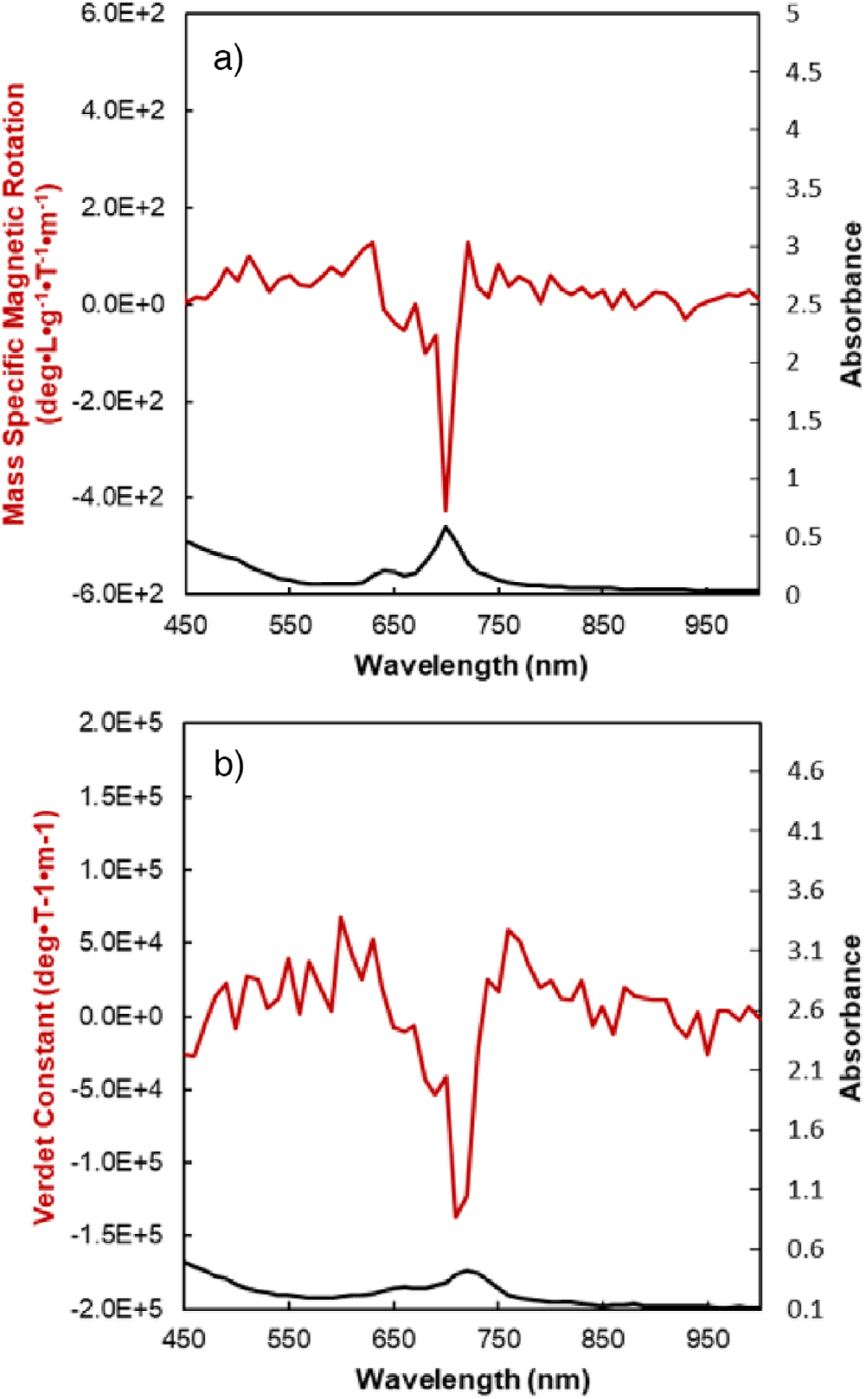
Faraday rotation (red) and absorption (black) spectra of HBC‐Pc **1** in a) toluene solution and b) thin film.

It has been shown in previous research that increased polarizability can lead to high Verdet constants, as was demonstrated in the case of ZnPc‐OT (Verdet constant of –3.3 × 10⁵ deg T^−1^m^−1^ at 800 nm).^[^ ^35^ ^]^ Therefore, we investigated the novel phthalocyanine derivative HBC‐Pc **1** bearing four HBC units as these large aromatic systems are expected to enhance polarizability through π‐extension. Measurements of HBC‐Pc **1** were conducted in both toluene solution and thin films (drop‐casted from chloroform, then vapor‐annealed). The compound exhibits strong Faraday rotation in both states, with a Verdet constant on the order of 10^5^ deg T^−1^ m^−1^ at 700 nm, corresponding to the maximum of the Q‐band and line shape consistent with a Faraday A‐term origin (Figure 6). This finding underscores the effectiveness of π‐extension in enhancing the magneto‐optical response of organic materials since it lies in the same order of magnitude as the previously published, π‐extended ZnPc‐OT and one order of magnitude higher than the conventional TGG (10^4^ deg T^−1^m^−1^ at 532 nm).^[^ ^34^ ^]^ Furthermore, the π‐extension of HBC‐Pc **1** enabled the Faraday Rotation absorption maximum to be located at 700 nm, whereas non‐π‐extended Pc derivatives with aliphatic side chains and comparable Verdet constants only absorb up to 640 nm.^[^ ^35^ ^]^ Thus, HBC‐Pc **1** not only exhibits a remarkable Verdet constant itself but also bridges a gap in absorption maxima location that previously existed in the field. The combination of high magneto‐optical activity, near‐infrared absorption, and straightforward preparation makes HBC‐Pc **1** an attractive candidate for integration into magneto‐optical devices, particularly in applications where low optical density is not required.

## Conclusion

For the first time, four hexabenzocoronene moieties have been connected to phthalocyanine in a single molecule, HBC‐Pc **1**, thus combining the optoelectronic properties of both scaffolds. UV–vis spectroscopy revealed strong absorption across the spectrum with local maxima at 371 nm (HBC β‐band) and 672 nm (Pc Q‐band). This allowed for the investigation of HBC‐Pc **1** as a Faraday rotator in a thin film as both solution‐processability and high absorption in the desired range are imperative. Upon irradiation at 700 nm, the compound revealed a remarkable Verdet constant of −1.4 × 10^5^ deg T^−1^m^−1^ consistent with the Faraday A‐term activity. The enhancement of the magneto‐optical response is attributed to the increased polarizability of the material due to the HBC moieties. As electrochemical measurements (CV and DPV) show, the connection of HBC and Pc building blocks leads to a decreased HOMO/LUMO gap of HBC‐Pc **1** compared to HBC‐based compounds **2‐4**. Values of E_g_^elect^  =  1.21 eV and E_g_^calc^  = 1.32 eV were determined experimentally or by DFT calculations, respectively. Additionally, the facile synthesis *via* a condensation reaction between HBC‐aldehyde **13** and a diamine allowed for the formation of benzodiimidazole‐bridged HBC dimer **2** and benzimidazole‐HBCs **3** and **4**. The latter two molecules contain a donor–acceptor motif; all compounds exhibit absorption extended up to 460 nm as well as slightly larger HOMO/LUMO gaps of 1.8–2.1 eV.

This highlights the usefulness of the approach, as a variety of HBC derivatives and therefore tunable material properties are easily accessible. With HBC‐Pc **1**, a new class of molecule is realized and its successful application as a Faraday rotator emphasizes the importance of this combination. HBC‐phthalocyanines offer a promising new class of hybrid molecules and are certain to be investigated further in the future, possibly with respect to amount of HBC moieties, linking units and direct fusion of HBC and Pc scaffolds.

## Supporting Information

The authors have cited additional references within the Supporting Information.^[^ ^48^, ^59^, ^60^, ^61^, ^62^, ^63^, ^64^, ^65^, ^66^ ^]^

## Conflict of Interests

The authors declare no conflict of interest.

## Supporting information



Supporting Information

## Data Availability

The data that support the findings of this study are available from the corresponding author upon reasonable request.
