# Supplementary material for: Hexabenzocoronene–Benzimidazole Hybrid Architectures and Faraday Rotation of the First Hexabenzocoronene–Phthalocyanine
Source: Angew Chem Int Ed Engl. 2025 Dec 16;65(5):e22494. doi: 10.1002/anie.202522494 (PMC12850995; doi:10.1002/anie.202522494)
Supplement: Supplementary file 1 — Supporting Information [file ANIE-65-e22494-s001.pdf]

Supporting Information

Hexabenzocoronene-Benzimidazole Hybrid Architectures and Faraday Rotation of the First Hexabenzocoronene-Phthalocyanine

Antonia Rocha-Ortiz,[a] Abdusalom A. Suleymanov,[b] Pascal Puhlmann,[c] Dustin Krischer,[a] Carolin Müller,[c] Dirk Zahn,[c] Timothy M. Swager [b] and Andreas Hirsch*[a]

Table of Contents

Materials and Instrumentational Methods.. 3

Syntheses.. 4

Characterization.. 15

Theoretical Investigation 56

Faraday Rotation Measurements... 62

Photographs: Solutions in Ambient Light and UV-Irradiation.. 63

References 63

Materials and Instrumentational Methods

All chemicals were purchased from commercial sources and used without further purification unless otherwise stated. Solvents were purified by distillation, except for THF which was purchased 99.9 % extra pure and anhydrous and used as received. Deuterated solvents were purchased from Deutero or Eurisotop; HPLC-grade solvents were purchased from VWR and used as obtained. Nitrogen of the brand LINDE with a purity of 99.9999 % or Argon served as inert gas.

Reactions under microwave irradiation were carried out in an Anton Paar Monowave 450 using Anton Paar G4, G10, or G30 microwave vials (30 bar max. pressure). Reaction mixtures were heated as fast as possible to the desired temperature with a maximum power of 850 W.

Thin layer chromatography (TLC) was performed on aluminium carrier foils coated with silica gel (Merck silica gel 60 F_{254}, 20 × 20 cm, film thickness 0.2 mm) and detected by UV-light (254 nm, 366 nm). Column and plug chromatography were performed on Machery-Nagel silica gel 60 M (230-400 mesh, 0.04–0.063 mm, deactivated). Flash chromatography was performed with a Biotage® Selekt system, SELEKT 1.1.1–13044 software and on Buchi FlashPure EcoFlex Silica columns of 12 g, 25 g and 40 g. Gel permeation size exclusion chromatography was performed on BioBeads SX1 from BioRad in $CHCl_3$.

NMR spectra were acquired with Bruker Ascend 400 (^{1}H: 400 MHz, ^{13}C: 101 MHz), 500 (^{1}H: 500 MHz, ^{13}C: 126 MHz) and 600 (^{1}H: 600 MHz, ^{13}C: 151 MHz) spectrometers. Chemical shifts (δ) are reported in ppm, referenced to residual protic solvent signals or the deuterated solvent itself (^{1}H: $CHCl_3$: 7.24 ppm, CH_2Cl_2: 5.32 ppm, $C_2H_2Cl_4$: 5.91 ppm, THF-d_8: 3.58 ppm; ^{13}C{^{1}H}: $CDCl_3$: 77.16 ppm, CD_2Cl_2: 53.40 ppm, $C_2D_2Cl_4$: 74.20 ppm, THF-d_8: 66.57 ppm). The resonance multiplicities are indicated as s (singlet), bs (broad singlet), d (doublet), t (triplet) and m (multiplet).

Mass spectrometry was carried out in either ESI, APPI or MALDI-TOF mode. ESI/APPI-ToF MS and high resolution MS (HRMS) was performed on a Bruker maXis 4G UHR TOF MS/MS spectrometer or a Bruker micrOTOF II focus TOF MS spectrometer. MALDI-MS spectra were recorded on a Shimadzu AXIMA Confidence MALDI-TOF using dhb (2,5-dihy-droxybenzoic acid), dctb (trans-2-[3-(4-tert-butylphenyl)-2-methyl-2-propenyliden]malononitrile) as a matrix, or without matrix (om); HRMS spectra were recorded on an LDI/ MALDI-ToF Bruker UltrafleXtreme machine.

UV/vis spectroscopy was carried out on a VarianCary 5000 UV–vis–NIR spectrometer in HPLC-grade solvents and room temperature using quartz cuvettes with a path length of 10 mm. A baseline correction was done prior to measurement. Fluorescence spectra were recorded on a FluoroMax 4 spectrofluorophotometer (Horiba) using the same equipment and conditions as for UV-vis spectroscopy. Emission maxima λ_{max} are given in nanometer [nm].

Electrochemical measurements were conducted in a classical three-electrode cell from Deutsche Metrohm GmbH & Co. KG, which was connected to Metrohm Autolab PGSTAT 101, controlled by NOVA 2.1 software, running on a personal computer. As a working electrode, a motionless gold electrode tip (0.03 cm^2) was used combined with a platinum sheet (1.0 cm^2) that served as a counter electrode. All potentials are presented relative to an Ag/AgCl (2 M lithium chloride in ethanol) reference electrode with a potential of 0.164 V vs SHE at 21 ± 1 °C, as given by the manufacturer. SHE is reported with a value of -4.44 eV on the Fermi scale, which therefore leads to a value of 4.604 eV for the calculation of the

HOMO/LUMO energy levels from the CV measurement data: $E_{HOMO/LUMO}$ [eV] = – ($E^{ox/red}{}_{1/2}$ + 4.604).[57,58] Spectra were recorded in dichloromethane (HPLC grade) at 21 ± 1 °C with 0.1 M n-Bu_4NPF_6 as a supporting electrolyte. For cyclic voltammetry, two different scan rates of v = 100 mVs^{-1} was chosen, whereas differential pulse voltammetry was conducted with a scan rate of v = 10 mVs^{-1}. DCM was deoxygenated with nitrogen (1 min/mL) before each measurement. The nitrogen atmosphere was maintained during all measurements.

Syntheses

Mesityl-substituents are abbreviated as follows:

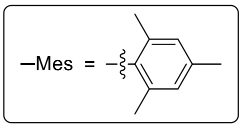

Target Molecules

Tetra-HBC Ni-Phthalocyanine (1)

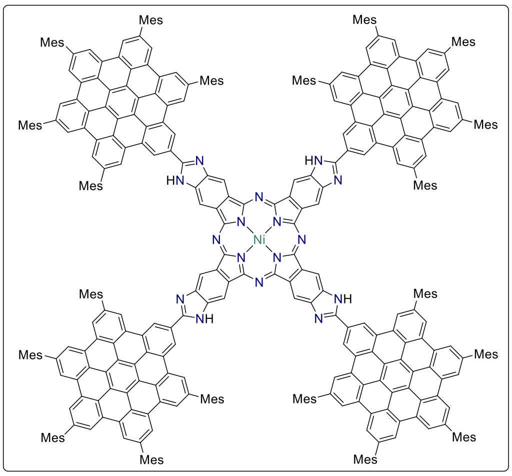

Under inert atmosphere, HBC **3** (85.0 mg, 6.64×10^{-5} mol, 4.0 eq.) and $NiCl_2$ (6.50 mg, 4.98×10^{-5} mol, 3.0 eq.) were dissolved in dry 1-pentanol (1.0 mL) and degassed with Ar. Two drops of DBU (10 µL) were added and the reaction mixture was heated to 125 °C for 14 d, during which a colour change towards green occurred and gradually intensified. The reaction was ended *via* cooling to room temperature and consecutive plug filtration (SiO_2, DCM + 1 % triethyl amine (TEA)). The product was obtained after gel permeation chromatography (BioBeads SX1, $CHCl_3$) as a dark green solid (78.0 mg, 1.51×10^{-5} mol, 91 %).

m.p.: continuous decomposition >138 °C;

^{1}H NMR ($C_2D_2Cl_4$, 500 MHz, 130 °C): δ = 9.59 (s, 8H), 9.06 + 9.02 + 8.83 + 8.77 (4×s, 48H), 7.08 + 7.00 + 6.94 (3×s, 40H), 2.42 (s, 24H), 2.31+2.27 (2×s, 96H), 2.12+2.09 (2×s, 60H) ppm;

^{13}C NMR (CD_2Cl_2, 151 MHz, RT): δ = 140.9, 140.7, 139.5, 139.3, 137.5, 136.5, 136.3, 135.7, 132.3, 131.6, 131.4, 131.3, 128.7, 128.5, 127.6, 124.7, 124.2, 122.3, 121.9, 121.5, 114.1, 21.4, 21.3, 21.2, 19.9, 19.3 ppm; ($C2D_2Cl_4$, 126 MHz, 110 °C): δ = 141.0, 139.4, 137.3, 137.2, 136.7, 136.3, 136.3, 136.3, 136.2, 131.58, 131.5, 131.5, 131.4, 131.3, 131.0, 129.0, 128.8, 128.7, 124.3, 124.2, 122.1, 121.8, 21.6, 21.5, 21.4, 21.3, 21.3, 21.2 ppm;

IR (ATR, diamond, cm^{-1}): ṽ = 3647, 3066, 3008, 2952, 2921, 2852, 2325, 2186, 2112, 1918, 1722, 1611, 1580, 1547, 1485, 1455, 1437, 1402, 1376,1362, 1259, 1089, 1051, 1029, 877, 849, 752;

UV-vis (DCM, rt): λ_{max}(HBC) = 371 nm (3.24×10^5 L*mol^{-1}*cm^{-1}), λ_{max}(Pc) = 672 nm (0.55×10^5 L*mol^{-1}*cm^{-1});

Fluorescence (DCM, nm): λ_{ex} = 371, λ_{em} = 481;

MALDI MS: m/z calculated for $C_{384}H_{280}N_{16}Ni$ [M]+: m/z = 5177.2694, found m/z = 5177.3480.

Di-HBC-benzodiimdazole (2)

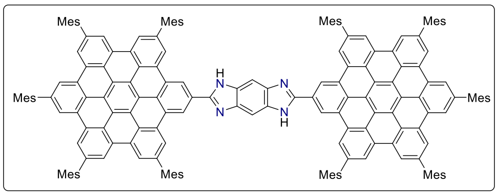

Under inert atmosphere, 1,2,4,5-tetraaminobenzene tetrahydrochloride (15.0 mg, 5.28×10^{-5} mol, 1.0 eq.) was dissolved in dry DMF (2.0 mL) and TEA (18 µL, 12.7×10^{-5} mol, 2.4 eq.) was added. The mixture was stirred under light exclusion and Ar for 30 min, before excessive TEA was removed under vacuum. Now, HBC aldehyde **13** (151 mg, 13.2×10^{-5} mol, 2.5 eq.) and $Zn(OTf)_2$ (4.81 mg, 1.32×10^{-5} mol, 0.25 eq.) were added and the mixture was degassed briefly with Ar. The reaction was carried out under microwave irradiation at 170 °C for 15 h. After cooling to room temperature, the reaction mixture was filtered through silica and, after solvent evaporation, purified *via* flash column chromatography, column chromatography (SiO_2, DCM + 1 % acetone), gel permeation chromatography (BioBeads SX1, $CHCl_3$), repeated column chromatography (SiO_2, DCM) and finally precipitated from chloroform with a mixture of pentane and methanol to obtain the product as a bright yellow solid (38.9 mg, 1.64×10^{-5} mol, 31 %).

$\mathbf{R_f}$ = 0.74 (DCM 100 %);

m.p. = decomposition >330 °C;

^{1}H NMR (THF-d_8, 500 MHz, 60 °C): δ = 12.25 (s, 2H), 12.09* (s), 10.25 (s, 4H), 10.21* (s), 9.41* (s), 9.40+9.22+9.19+9.17+9.17 (5×s, 20H), 8.22* (s), 7.90 (s, 2H), 7.49* (s), 7.15 (s, 8H), 7.05+7.04 (2×s, 12H), 2.47 (s, 12H), 2.37+2.36 (2×s, 18H), 2.28 (s + H_2O signal), 2.22 (s, 36H) ppm;

THF-d_8, 500 MHz, -10 °C): δ = 12.54 (s, 2H), 12.41* (s), 10.31 (s, 4H), 10.27* (s), 9.45* (s), 9.42+9.34+9.29+9.28+9.27 (5×s, 20H), 8.21* (s), 7.93 (s, 2H), 7.48* (s), 7.18 (s, 8H), 7.07+7.06 (2×s, 12H), 2.49 (s, 12H), 2.38+2.37 (2×s, 18H), 2.29 (s, 24H), 2.23 (s, 36H) ppm;

* Indicates signals arising from protons of the *cis*-isomer

^{13}C NMR (THF, 126 MHz, 60 °C): δ = 153.3, 144.5, 141.8, 141.7, 141.7, 141.0, 140.5, 140.3, 140.3, 137.8, 137.6, 137.1, 136.9, 134.7, 132.4, 132.4, 132.2, 132.2, 132.2, 132.1, 130.8, 129.1, 129.0, 127.3, 125.7, 125.5, 124.9, 124.8, 124.6, 122.6, 122.5, 122.3, 122.0, 121.2, 21.4, 21.4, 21.4, 21.2, 21.1 ppm;

IR (ATR, diamond, cm^{-1}): ṽ = 3648, 3366, 3066, 3020, 2951, 2917, 2853, 2285, 2114, 2084, 1916, 1884, 1729, 1702, 1609, 1579, 1555, 1484, 1437, 1376, 1359, 1262, 1250, 1030, 1014, 876, 848, 751, 736;

UV-vis (DCM, rt) λ_{max}(HBC) = 371 nm (2.32×10^5 L*mol^{-1}*cm^{-1}), λ_2 = 433 nm (1.21×10^5 L*mol^{-1}*cm^{-1});

Fluorescence (DCM, nm): λ_{ex} = 371, λ_{em} = 476;

MALDI MS: m/z calculated for $C_{182}H_{138}N_4$ [M]+: m/z = 2379.0922, found m/z = 2379.0489.

HBC-imidazophthalonitrile (3)

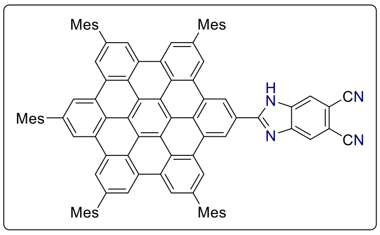

Under inert atmosphere, HBC aldehyde **13** (100 mg, 8.76×10^{-5} mol, 1.0 eq.), 4,5-dinitrophthalonitrile (21.0 mg, 13.2×10^{-5} mol, 1.5 eq.) and $Zn(OTf)_2$ (3.20 mg, 0.876×10^{-5} mol, 0.1 eq.) were dissolved in dry DMF (0.7 mL) and degassed with Ar. The reaction was carried out under microwave irradiation at 170 °C for 8 h. After cooling to room temperature, the reaction mixture was filtered through silica and, after solvent evaporation, purified *via* flash column chromatography (SiO_2, hexanes:DCM 1:9 → 0:1. The product was obtained as a yellow solid (49.3 mg, 3.85×10^{-5} mol, 44 %).

R$_f$ = 0.32 (DCM 100 %);

m.p. = decomposition >310 °C;

^{1}H NMR ($C_2D_2Cl_4$, 500 MHz, 45 °C): δ = 10.57 (s, 1H), 9.88 (s, 2H), 9.20 (s, 2H), 9.06+9.01+8.99 (3×s, 8H), 8.30 (s, 1H), 7.98 (s, 1H), 7.09+7.02+7.01 (3×s, 10H), 2.40+2.35+2.34 (3×s, 15H), 2.19+2.16+2.16 (3×s, 30H) ppm;

^{13}C NMR ($C_2D_2Cl_4$, 126 MHz, 45 °C): δ = 157.6, 146.9, 141.0, 140.9, 140.8, 139.1, 139.0, 137.8, 137.4, 136.8, 136.6, 136.3, 136.2, 132.1, 131.5, 131.4, 131.3, 131.0, 130.5, 128.9, 128.8, 128.3, 126.5, 126.0, 124.9, 124.8, 124.5, 124.4, 124.2, 123.7, 122.6, 122.0, 121.7, 120.7, 117.4, 117.0, 109.6, 109.3, 74.4, 74.2, 74.0, 21.6, 21.6, 21.5 ppm;

IR (ATR, diamond, cm^{-1}): $\tilde{\nu}$ = 3067, 3025, 2951, 2915, 2855, 2232, 2081, 1728, 1610, 1540, 1437, 1366, 1315, 1257, 1085, 1013, 873, 848;

UV-vis (DCM, rt): λ_{max}(HBC) = 373 nm (1.58×10^5 L*mol^{-1}*cm^{-1});

Fluorescence (DCM, nm): λ_{ex} = 373, λ_{em} = 479;

MALDI HRMS: m/z calculated for $C_{96}H_{70}N_4$ [M]+: m/z = 1278.5600, found m/z = 1278.5595.

HBC-dinitrobenzimidazole (4)

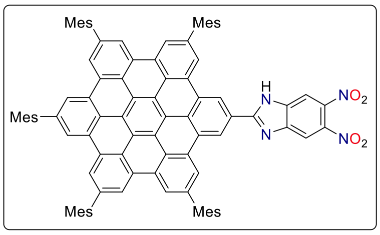

Under inert atmosphere, HBC aldehyde **13** (100 mg, 8.76×10^{-5} mol, 1.0 eq.), 1,2-diamino-4,5-dinitrobenzene (26.2 mg, 13.2×10^{-5} mol, 1.5 eq.) and $Zn(OTf)_2$ (3.20 mg, 0.876×10^{-5} mol, 0.1 eq.) were dissolved in dry THF (0.7 mL) and degassed with Ar. The reaction was carried out under microwave irradiation at 120 °C for 7 h. After cooling to room temperature, the reaction mixture was filtered through silica and, after solvent evaporation, purified *via* flash column chromatography (SiO_2, hexanes:DCM 1:1 → 3:7 → 0:1 (v/v)). The product was obtained as yellow solid (37.0 mg, 2.80×10^{-5} mol, 32 %).

R$_f$ = 0.36 (DCM 100 %);

m.p. = decomposition >330 °C;

^{1}H NMR ($C_2D_2Cl_4$, 400 MHz, RT): δ = 10.87 (s, 1H), 9.89 (s, 2H), 9.19 (s, 2H), 9.06+9.00+8.99+8.98 (4×s, 8H), 8.38 (s, 1H), 8.14 (s, 1H), 7.09+7.05+7.01 (3×s, 10H), 2.39+2.36+2.33 (3×s, 15H), 2.17+2.15+2.14 (3×s, 30H) ppm;

^{13}C NMR ($C_2D_2Cl_4$, 101 MHz, RT): δ = 158.8, 146.1, 141.0, 140.8, 140.8, 140.2, 139.3, 139.1, 139.0, 137.8, 137.4, 136.7, 136.3, 136.3, 135.6, 132.1, 131.5, 131.3, 131.3, 130.9, 130.4, 128.9, 128.8, 125.9, 124.9, 124.4, 124.2, 123.9, 122.0, 121.7, 120.6, 117.5, 109.2, 21.7, 21.7, 21.7, 21.6 ppm;

IR (ATR, diamond, cm^{-1}): $\tilde{\nu}$ = 3066, 3005, 2950, 2916, 2855, 2324, 2087, 1770, 1609, 1539, 1440, 1366, 1340, 1081, 1033, 874, 849, 750;

UV-vis (DCM, rt): λ_{max}(HBC) = 372 nm (1.22×10^5 L*mol^{-1}*cm^{-1});

Fluorescence (DCM, nm): λ_{ex} = 372, λ_{em} = 481;

MALDI HRMS: m/z calculated for $C_{94}H_{70}N_4O_4$ [M]+: m/z = 1318.5397, found m/z = 1318.5392.

Precursor Molecules

The syntheses of precursors **5**, **7**, **9**, **10**, **14** and **15**, were adapted from procedures reported in literature as referenced at the respective molecule. Each compounds´ spectroscopic data matched those previously reported. Precursor molecules **6**, **8**, **11-13**, **16** and **17** are not reported previously, however, their syntheses was performed according to reported procedures of similar molecules, as referenced.

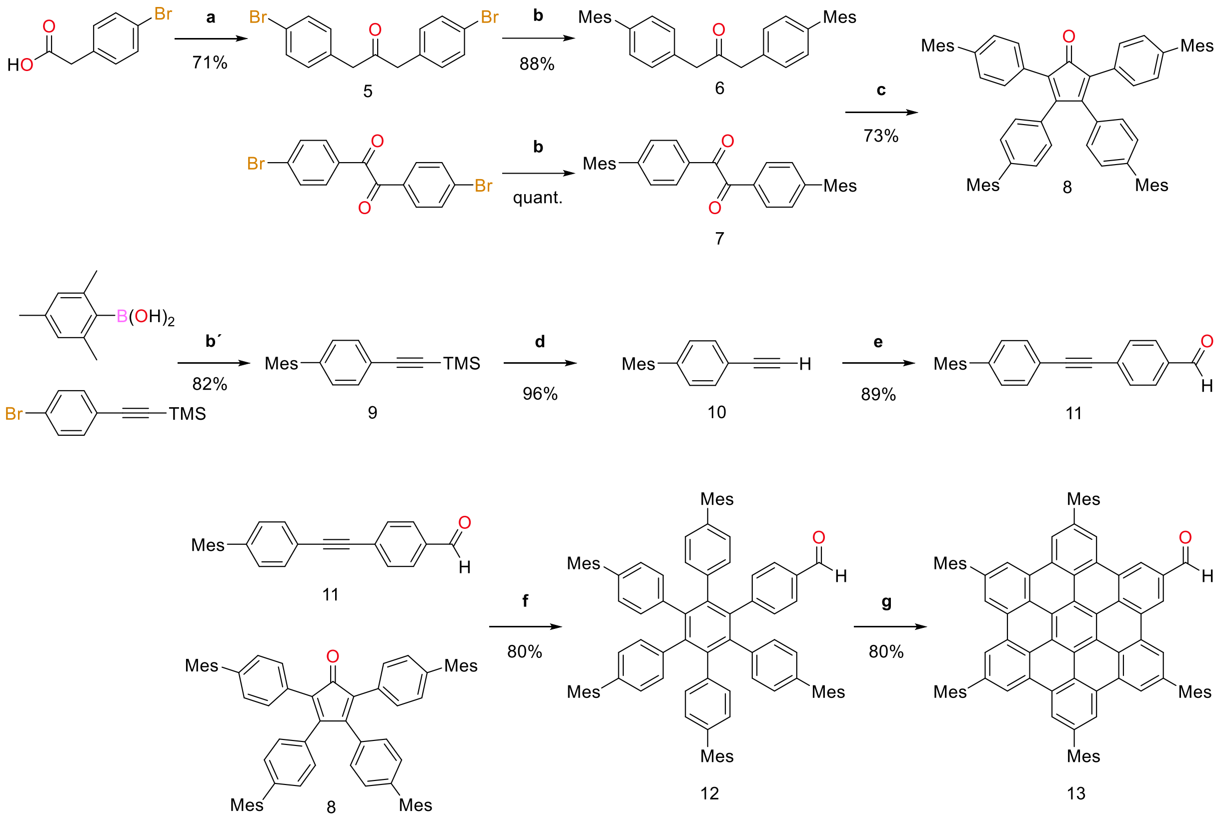

Scheme S1: Synthetic approach towards precursors 5-13: a) DCC, DMAP, DCM, rt, 18 h; b) Na_2CO_3, TBAB, Mes-B(OH)$_2$, Pd(PPh$_3$)$_4$, tol, EtOH, H_2O, 100°C, 66-68 h; b´) Na_2CO_3, TBAB, Pd(PPh$_3$)$_4$, tol, EtOH, H_2O, 100°C, 17 h; c) KOH, EtOH, 79°C, 3 h; d) K_2CO_3, MeOH, rt, 3 h, NH_4Cl; e) Pd(PPh$_3$)$_2$Cl$_2$, CuI, DIPA, THF, rt, 22 h; f) Ph_2O, 250°C, 18 h; g) DDQ, TfOH. DCM, 0°C to rt, 3 h.

1,3-Bis(4-bromophenyl)acetone (5)[59]

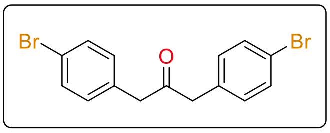

Under inert atmosphere, in a Schlenk flask equipped with a dropping funnel, DCC (7.70 g, 37.5 mmol, 1.0 eq.) and DMAP (1.15 g, 9.40 mmol, 0.25 eq.) were dissolved in anhydrous DCM (75 mL) and degassed for 10 min. A solution of 2-(4-bromophenyl)acetic acid (8.05 g, 37.5 mmol, 1.0 eq.) in degassed, anhydrous DCM (75 mL) was filled into the dropping funnel and added to the DCC/DMAP solution dropwise over 2 h. The reaction was monitored *via* TLC (SiO_2, hexanes/ethyl acetate, 9:1 v:v), which showed complete turnover of the reactant after 18 h. The solvent was removed *in vacuo* and the product was obtained by flash column chromatography (SiO_2, hexanes/ethyl acetate, 9:1 v:v) as colourless crystalline solid (4.86 g, 13.2 mmol, 71 %).

R$_f$ = 0.37 (hexanes/ethyl acetate 85:15);

m.p. = 120 °C;

^{1}H NMR (400 MHz, $CDCl_3$) δ (ppm): 7.44-7.41 (4H, m, ar-H), 7.01-6.98 (4H, m, ar-H), 3.66 (4H, s, CH_2);
^{13}C NMR: (101 MHz, $CDCl_3$) δ (ppm): 204.4, 132.7, 132.0, 131.3, 121.4, 48.6;
HRMS (APPI, DCM) [M+H]+: m/z calc.: ($C_{15}H_{12}Br_2O$): 366.9328, found: 366.9326.

1,3-Bis(4-mesitylphenyl)propan-2-one (6)[48]

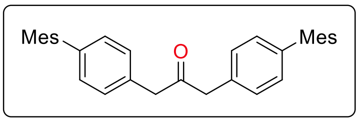

Ketone **5** (2.00 g, 5.40 mmol, 1.0 eq.), Na_2CO_3 (2.30 g, 21.7 mmol, 4.0 eq.) and TBAB (0.220 g, 0.680 mmol, 0.13 eq.) were dissolved and degassed for 15 min in a mixture of toluene (20 mL), ethanol (5.0 mL) and H_2O (2.0 mL). Mesitylboronic acid (2.10 g, 12.8 mmol, 2.4 eq.) was added and the solution was further degassed for 15 min. Next, $Pd(PPh_3)_4$ (0.400 g, 0.350 mmol, 0.06 eq.) was added and the reaction mixture was heated to 100 °C for 68 h. After cooling to room temperature, the solvents were removed *in vacuo*, followed by extraction with DCM, washing with H_2O and drying over $MgSO_4$. The product was obtained after flash column chromatography (SiO_2, hexanes/ethyl acetate, 1:0 → 85:15 v:v) as colourless crystalline solid (2.12 g, 4.75 mmol, 88 %).
R_f = 0.66 (hexanes/ethyl acetate 85:15);
m.p. = 158 °C;
^{1}H NMR: (400 MHz, $CDCl_3$) δ (ppm): 7.20-7.17 (4H, m, ar-H), 7.08-7.05 (4H, m, ar-H), 6.91 (4H, s, ar-H), 3.80 (4H, s, CH_2), 2.30 (6H, s, CH_3), 1.98 (12H, s, CH_3);
^{13}C NMR: (101 MHz, $CDCl_3$) δ (ppm): 206.2, 140.0, 138.7, 136.8, 136.1, 132.3, 129.8, 129.8, 128.2, 49.2, 21.2, 20.9;
HRMS (APPI, DCM) [M+H]+: m/z calc.: ($C_{33}H_{34}O$): 447.2682, found: 447.2681.

4,4`-Dimesitylbenzil (7)[48]

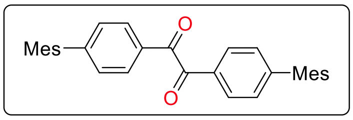

4,4´-Dibromobenzil (2.00 g [purchased: 90 % purity], 4.86 mmol, 1.0 eq.) and mesitylboronic acid (2.10 g, 12.8 mmol, 2.6 eq.) were dissolved and degassed for 10 min a mixture of toluene (20 mL), ethanol (5.0 mL) and H_2O (2.0 mL). Afterwards, TBAB (0.220 g, 0.680 mmol, 0.14 eq.) was added and the solution was degassed for additional 10 min. Next, Na_2CO_3 (2.30 g, 21.7 mmol, 4.5 eq.) and $Pd(PPh_3)_4$ (0.400 g, 0.350 mmol, 0.07 eq.) were added, the reaction mixture was again degassed for 10 min and then heated to 100 °C. After 66 h, monitoring *via* TLC (SiO_2, hexanes/DCM, 7:3 v:v) showed a considerable amount of mono-mesitylated intermediate, wherefore additional mesitylboronic acid (0.700 g, 4.27 mmol, 0.88 eq.) and $Pd(PPh_3)_4$ (0.135 g, 0.117 mmol, 0.02 eq.) were added. After 72 h the reaction was let cool to room temperature and the solvent removed *in vacuo*. The residue was extracted with DCM, washed with H_2O, dried over $MgSO_4$ and purified by means of flash column chromatography (SiO_2, hexanes/DCM, 8:2 → 7:3 v:v). to obtain the product was as yellow crystalline solid (2.17 g, 4.86 mmol, quant.).
R_f = 0.29 (hexanes/DCM 7:3);
m.p. = 182 °C;
^{1}H NMR (400 MHz, $CDCl_3$) δ (ppm): 8.09-8.06 (4H, m, ar-H), 7.33-7.30 (4H, m, ar-H), 6.95 (4H, s, ar- H), 2.32 (6H, s, CH_3), 1.99 (12H, s, CH_3);

^{13}**C NMR** (101 MHz, $CDCl_3$) δ (ppm): 194.4, 148.8, 137.7, 137.6, 135.5, 131.6, 130.4, 130.4, 128.4, 21.2, 20.8;
HRMS (APPI, DCM) [M+H]+: m/z calc.: ($C_{32}H_{30}O_2$): 447.2319, found: 447.2313.

2,3,4,5-Tetrakis-(4´-mesitylphenyl)-cyclopenta-2,4-dien-1-one (8)[60]

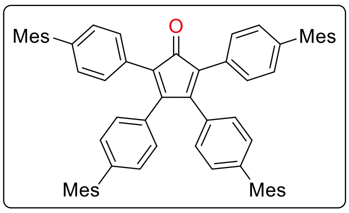

In a round-bottom flask under ambient conditions, ketone **6** (2.00 g, 4.48 mmol, 1.0 eq.) and benzil **7** (2.00 g, 4.48 mmol, 1.0 eq.) were suspended in ethanol (30 mL) and heated to until dissolved. Then, KOH (0.126 g, 2.24 mmol, 0.50 eq.) dissolved in ethanol (3.0 mL) was added and the reaction was stirred at reflux for 3 h. After cooling to room temperature, the mixture was purified by recrystallization from ethanol and the product obtained as purple crystalline solid (4.81 g, 3.28 mmol, 73 %).
m.p. = 220 °C;
^{1}**H NMR:** (400 MHz, $CDCl_3$) δ (ppm): 7.41-7.39 (4H, m, ar-H), 7.07-7.02 (8H, m, ar-H), 6.98-6.96 (4H, m, ar-H), 6.92 + 6.91 (8H, 2 × s, ar-H), 2.31 + 2.30 (12H, 2 × s, CH_3), 2.00 (12H, s, CH_3), 1.93 (12H, s, CH_3);
^{13}**C NMR** (101 MHz, $CDCl_3$): δ (ppm): 201.1, 155.0, 141.5, 140.4, 138.8, 138.6, 137.0, 136.8, 136.1, 135.8, 132.0, 130.2, 129.5, 129.3, 129.2, 129.1, 128.2, 128.2, 125.1, 21.2, 20.9, 20.5;
HRMS (MALDI-dctb): m/z Calc.: ($C_{65}H_{60}O$): 856.4639, found: 856.4647.

((4-Mesitylphenyl)ethynyl)trimethylsilane (9)[48]

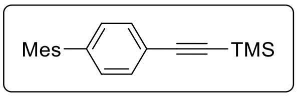

((4-Bromophenyl)ethynyl)trimethylsilane (1.00 g, 3.95 mmol, 1.0 eq.), TBAB (0.166 g, 5.14 mmol, 0.13 eq.) and Na_2CO_3 (1.05 g, 9.88 mmol, 2.5 eq.) were dissolved in a mixture of in toluene (20 mL), ethanol (5.0 mL) and H_2O (2.0 mL) and degassed for 15 min. Then, mesitylboronic acid (0.777 g, 4.74 mmol, 1.2 eq.) was added and the solution was again degassed for 10 min before, $Pd(PPh_3)_4$ (0.274 g, 0.237 mmol, 0.06 eq.) was added. The reaction mixture was heated to 100 °C for 17 h. After cooling to room temperature, the solution was diluted with ethanol (10 mL) and the solvent removed by rotary evaporation. The residue was extracted with DCM, washed with H_2O and brine and purified *via* flash column chromatography (SiO_2, hexanes 100 %). The product was obtained as colourless crystalline solid (0.714 g, 3.24 mmol, 82 %).
R_f = 0.42 (hexanes/DCM 95:5);
m.p. = 84 °C;
^{1}**H NMR** (400 MHz, $CDCl_3$) δ (ppm): 7.52-7.49 (2H, m, ar-H), 7.08-7.05 (2H, m, ar-H), 6.91 (2H, s, ar-H), 2.30 (3H, s, CH_3), 1.95 (6H, s, CH3), 0.25 (9H, s, CH_3);
^{13}**C NMR** (101 MHz, $CDCl_3$): δ (ppm): 141.8, 138.5, 137.0, 135.9, 132.2, 129.5, 128.3, 121.4, 105.3, 94.2, 21.2, 20.8, 0.16;
HRMS (APPI, DCM) [M+H]+: m/z calc.: ($C_{20}H_{24}Si$): 292.1642, found: 292.1648.

(4-Mesitylphenyl)acetylene (10)[61]

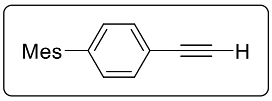

Acetylene **9** (0.700 g, 2.39 mmol, 1.0 eq.) was dissolved in MeOH (35 mL) and degassed for 15 min before K_2CO_3 (0.264 g, 1.91 mmol, 0.8 eq.) was added. The reaction was ended after 3 h *via* addition of NH_4Cl until an acidic pH-value was achieved. The crude mixture was extracted with DCM, the combined organic phases were washed with H_2O and dried over $MgSO_4$. Finally, the solvent was removed *in vacuo* and the product was obtained as colourless liquid that solidifies upon cooling to -20 °C (0.506 g, 2.29 mmol, 96 %).

R_f = 0.60 (hexanes/DCM 4:1);

m.p. = 43 °C;

^{1}H NMR: (400 MHz, CD_2Cl_2) δ (ppm): 7.56-7.53 (2H, m, ar-H), 7.12-7.09 (2H, m, ar-H), 6.93 (2H, s, ar-H), 3.15 (1H, s, H-acet.), 2.31 (3H, s, CH_3), 1.97 (6H, s, CH_3);

^{13}C NMR: (101 MHz, CD_2Cl_2) δ (ppm): 142.5, 138.5, 137.2, 135.9, 132.5, 130.0, 128.5, 120.6, 77.3, 21.1, 20.8;

HRMS (APPI, DCM) [M+H]+: m/z calc.: ($C_{17}H_{16}$): 221.1325, found: 221.1320.

4-((4-Mesitylphenyl)ethynyl)benzaldehyde (11)[7]

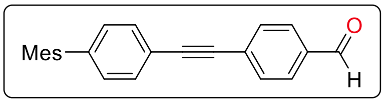

In a 50 mL Schlenk flask, 4-iodobenzaldehyde (0.400 g, 1.72 mmol, 1.00 eq.), CuI (0.0164 g, 0.0860 mmol, 0.05 eq.), $Pd(PPh_3)_2Cl_2$ (0.0604 g, 0.0860 mmol, 0.05 eq.) and diisopropylamine (1.20 mL, 0.872 g, 8.62 mmol, 5.00 eq.) were dissolved in THF (20 mL) and degassed *via* sonication (2 × 3 min) and purged with argon. The reaction was started by addition of molecule **10** (0.400 g, 1.82 mmol, 1.05 eq.) in solid state and one portion and stirred at room temperature for 22 h. The solvent was evaporated *in vacuo* and the reaction mixture was purified *via* flash column chromatography (SiO_2, hexanes/DCM, 8:2 → 7:3 v:v) to obtain the product as a colourless crystalline solid (0.498 g, 1.54 mmol, 89 %).

R_f = 0.13 (hexanes/DCM 7:3);

m.p. = 161 °C;

^{1}H NMR: (400 MHz, $CDCl_3$) δ (ppm): 10.02 (1H, s, CHO), 7.88-7.86 (2H, m, ar-H), 7.69-7.67 (2H, m, ar-H), 7.62-7.59 (2H, m, ar-H), 7.18-7.15 (2H, m, ar-H), 6.95 (2H, s, ar-H), 2.33 (3H, s, CH_3), 2.01 (6H, s, CH_3);

^{13}C NMR: (101 MHz, $CDCl_3$) δ (ppm): 191.5, 142.3, 138.3, 137.1, 135.8, 135.5, 132.2, 132.0, 129.8, 129.7, 129.7, 128.3, 120.8, 93.7, 88.6, 21.2, 20.8;

HRMS (APPI, DCM) [M+H]+: m/z calc.: ($C_{24}H_{20}O$): 324.1509, found: 324.1512.

Pentamesityl-HPB-aldehyde (12)[61]

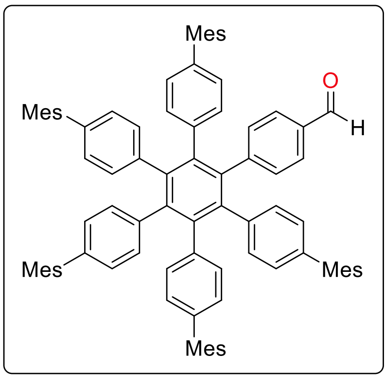

In a microwave pressure vial, cyclopentadienone **8** (1.14 g, 1.33 mmol, 1.0 eq.) and acetylene **11** (0.432 g, 1.33 mmol, 1.0 eq.) were dissolved in Ph_2O (4.66 mL, 4.99 g, 29.3 mmol, 22 eq.) and degassed for 15 min. The reaction was carried out at 250° C for 18 h. After cooling to room temperature, the product was precipitated from the mixture with cold methanol and obtained as off-white solid (1.24 g, 1.07 mmol, 80 %).

m.p. = decomposition >318 °C;

^{1}H NMR (500 MHz, $CDCl_3$) δ (ppm): 9.76 (1H, s, CHO), 7.40-7.38 (2H, m, ar-H), 7.10-7.08 (2H, m, ar-H), 7.02-6.99 (6H, m, ar-H), 6.94-6.91 (4H, m, ar-H), 6.84 + 6.82 (10H, 2 × s, ar-H), 6.70-6.63 (4H, m, ar-H), 2.27 + 2.26 (15H, 2 × s, CH_3), 1.78 + 1.75 (24H, 2 × s, CH_3), 1.68 (6H, s, CH_3);

^{13}C NMR: (126 MHz, $CDCl_3$) δ (ppm): 192.0, 148.4, 141.1, 140.6, 140.4, 139.9, 139.1, 139.1, 139.0, 138.9, 138.9, 138.8, 138.5, 138.4, 138.3, 136.5, 136.5, 136.4, 136.0, 136.0, 135.7, 133.5, 132.5, 132.0, 132.0, 131.8, 128.2, 128.0, 127.9, 127.9, 127.8, 127.8, 127.6, 21.2, 20.6, 20.5, 20.4;

HRMS (MALDI-dctb): m/z Calc.: ($C_{88}H_{80}O$): 1152.6204, found: 1152.6223.

Pentamesityl-HBC-aldehyde (13)[61]

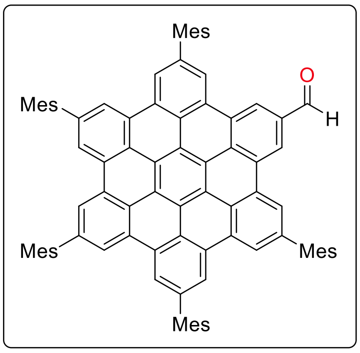

Under inert atmosphere, HPB **12** (0.300 g, 0.260 mmol, 1.0 eq.) and DDQ (0.413 g, 1.82 mmol, 7.0 eq.) were dissolved in HPLC-grade DCM (50 mL), cooled to 0 °C and degassed for 15 min. Then, TfOH (0.32 mL, 0.546 g, 3.64 mmol, 14 eq.) was added and the reaction was stirred for 3 h while gradually warming to room temperature. It was ended *via* the addition of TEA (9.0 mL). The solvent was removed *in vacuo* and the residue was purified by plug chromatography (SiO_2, hexanes/DCM, 2:3 v:v) to yield the product is orange flakes (0.237 g, 0.208 mmol, 80 %).

R_f = 0.44 (hexanes/DCM 7:3);

m.p. = decomposition >320 °C;

^{1}H NMR (400 MHz, $CDCl_3$) δ (ppm): 10.57 (1H, s, CHO), 9.68 (2H, s, ar-H), 9.21-9.00 (10H, m, ar-H), 7.14 + 7.08 (10H, 2 × s, ar-H), 2.46 + 2.41 (15H, 2 × s, CH_3), 2.25 + 2.23 + 2.22 (30H, 3 × s, CH_3);

^{13}C NMR: (101 MHz, $CDCl_3$) δ (ppm): 193.0, 140.7, 140.7, 140.5, 139.0, 139.0, 137.6, 137.4, 136.3, 136.2, 134.0, 131.6, 131.3, 131.3, 131.1, 130.9, 130.7, 130.0, 128.6, 128.6, 124.8, 124.5, 124.5, 124.3, 124.0, 123.9, 123.2, 122.7, 122.4, 121.6, 120.7, 21.5, 21.5, 21.5, 21.5, 21.4, 21.3;

HRMS (MALDI-dctb): m/z Calc.: ($C_{88}H_{68}O$): 1140.5265, found: 1140.5259.

Reference Molecule

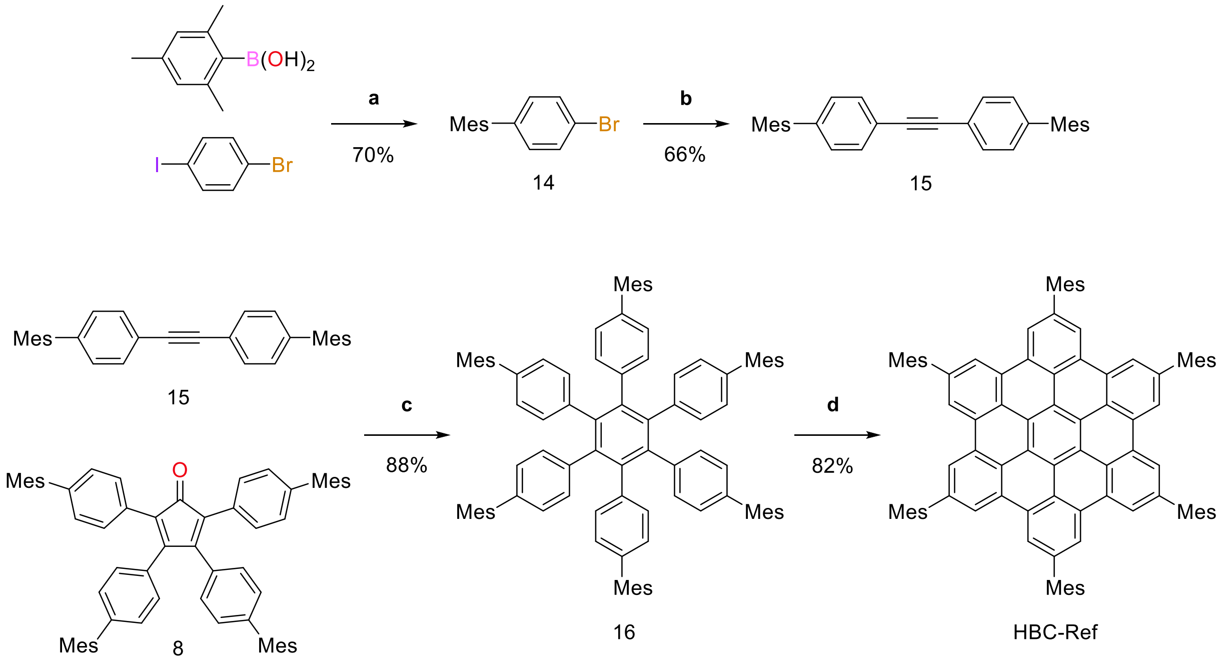

*Scheme S2: Synthetic approach towards precursors **14** - **HBC-Ref**: a) Na_2CO_3, TBAB, $Pd(PPh_3)_4$, tol, EtOH, H_2O, 100°C, 17 h; b) Acetylene **10**, $Pd(PPh_3)_2Cl_2$, CuI, TEA, 70°C, 22 h; c) Ph_2O, 250°C, 18 h; d) DDQ, TfOH. DCM, 0°C to rt, 3 h.*

4-Mesityl-1-bromobenzene (14)[48]

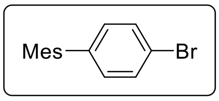

1-Bromo-4-iodobenzene (500 mg, 1.77 mmol, 1.0 eq.), TBAB (74.1 mg, 0.230 mmol, 0.13 eq.) and Na_2CO_3 (468 mg, 4.42 mmol, 2.5 eq.) were dissolved in a mixture of in toluene (20 mL), ethanol (5.0 mL) and H_2O (2.0 mL) and degassed for 15 min. Then, mesitylboronic acid (304 mg, 1.86 mmol, 1.05 eq.) was added and the solution was again degassed for 10 min before $Pd(PPh_3)_4$ (102 mg, 0.0880 mmol, 0.05 eq.) was added. The reaction mixture was heated to 100 °C for 17 h. After cooling to room temperature, the solution was diluted with ethanol (10 mL) and the solvent removed by rotary evaporation. The residue was extracted with DCM, washed with H_2O and brine and purified *via* plug filtration (SiO_2, hexanes/DCM ,95:5 v:v). The product was obtained as colourless crystalline solid (341 mg, 1.24 mmol, 70 %).

R_f = 0.55 (hexanes/DCM 95:5);

m.p. = 74 °C;

^{1}H NMR: (400 MHz, $CDCl_3$) δ (ppm): 7.54-7.51 (2H, m, ar-H), 7.02-6.98 (2H, m, ar-H), 6.92 (2H, s, ar-H), 2.31 (3H, s, CH_3), 1.97 (6H, s, CH_3);

^{13}C NMR (101 MHz, $CDCl_3$) δ (ppm): 140.1, 137.9, 137.1, 136.0, 131.7, 131.3, 128.3, 120.8, 21.2, 20.9,

HRMS (APPI, DCM) [M+H]+: m/z calc.: ($C_{15}H_{15}Br$): 274.0352, found: 274.0352.

1,2-bis(4-mesitylphenyl)ethyne (15)[7]

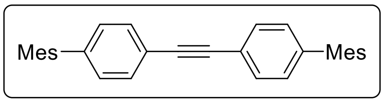

In a 50 mL Schlenk flask, compound **14** (300 mg, 1.09 mmol, 1.00 eq.), CuI (8.30 mg, 0.0436 mmol, 0.04 eq.) and $Pd(PPh_3)_2Cl_2$ (15.3 mg, 0.0218 mmol, 0.05 eq.) were dissolved in TEA (10 mL), degassed *via* sonication (2 × 3 min) and purged with argon. The reaction was started by addition of molecule **10** (240 mg, 1.09 mmol, 1.00 eq.) in solid state and one portion and stirred at 70°C for 22 h. The solvent was evaporated *in vacuo* and the reaction mixture was purified *via* flash column chromatography (SiO_2, hexanes/DCM, 9:1) to obtain the product as a colourless solid (199 mg, 0.480 mmol, 44 %). Additionally, starting material 14 could be recovered and reused in another batch (200 mg, 0.727 mmol, 66 %).

R_f = 0.28 (hexanes/DCM 9:1);

m.p. = 248 °C;

^{1}H NMR: (400 MHz, $CDCl_3$) δ (ppm): 7.60-7.57 (4H, m, ar-H), 7.15-7.12 (4H, m, ar-H), 6.94 (4H, s, ar-H), 2.32 (6H, s, CH_3), 2.01 (12H, s, CH_3);

^{13}C NMR: (101 MHz, $CDCl_3$) δ (ppm): 141.5, 138.5, 137.0, 136.0, 131.8, 129.6, 128.3, 121.7, 89.5, 21.2, 20.9;

HRMS (APPI, DCM) [M+H]+: m/z calc.: ($C_{32}H_{31}$): 415.2420, found: 415.2419.

Hexamesityl-HPB (16)[61]

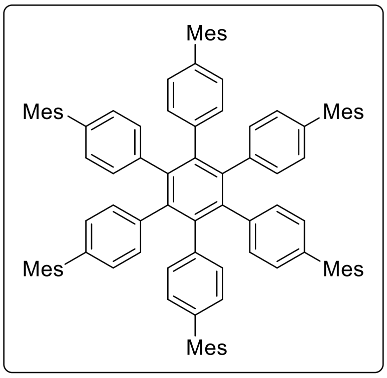

In a microwave pressure vial, cyclopentadienone **8** (207 mg, 0.241 mmol, 1.0 eq.) and acetylene **15** (100 mg, 0.241 mmol, 1.0 eq.) were dissolved in Ph_2O (0.844 mL, 903 mg, 5.31 mmol, 22 eq.) and degassed for 5 min. The reaction was carried out at 250° C for 18 h under microwave irradiation. After cooling to room temperature, the product was precipitated from the mixture with cold methanol and obtained as off-white solid (265 mg, 0.213 mmol, 88 %).

m.p. = decomposition >350 °C;

^{1}H NMR (400 MHz, $CDCl_3$) δ (ppm): 6.99-6.97 (12H, m, ar-H), 6.84 (12H, s, ar-H), 6.67-6.64 (4H, m, ar-H), 2.27 (18H, s, CH_3), 1.78 (36H, s, CH_3);

^{13}C NMR: (101 MHz, $CDCl_3$) δ (ppm): 140.6, 139.4, 139.2, 138.1, 136.4, 136.1, 132.1, 127.9, 127.7, 21.2, 20.6;

MS (MALDI-dctb): m/z Calc.: ($C_{96}H_{90}$): 1243.7076, found: 1243.7663.

Hexamesityl-HBC (HBC-Ref)[61]

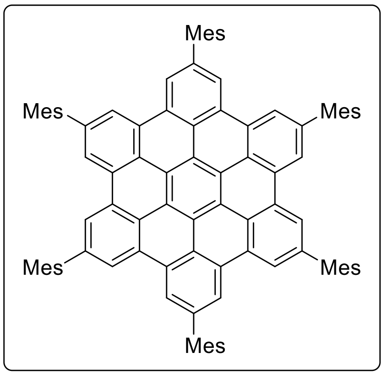

Under inert atmosphere, HPB **16** (60.0 mg, 0.0483 mmol, 1.0 eq.) and DDQ (77.0 mg, 0.338 mmol, 7.0 eq.) were dissolved in dry DCM (15 mL), cooled to 0 °C and degassed for 15 min. Then, TfOH (60.0 µL, 101 mg, 0.676 mmol, 14 eq.) was added and the reaction was stirred for 3 h while gradually warming to room temperature. It was ended *via* the addition of TEA (2.5 mL). The solvent was removed *in vacuo* and the residue was purified by plug chromatography (SiO_2, DCM, 100 %) to yield the product is orange powder (49.3 mg, 0.040 mmol, 82 %).

m.p. = decomposition >340 °C;

^{1}H NMR (400 MHz, $CDCl_3$) δ (ppm): 9.01 (12H, s, ar-H), 7.07 (12H, s, ar-H), 2.41 (18H, s, CH_3), 2.23 (36H, s, CH_3).

^{13}C NMR: (101 MHz, $CDCl_3$) δ (ppm): 140.1, 139.2, 137.3, 136.3, 131.1, 128.6, 124.7, 123.7, 121.4, 21.5, 21.3;

HRMS (MALDI-dctb): m/z Calc.: ($C_{96}H_{78}$): 1230.6098, found: 1230.6121.

Characterization

NMR spectra

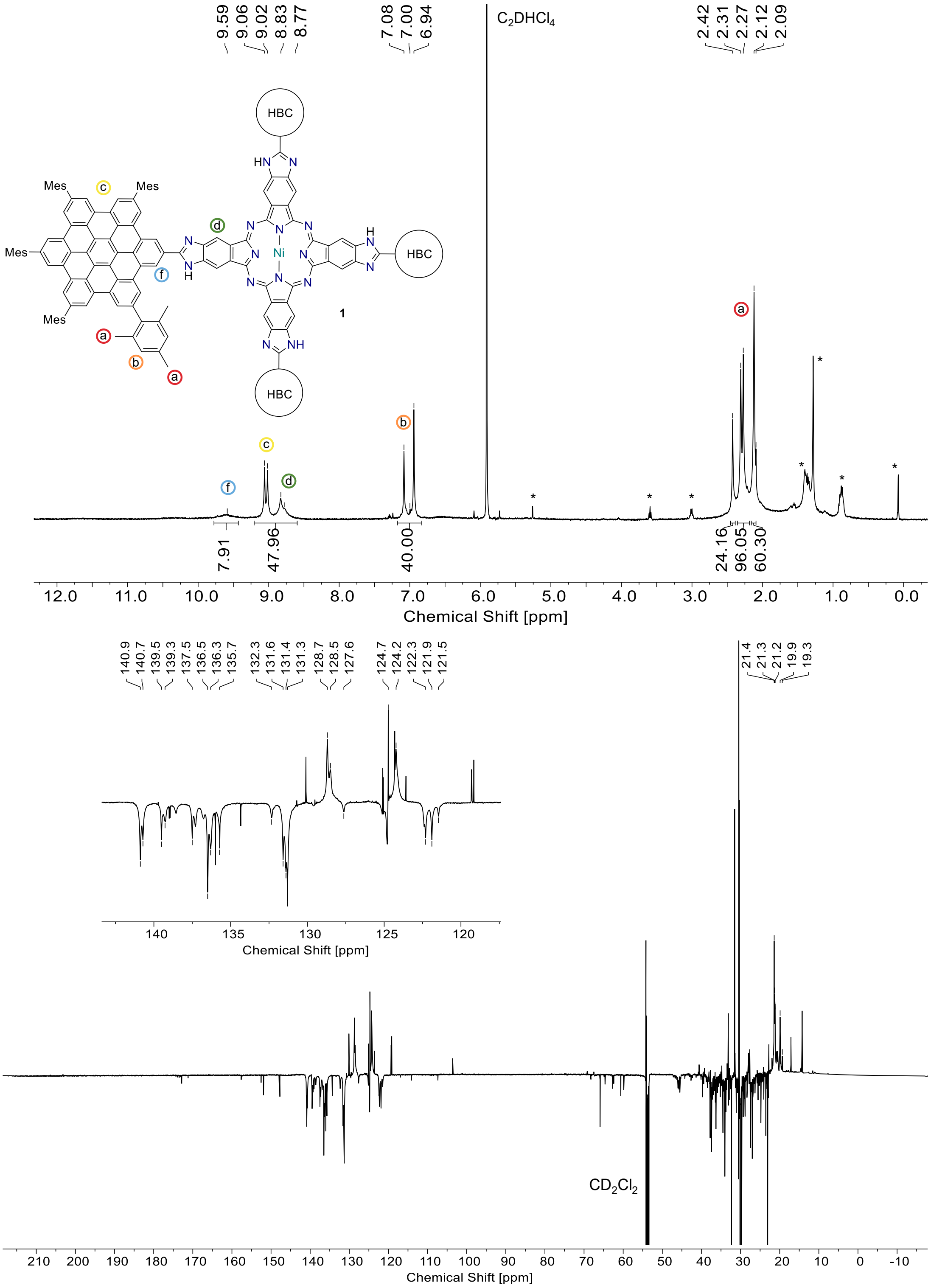

*Figure S1-1: ¹H NMR (500 MHz, 130°C, $C_2D_2Cl_4$) of **01**, solvent signal is labelled, stars indicate small impurity residues of DCM, THF, water, hexanes, grease and silicone grease; S1-2: DEPTq135 NMR (151 MHz, rt, CD_2Cl_2) of **01**, CH/CH_3 up, CH_2/Cquart. down, located between 25-40 ppm are impurities (grease, hexanes) and resulting artefacts only visible in the measurement at 151 MHz (compare overlay in Figure S1-4);*

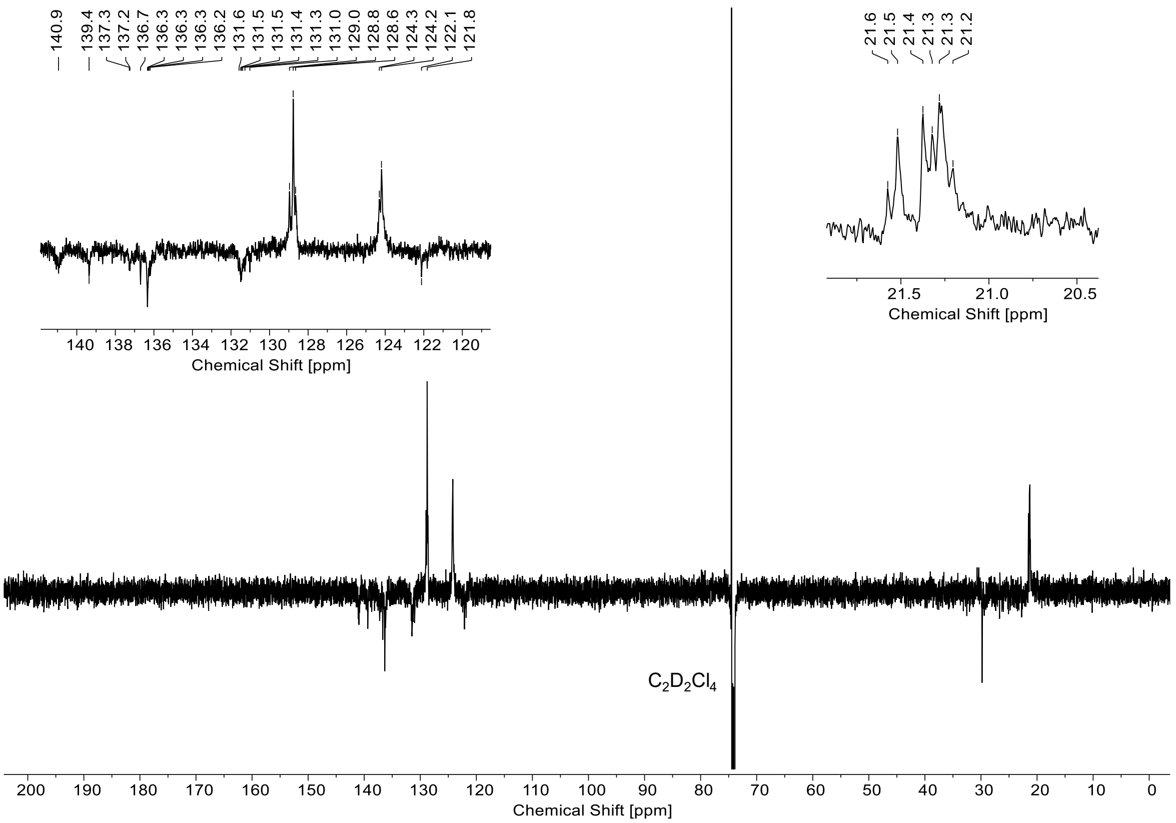

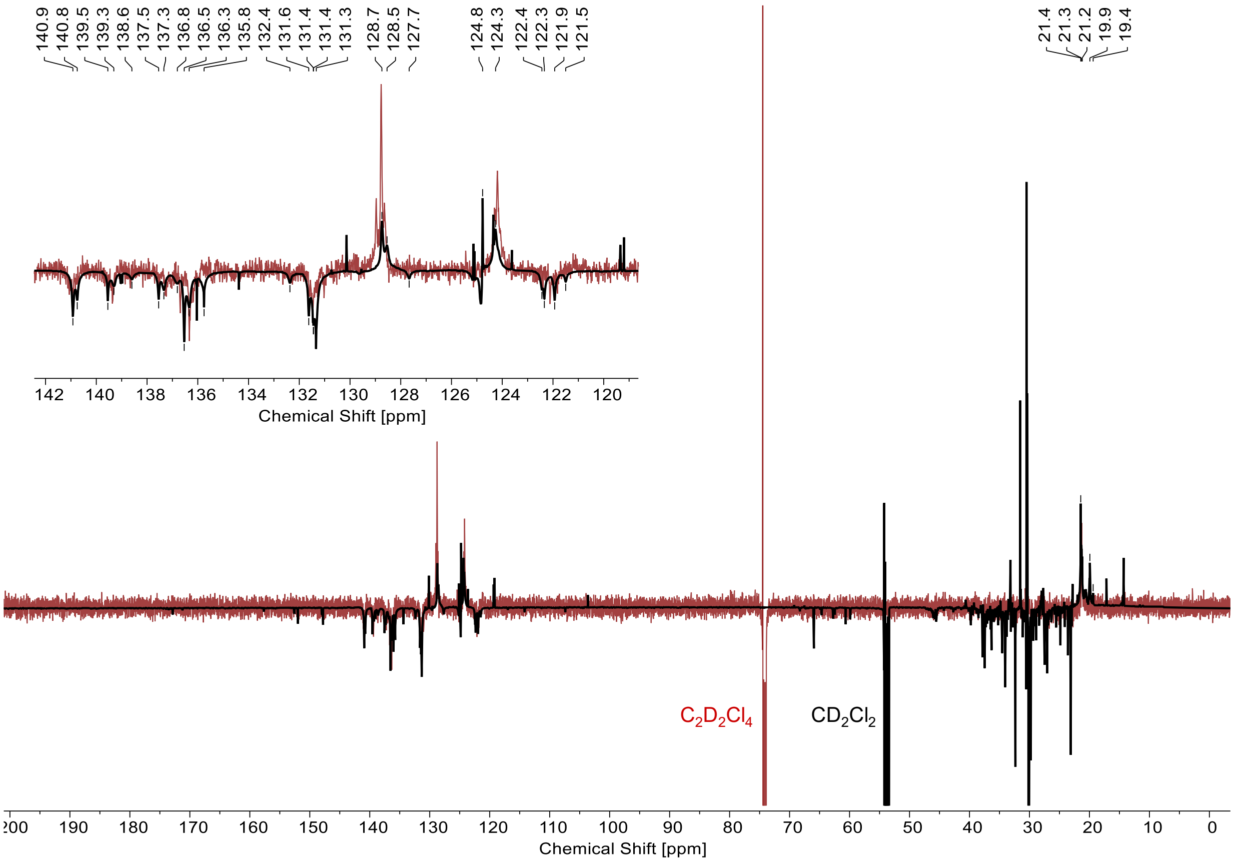

*S1-3: DEPTq135 NMR (126 MHz, 110°C, $C_2D_2Cl_4$ [sic!]) of **01**, CH/CH_3 up, CH_2/Cquart. down, impurities at ≈30 ppm are less intense here and do not result in artefacts, however, the resolution of compound signals is less distinct than in the measurement of Figure S1-2; S1-4: overlay of the two DEPTq135 NMR spectra of **01** in different solvents.*

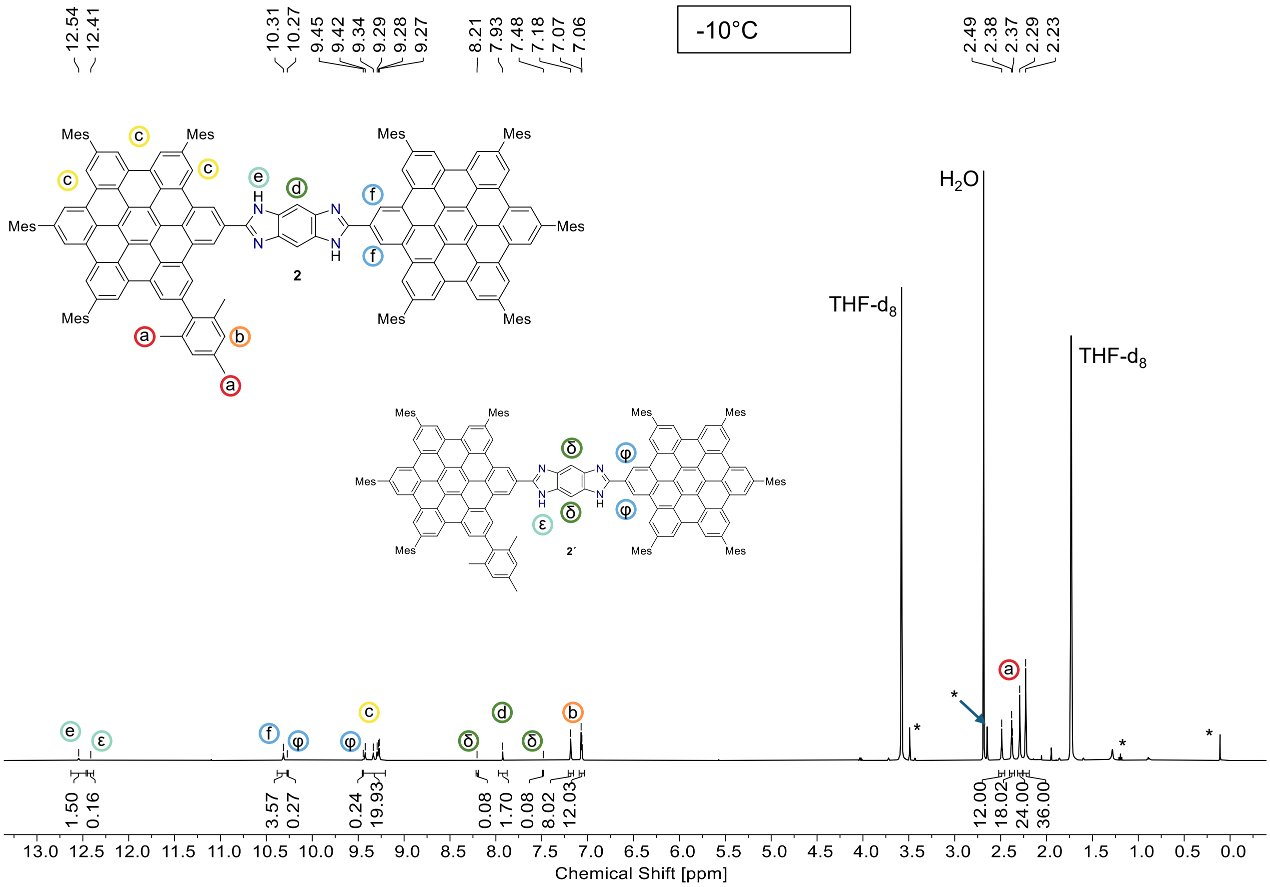
-10°C
12.54
12.41
10.31
10.27
9.45
9.42
9.34
9.29
9.28
9.27
8.21
7.93
7.48
7.18
7.07
7.06
2.49
2.38
2.37
2.29
2.23
Mes
2
2´
H_2O
THF-d_8
THF-d_8
*
1.50
0.16
3.57
0.27
0.24
19.93
0.08
1.70
0.08
8.02
12.03
12.00
18.02
24.00
36.00
13.0 12.5 12.0 11.5 11.0 10.5 10.0 9.5 9.0 8.5 8.0 7.5 7.0 6.5 6.0 5.5 5.0 4.5 4.0 3.5 3.0 2.5 2.0 1.5 1.0 0.5 0.0
Chemical Shift [ppm]

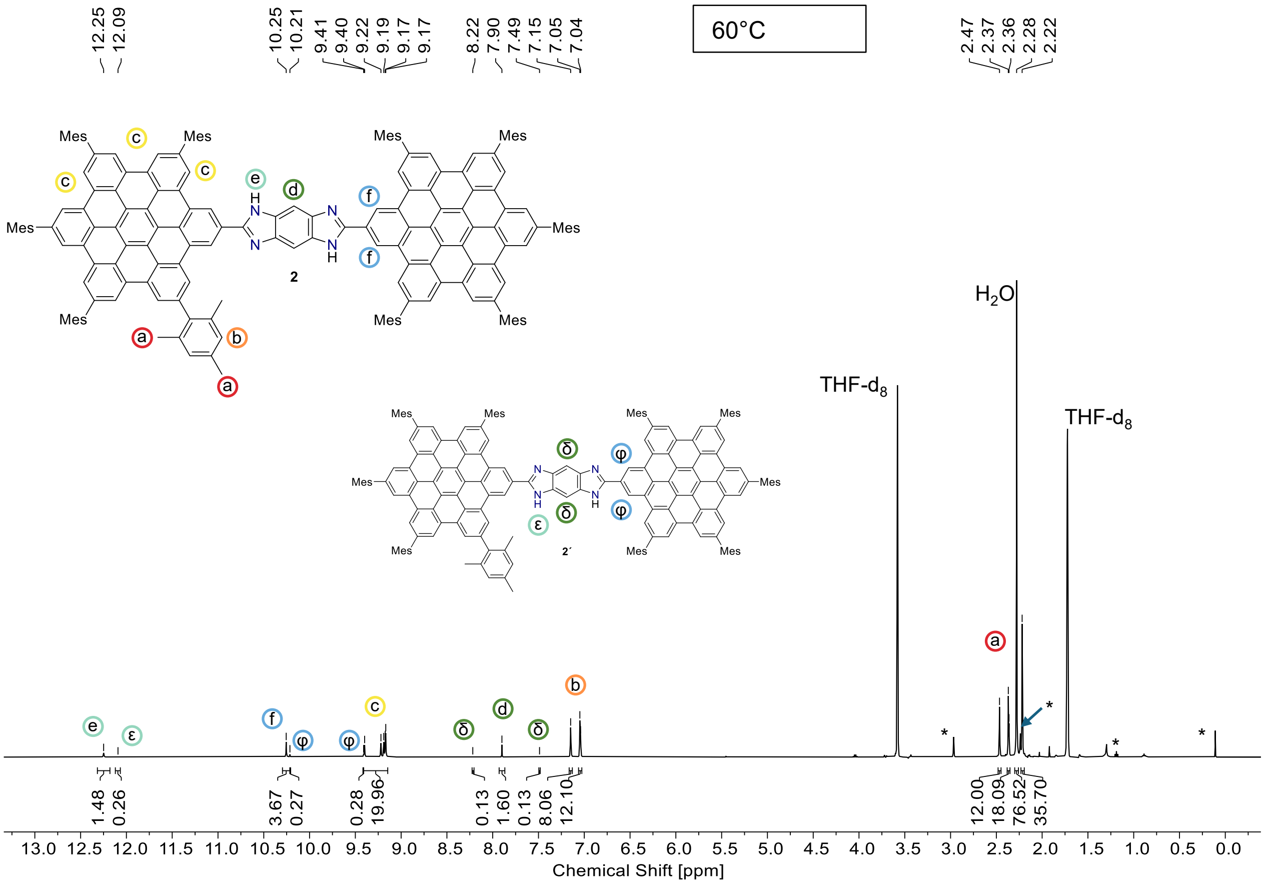
60°C
12.25
12.09
10.25
10.21
9.41
9.40
9.22
9.19
9.17
9.17
8.22
7.90
7.49
7.15
7.05
7.04
2.47
2.37
2.36
2.28
2.22
Mes
2
2´
H_2O
THF-d_8
THF-d_8
*
1.48
0.26
3.67
0.27
0.28
19.96
0.13
1.60
0.13
8.06
12.10
12.00
18.09
76.52
35.70
13.0 12.5 12.0 11.5 11.0 10.5 10.0 9.5 9.0 8.5 8.0 7.5 7.0 6.5 6.0 5.5 5.0 4.5 4.0 3.5 3.0 2.5 2.0 1.5 1.0 0.5 0.0
Chemical Shift [ppm]

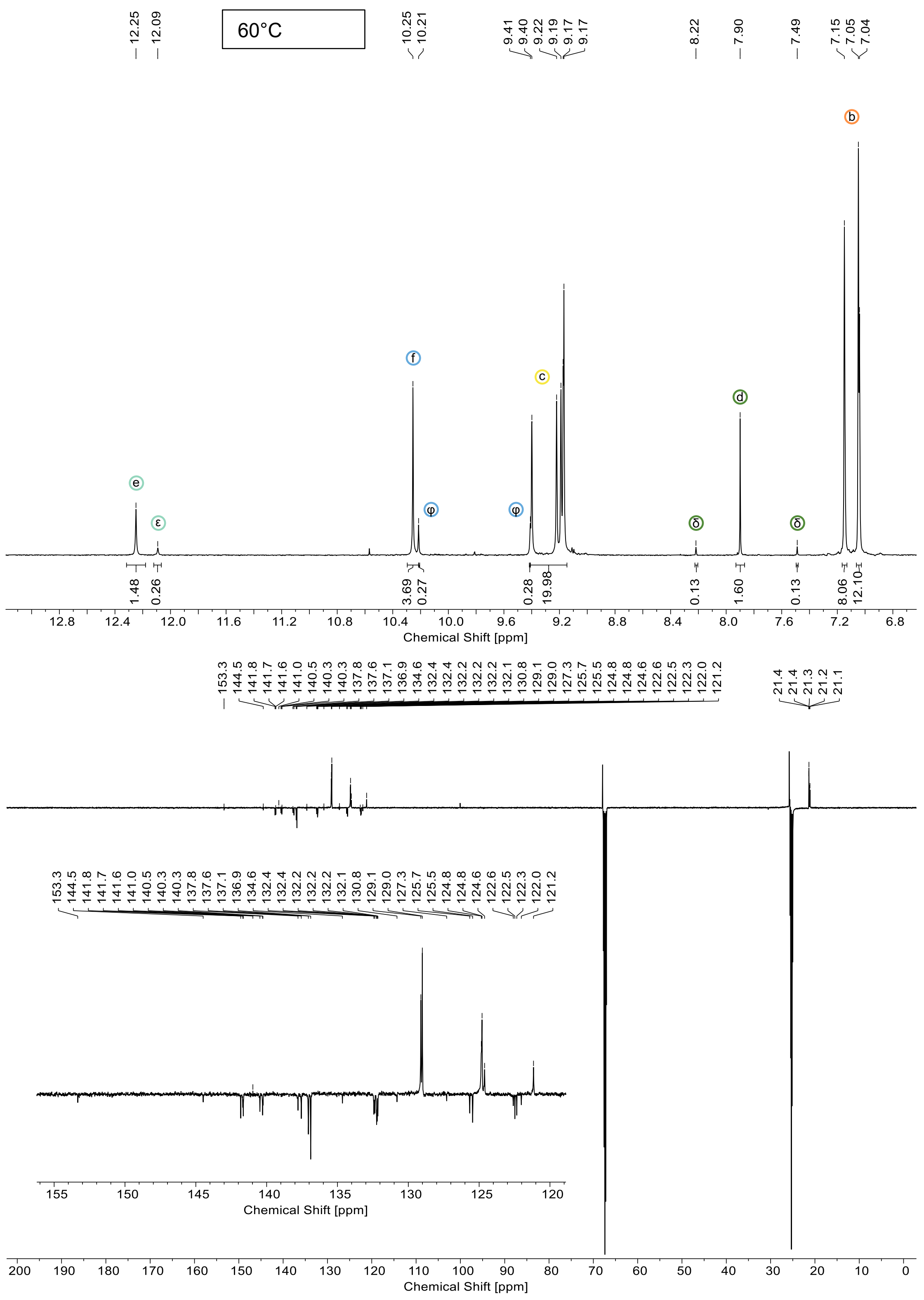

*Figure S2-1: ^{1}H NMR (500 MHz, -10°C, THF-d_8) of **02**, solvent signals and water residue are labelled, stars indicate small impurity residues of methanol, pentane, grease and silicone grease; S2-2: ^{1}H NMR (500 MHz, 60°C, THF-d_8) of **02**, analogous labelling, note that the water residue overlaps with the signal at 2.28 ppm, thus influencing the integration of this signal; S2-3: zoom into aromatic region of the ^{1}H NMR spectrum (500 MHz, 60°C, THF-d_8) of **02**; S2-4: DEPTq135 NMR (126 MHz, 60°C, THF-d_8) of **02**, CH/CH$_3$ up, CH$_2$/Cquart. down.*

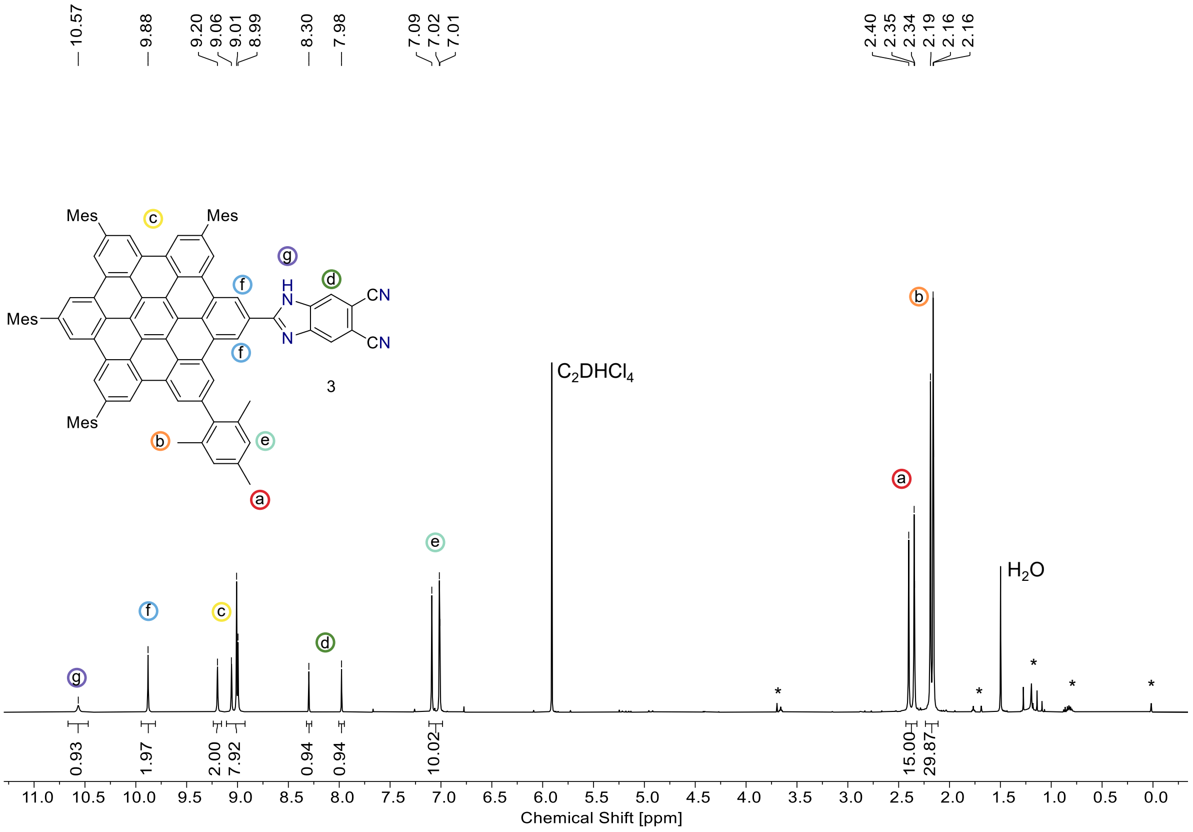
10.57
9.88
9.20
9.06
9.01
8.99
8.30
7.98
7.09
7.02
7.01
2.40
2.35
2.34
2.19
2.16
2.16
Mes
Mes
Mes
Mes
CN
CN
3
C_2DHCl_4
H_2O
0.93
1.97
2.00
7.92
0.94
0.94
10.02
15.00
29.87
Chemical Shift [ppm]

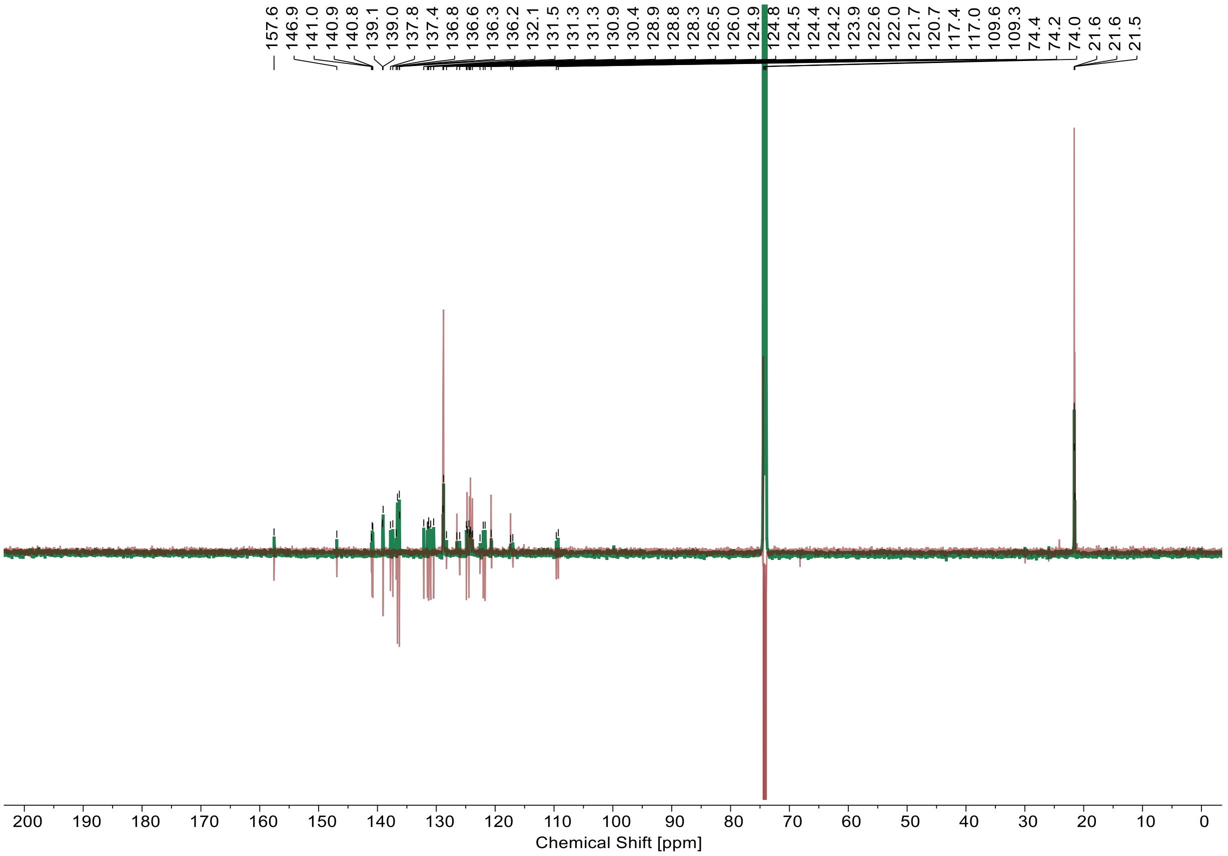
157.6
146.9
141.0
140.9
140.8
139.1
139.0
137.8
137.4
136.8
136.6
136.3
136.2
132.1
131.5
131.3
130.9
130.4
128.9
128.8
128.3
126.5
126.0
124.9
124.8
124.5
124.4
124.2
123.9
122.6
122.0
121.7
120.7
117.4
117.0
109.6
109.3
74.4
74.2
74.0
21.6
21.6
21.5
Chemical Shift [ppm]

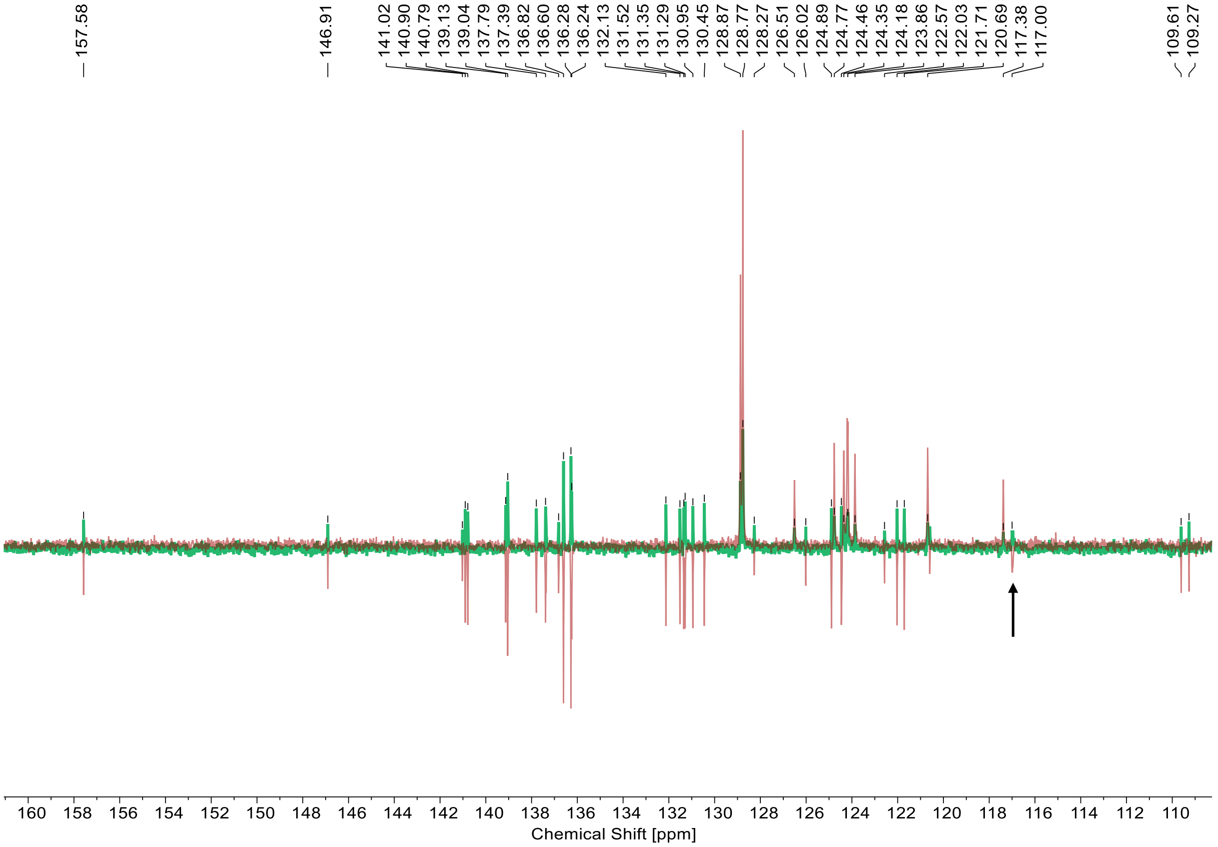
157.58
146.91
141.02
140.90
140.79
139.13
139.04
137.79
137.39
136.82
136.60
136.28
136.24
132.13
131.52
131.35
131.29
130.95
130.45
128.87
128.77
128.27
126.51
126.02
124.89
124.77
124.46
124.35
124.18
123.86
122.57
122.03
121.71
120.69
117.38
117.00
109.61
109.27
Chemical Shift [ppm]

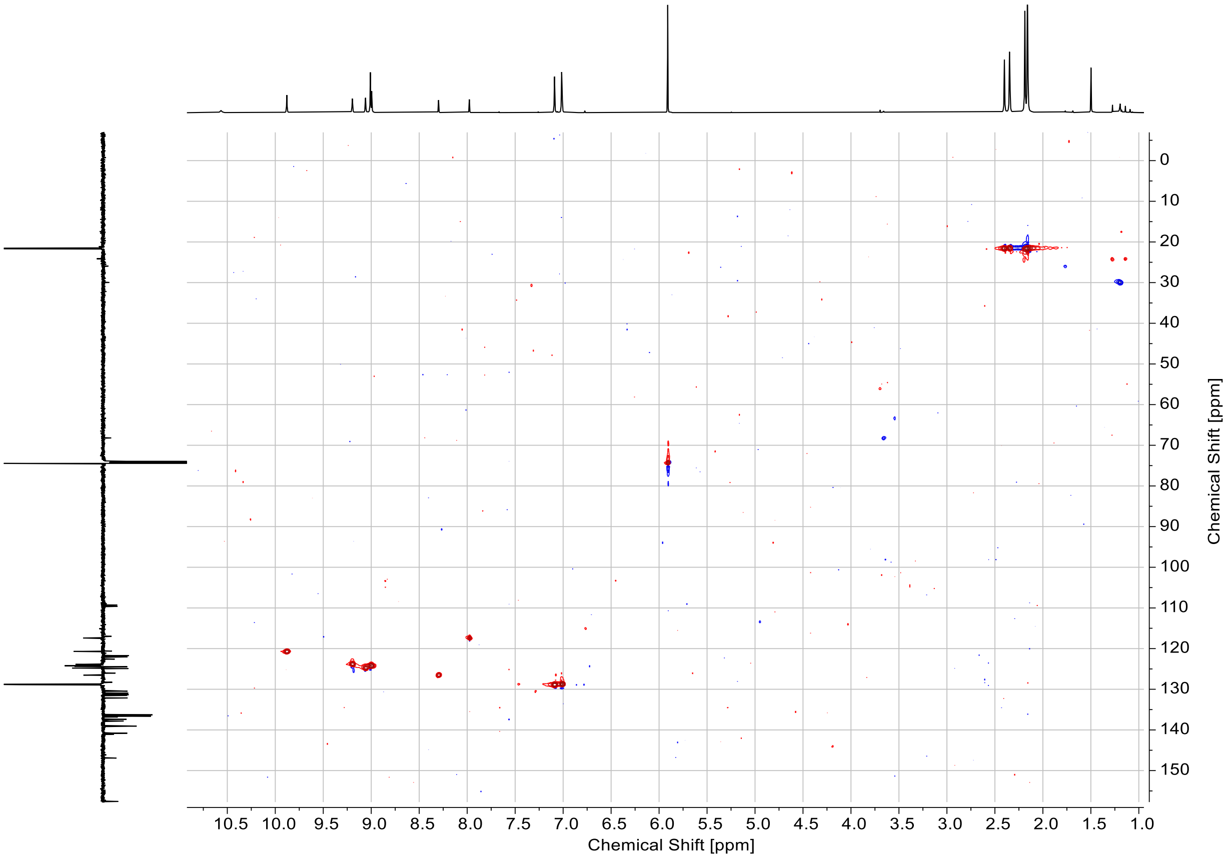
Chemical Shift [ppm]
Chemical Shift [ppm]

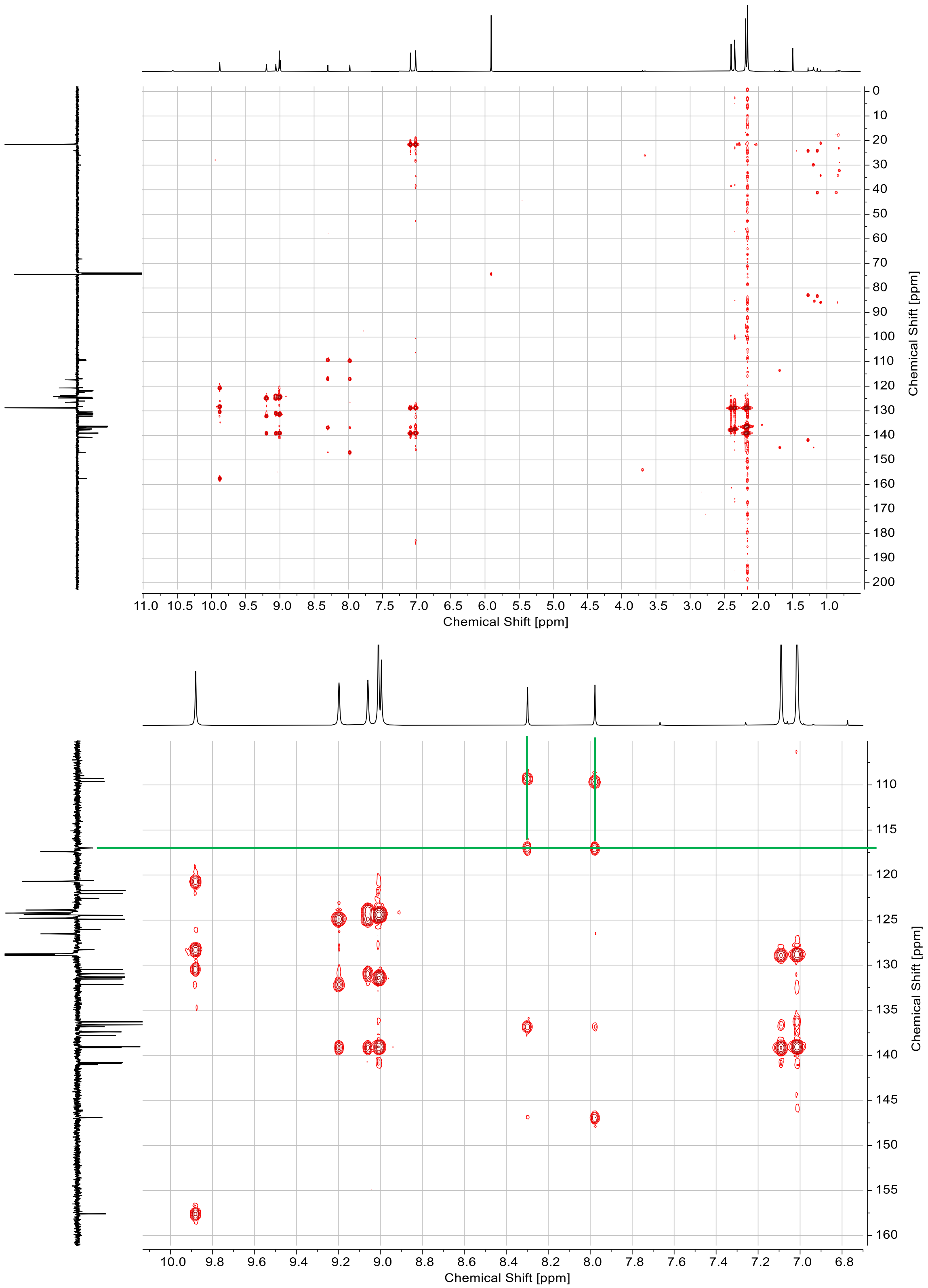

*Figure S3-1: ^{1}H NMR (500 MHz, 45°C, $C_2D_2Cl_4$) of **03**, solvent and water are labelled, stars indicate small impurity residues of THF, hexanes, grease and silicone grease; S3-2: layered ^{13}C NMR spectrum (green, front) and DEPTq135 NMR spectrum (red, back) (126 MHz, 45°C, $C_2D_2Cl_4$) of **03**, CH/CH_3 up, CH_2/Cquart. down; S3-3: zoom into the aromatic region of the DEPTq135 spectrum, highlighted: nitrile carbon atom signal; S3-4: HSQC spectrum; S3-5: HMBC spectrum; S3-6: zoom into the aromatic region of the HMBC spectrum with the nitrile-C atom to benzimidazole-proton cross peaks highlighted.*

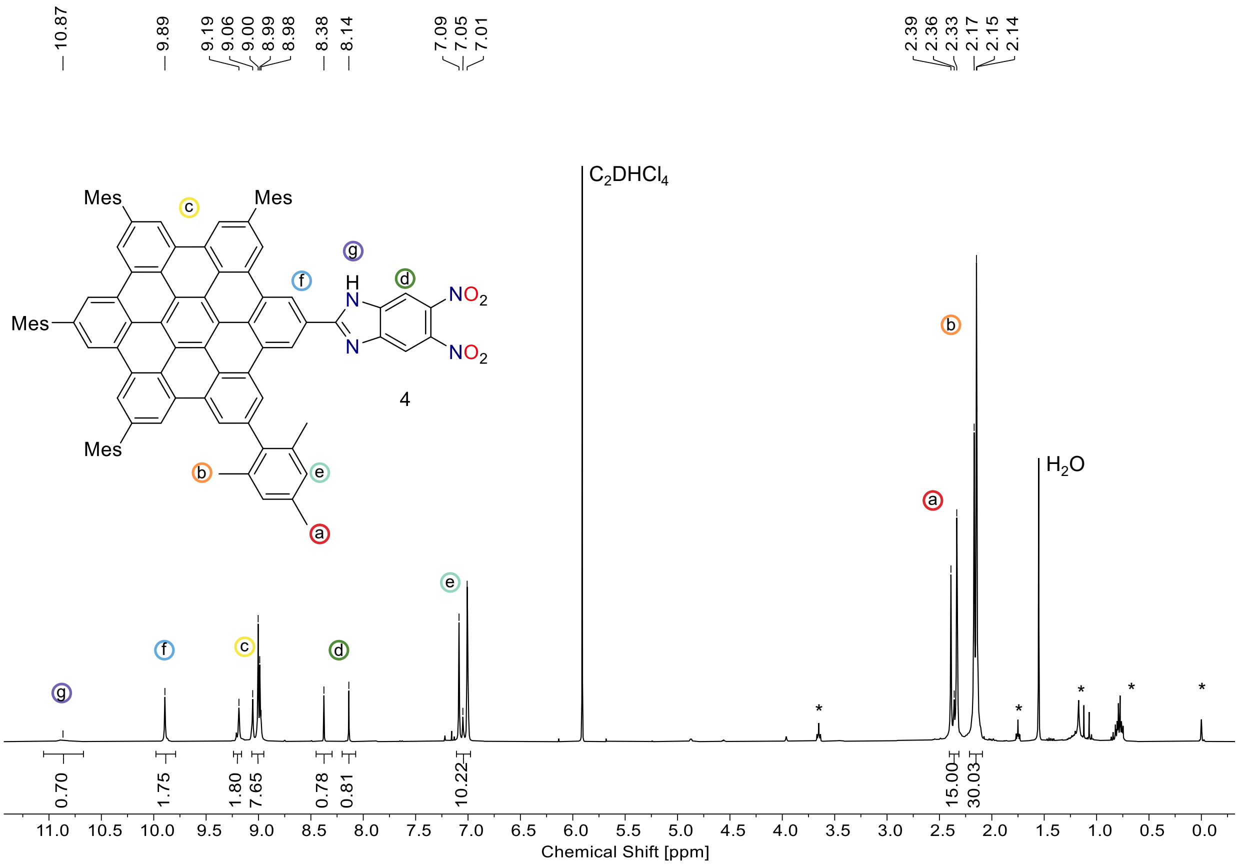
10.87
9.89
9.19
9.06
9.00
8.99
8.98
8.38
8.14
7.09
7.05
7.01
2.39
2.36
2.33
2.17
2.15
2.14
Mes
Mes
Mes
Mes
H
N
N
NO2
NO2
4
a
b
c
d
e
f
g
C2DHCl4
H2O
*
0.70
1.75
1.80
7.65
0.78
0.81
10.22
15.00
30.03
11.0 10.5 10.0 9.5 9.0 8.5 8.0 7.5 7.0 6.5 6.0 5.5 5.0 4.5 4.0 3.5 3.0 2.5 2.0 1.5 1.0 0.5 0.0
Chemical Shift [ppm]

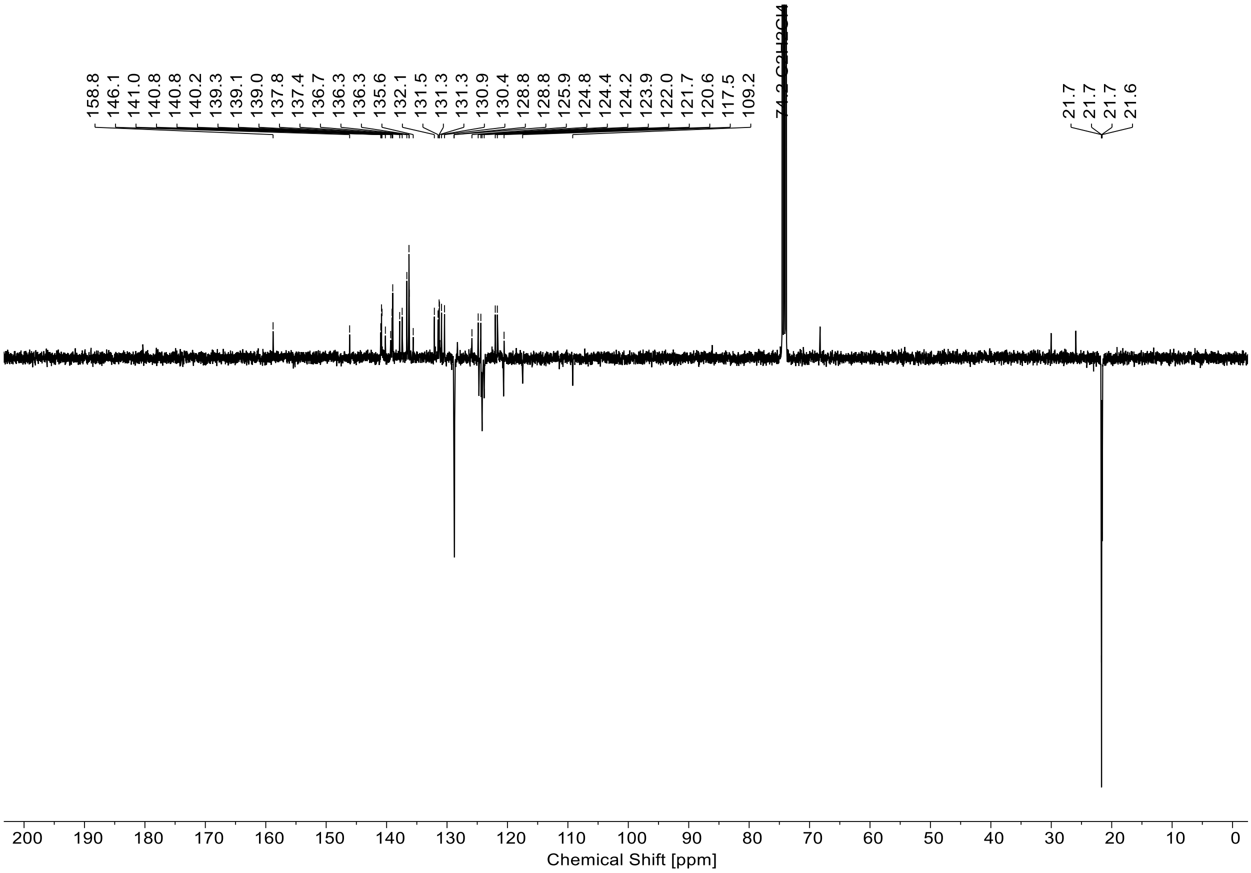
158.8
146.1
141.0
140.8
140.8
140.2
139.3
139.1
139.0
137.8
137.4
136.7
136.3
136.3
135.6
132.1
131.5
131.3
131.3
130.9
130.4
128.8
128.8
125.9
124.8
124.4
124.2
123.9
122.0
121.7
120.6
117.5
109.2
21.7
21.7
21.7
21.6
200 190 180 170 160 150 140 130 120 110 100 90 80 70 60 50 40 30 20 10 0
Chemical Shift [ppm]

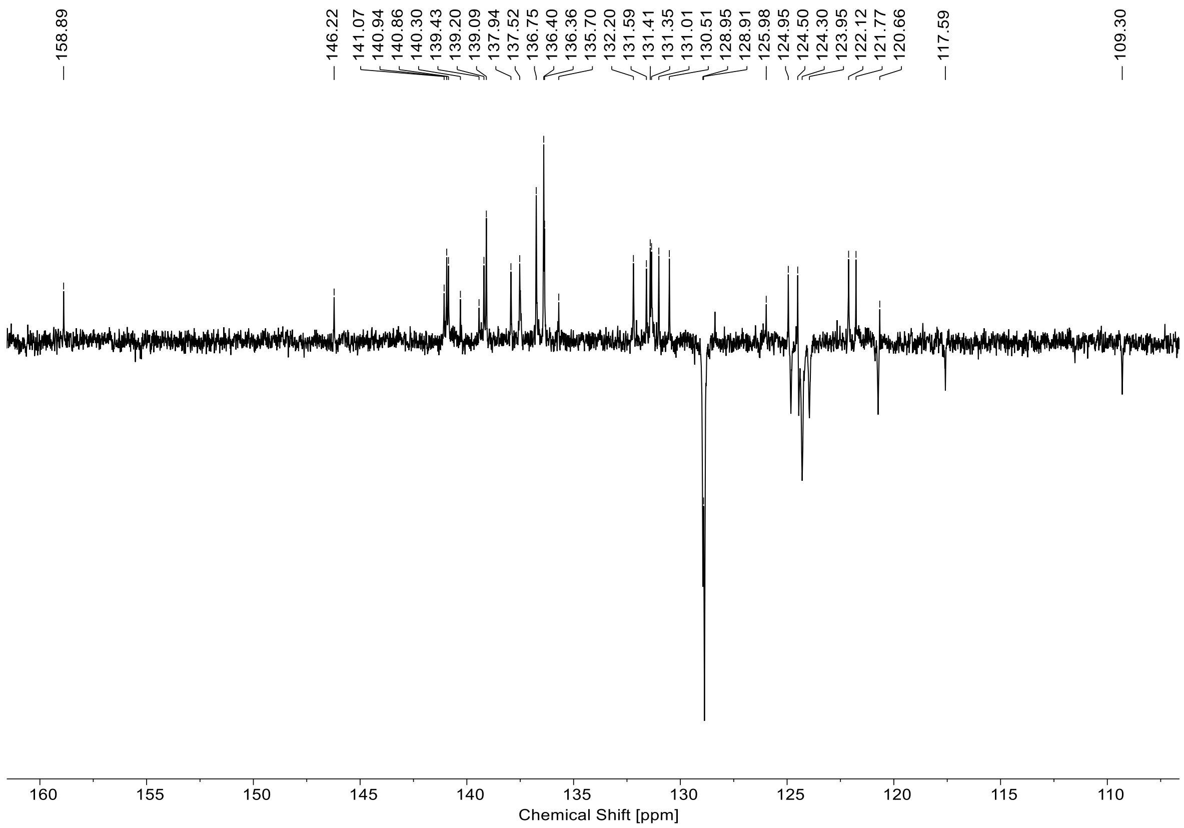

*Figure S4-1: ^{1}H NMR (400 MHz, rt, $C_2D_2Cl_4$) of **04**, solvent and water are labelled, stars indicate small impurity residues of THF, hexanes, grease and silicone grease; S4-2: inverted DEPTq135 NMR (101 MHz, rt, $C_2D_2Cl_4$) of **04**, CH/CH_3 down, CH_2/Cquart. up; S4-3: zoom into the aromatic region of the DEPTq135 spectrum.*

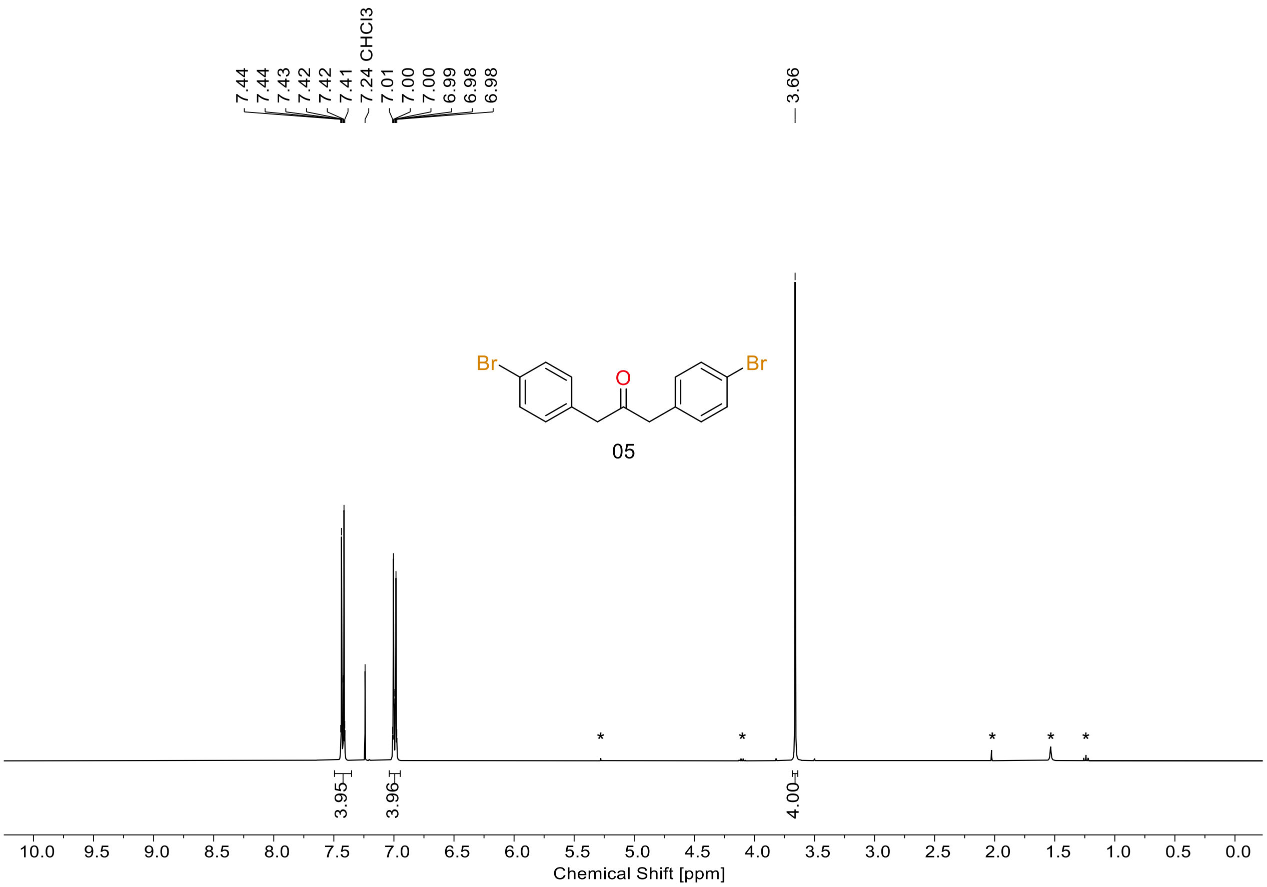

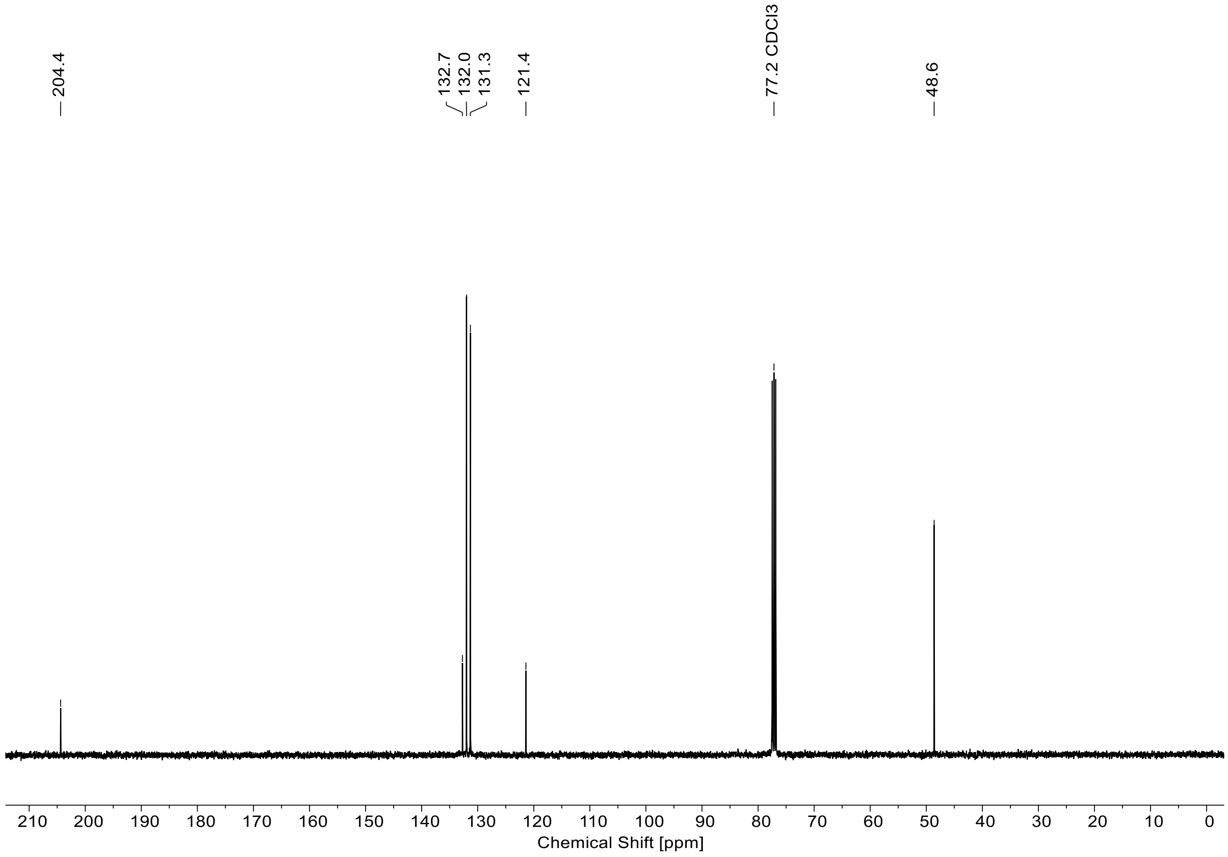

*Figure S5 Top: ^{1}H NMR (400 MHz, rt, $CDCl_3$) of **05**, stars indicate tiniest residues of DCM; water and ethyl acetate; bottom: ^{13}C NMR (101 MHz, rt, $CDCl_3$) of **05**.*

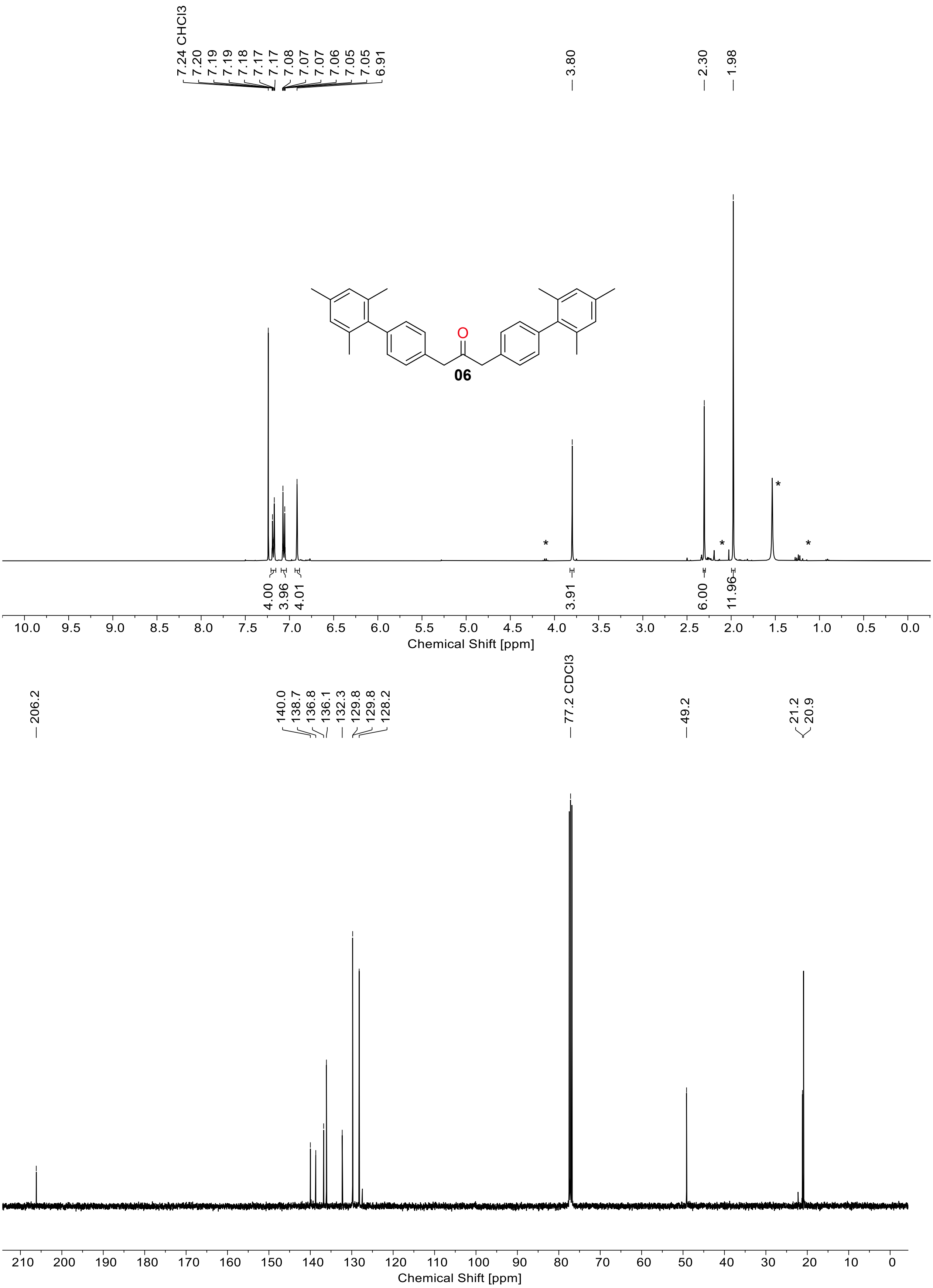

*Figure S6 Top: ^{1}H NMR (400 MHz, rt, CDCl$_3$) of **06**, stars indicate tiniest residues of water grease and ethyl acetate; bottom: ^{13}C NMR (101 MHz, rt, CDCl$_3$) of **06**.*

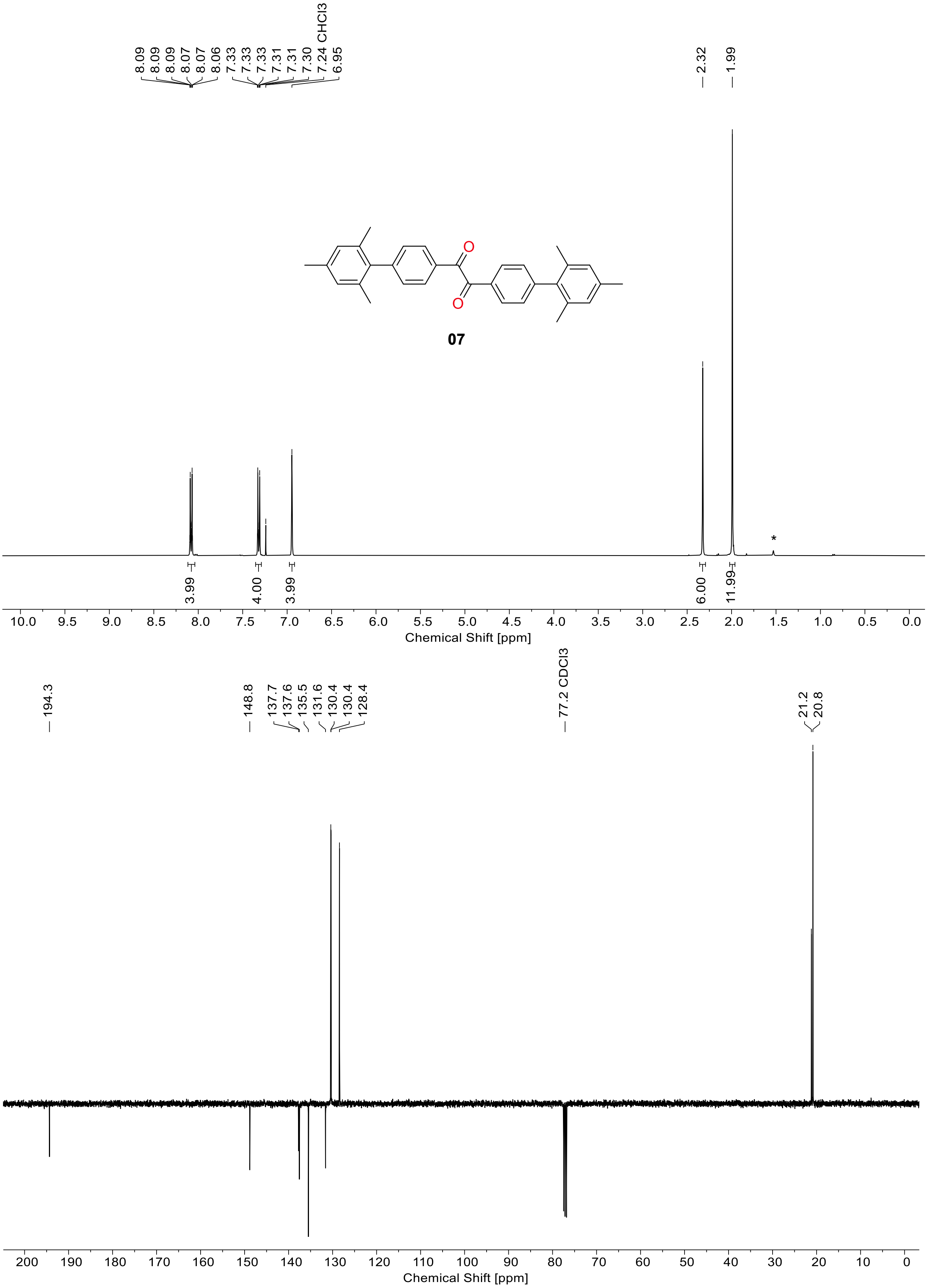

Figure S7 Top: ¹H NMR (400 MHz, rt, $CDCl_3$) of ***07****, star indicates a tiny residue of water; bottom: DEPTq135 NMR (101 MHz, rt, $CDCl_3$) of* ***07****, CH/CH_3 up, CH_2/Cquart. down.*

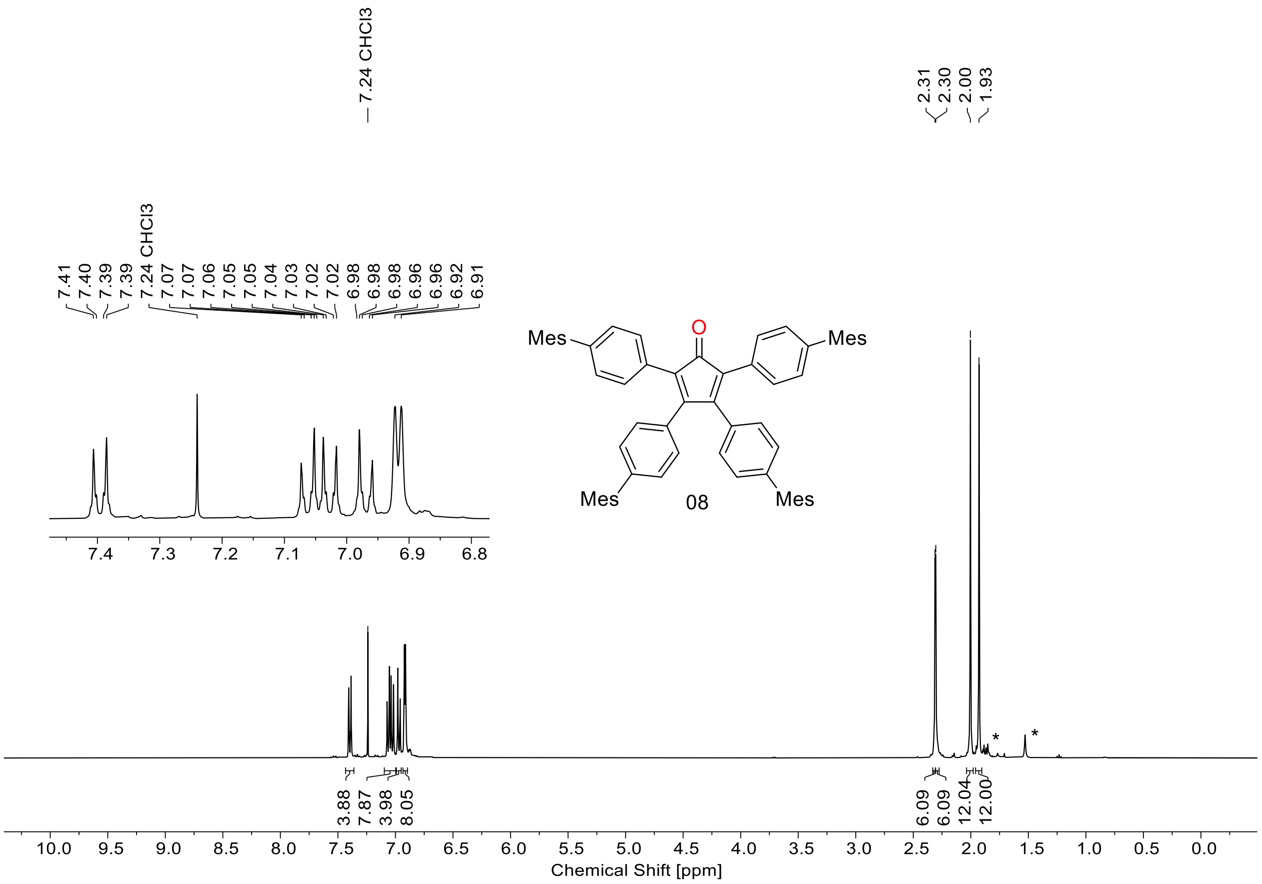

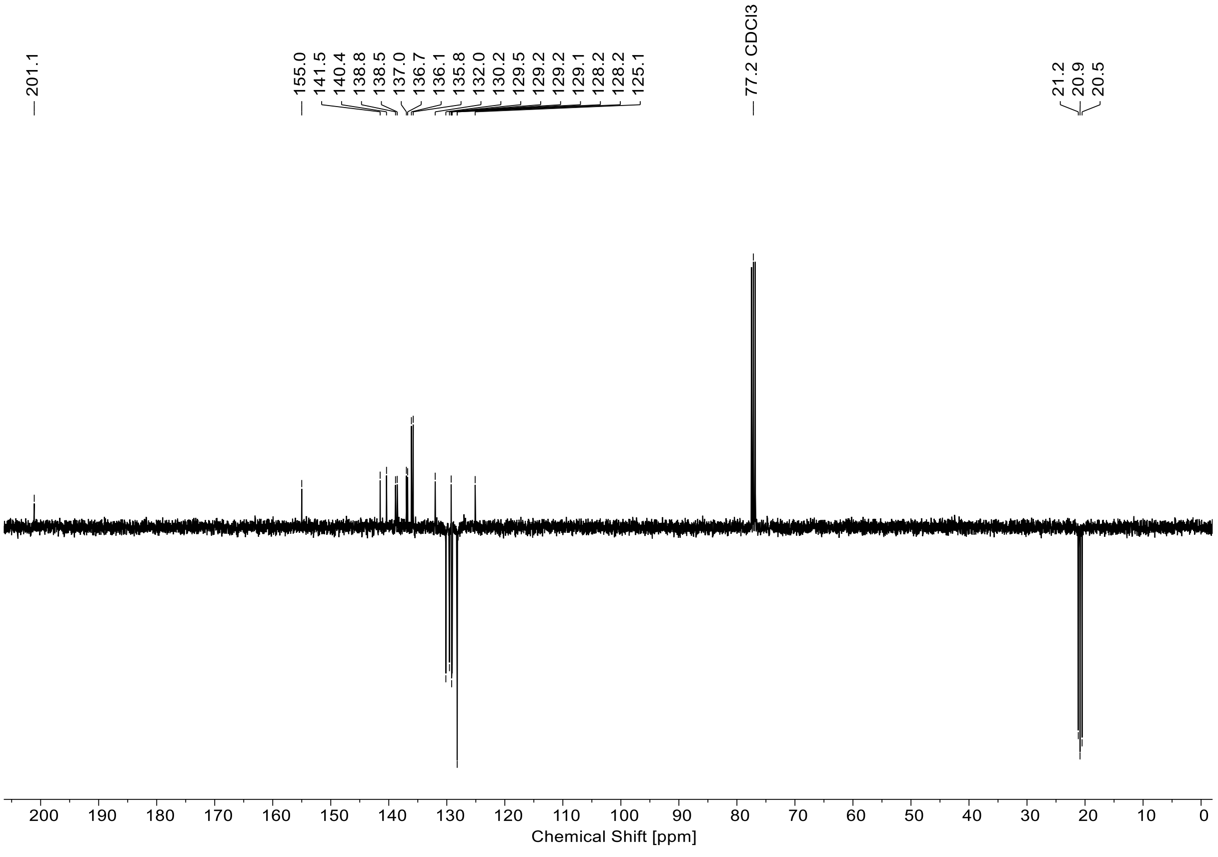

*Figure S8 Top ^{1}H NMR (400 MHz, rt, $CDCl_3$) of **08**, stars indicate tiny residues of grease and water; bottom: DEPTq135 NMR (101 MHz, rt, $CDCl_3$) of **08**, inverted CH/CH_3 down, CH_2/Cquart. up.*

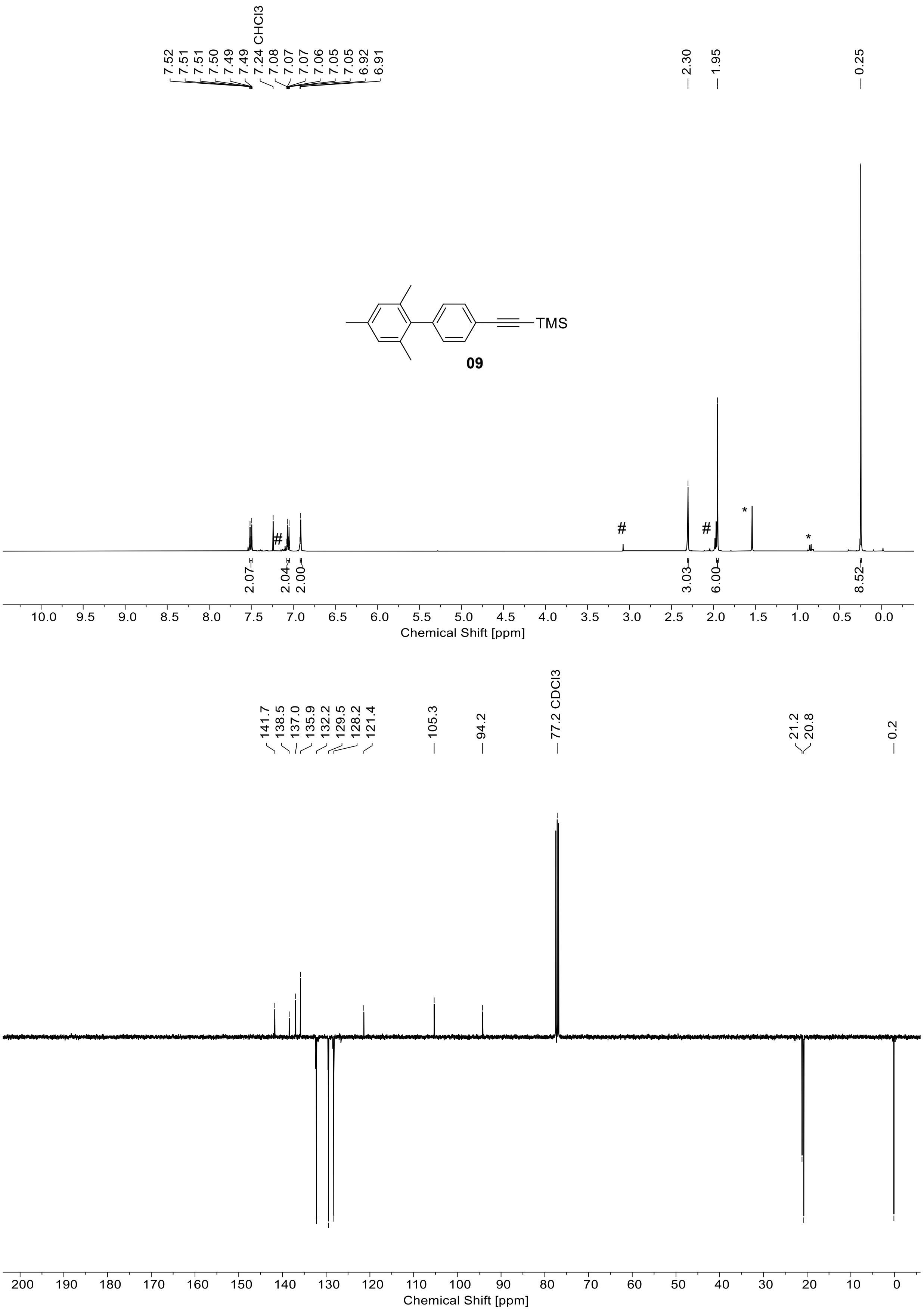

*Figure S9 Top: ^{1}H NMR (400 MHz, rt, $CDCl_3$) of **09**, stars indicate tiny residues of water and grease, # shows smalls amounts of already deprotected molecule **10**; bottom: DEPTq135 NMR (101 MHz, rt, $CDCl_3$) of **09**, inverted CH/CH_3 down, CH_2/Cquart. up.*

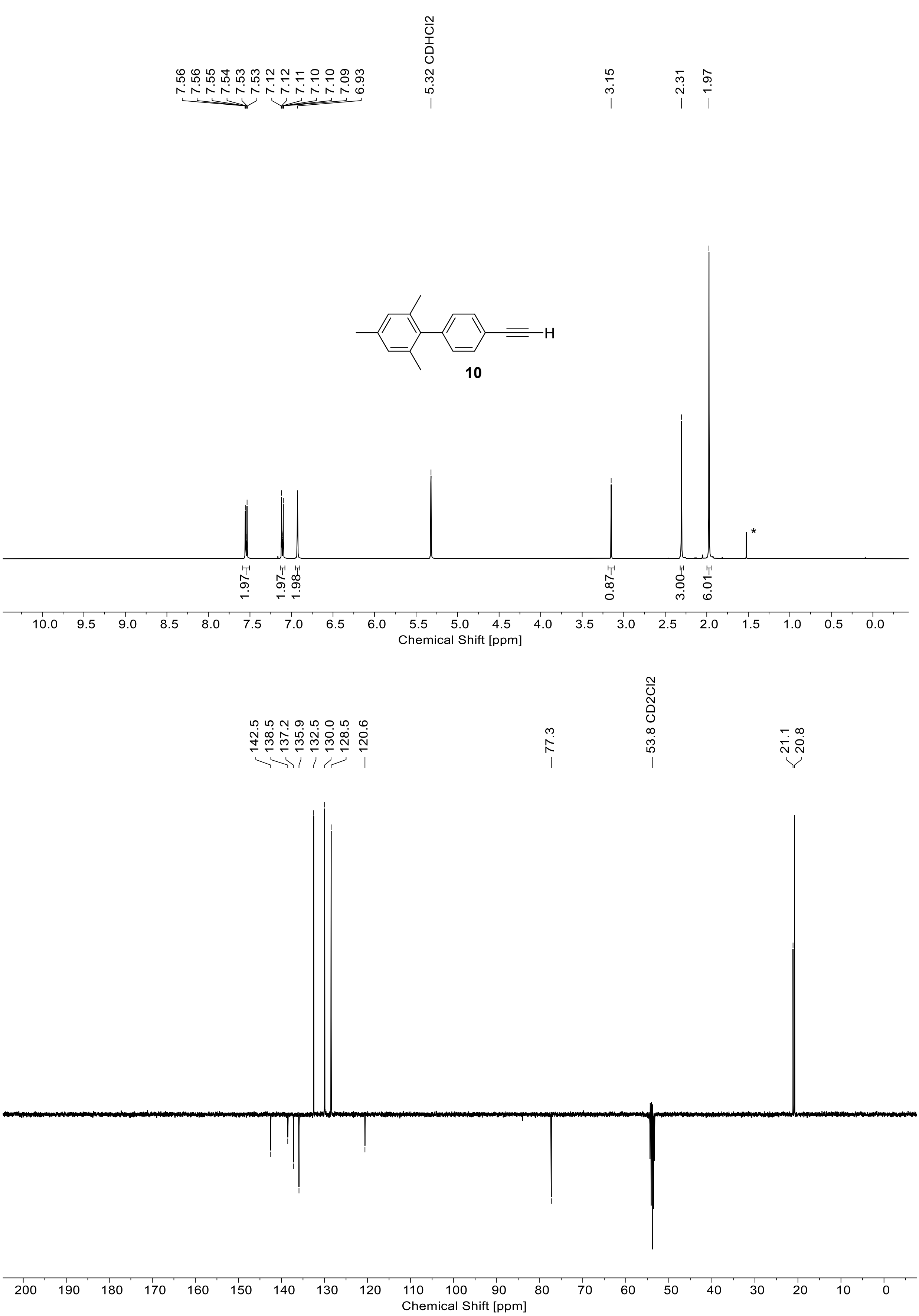

*Figure S10 Top: ^{1}H NMR (400 MHz, rt, CD_2Cl_2) of **10**, the star indicates a residue of water; bottom: DEPTq135 NMR (101 MHz, rt, CD_2Cl_2) of **10**, CH/CH_3 up, CH_2/Cquart. down.*

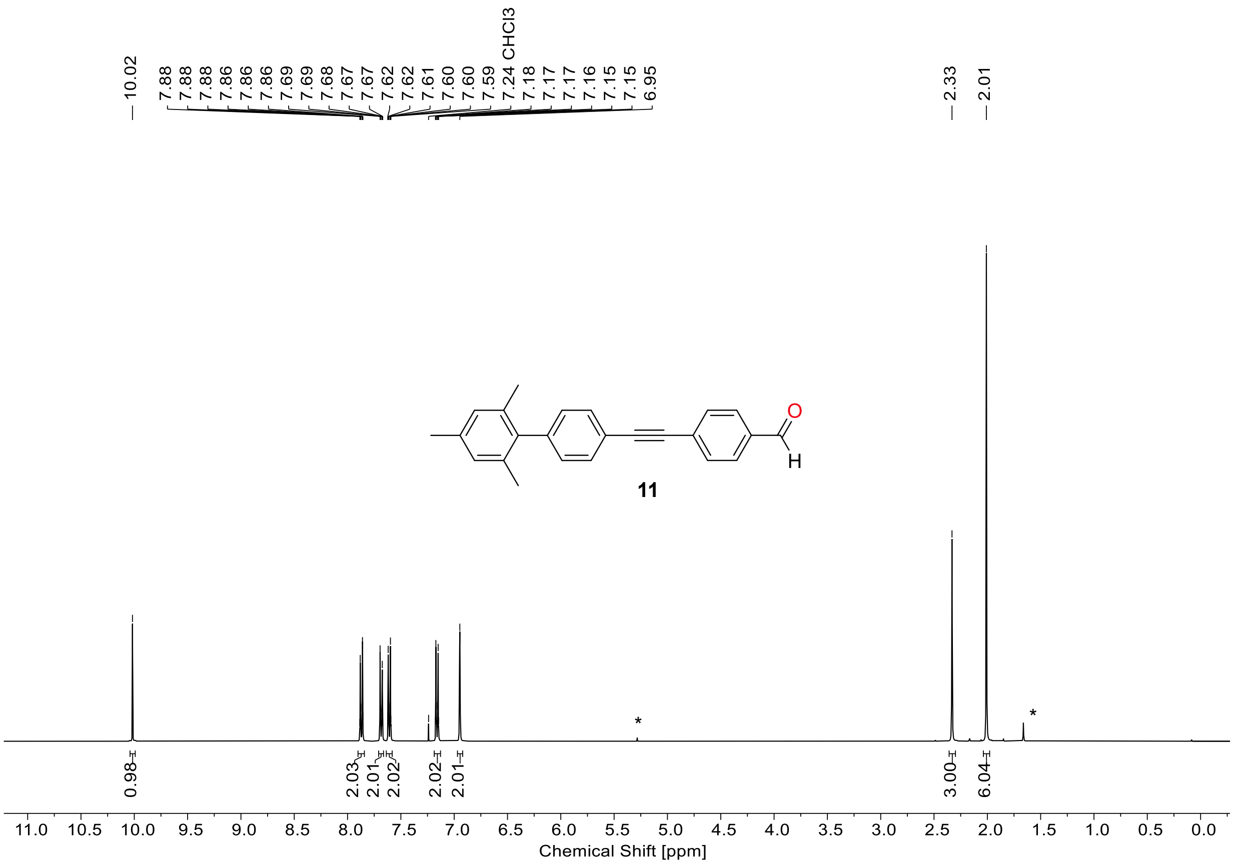

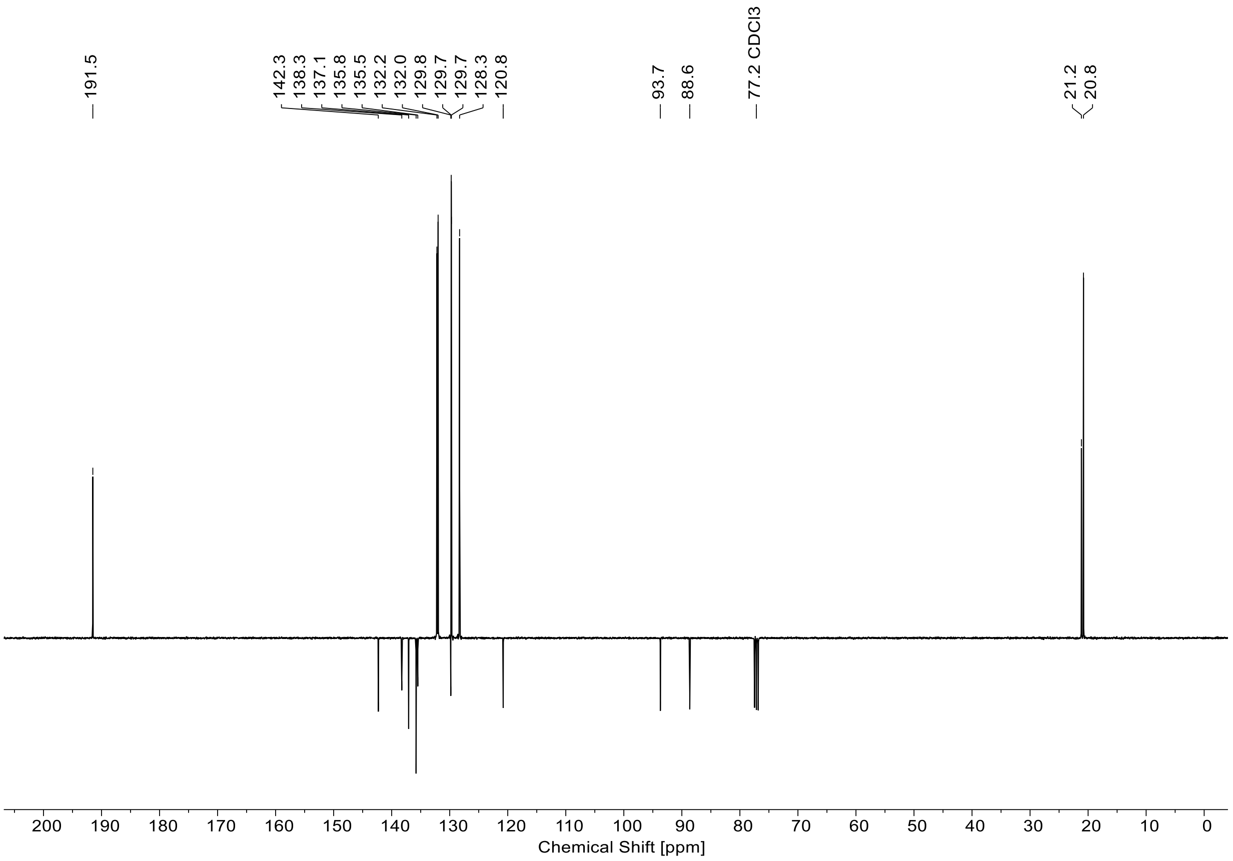

*Figure S11 Top: ^{1}H NMR (400 MHz, rt, $CDCl_3$) of **11**, stars indicate tiniest residues of DCM and water; bottom: DEPTq135 NMR (101 MHz, rt, $CDCl_3$) of **11**, CH/CH_3 up, CH_2/Cquart. down.*

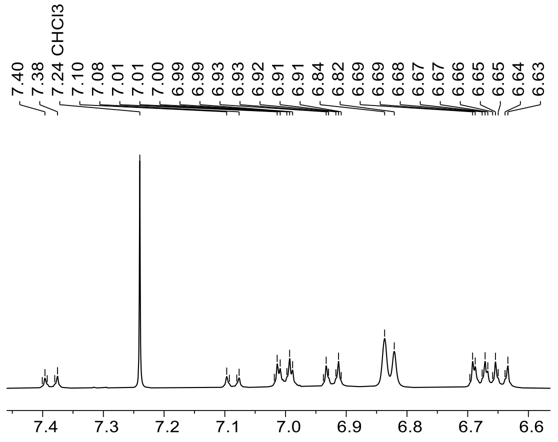

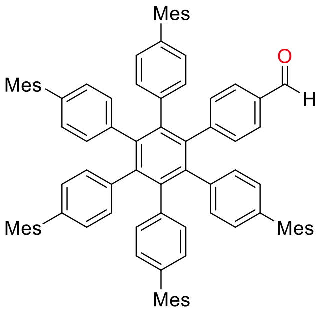

12

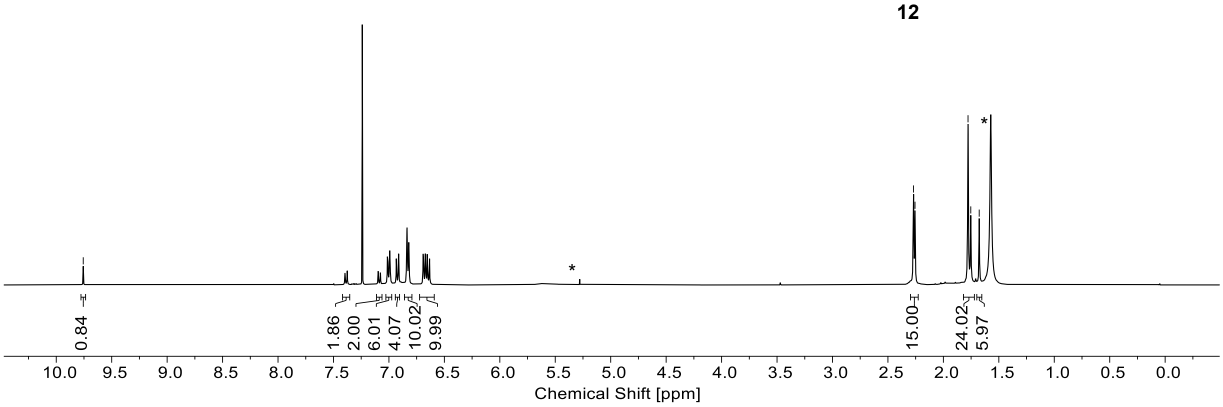

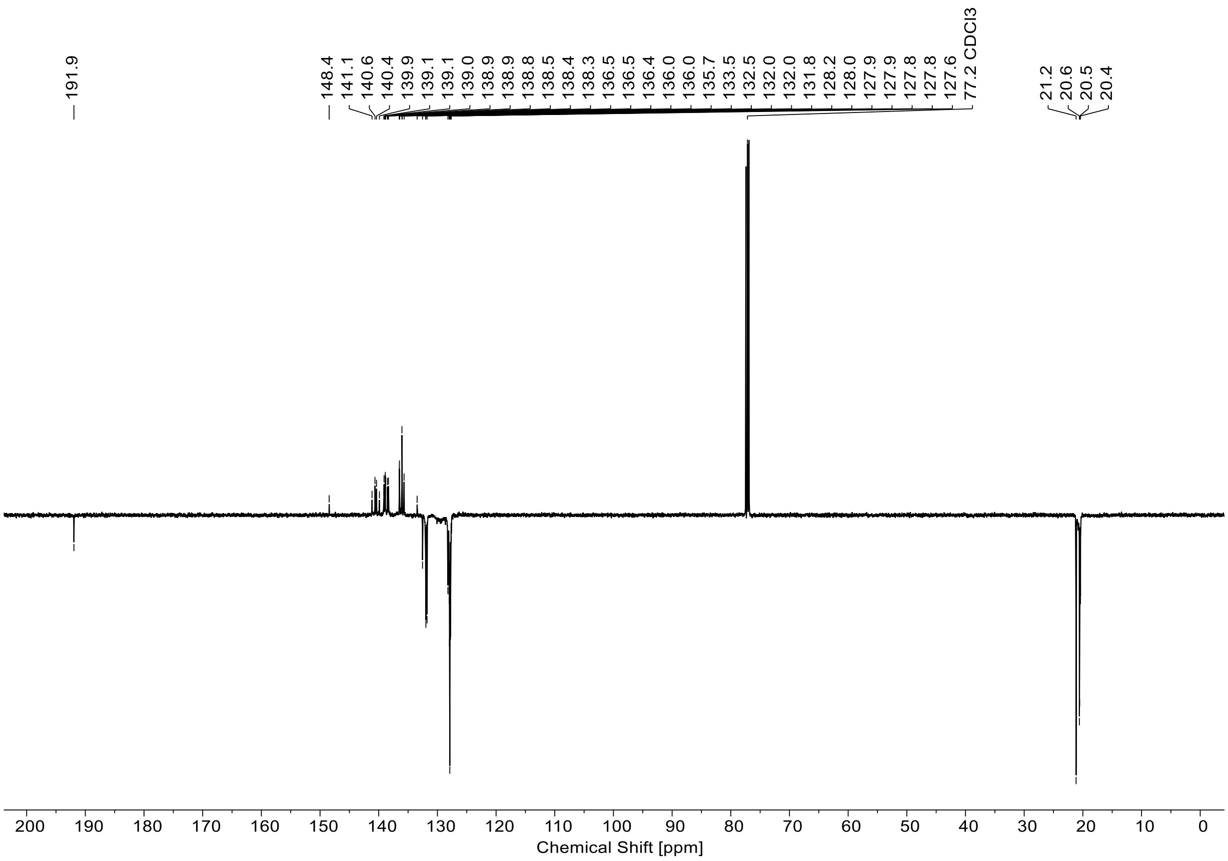

*Figure S12 Top: ^{1}H NMR (400 MHz, rt, $CDCl_3$) of **12**, stars indicate tiniest residues of DCM and water; bottom: DEPTq135 NMR (101 MHz, rt, $CDCl_3$) of **12**, inverted CH/CH_3 down, CH_2/Cquart. up.*

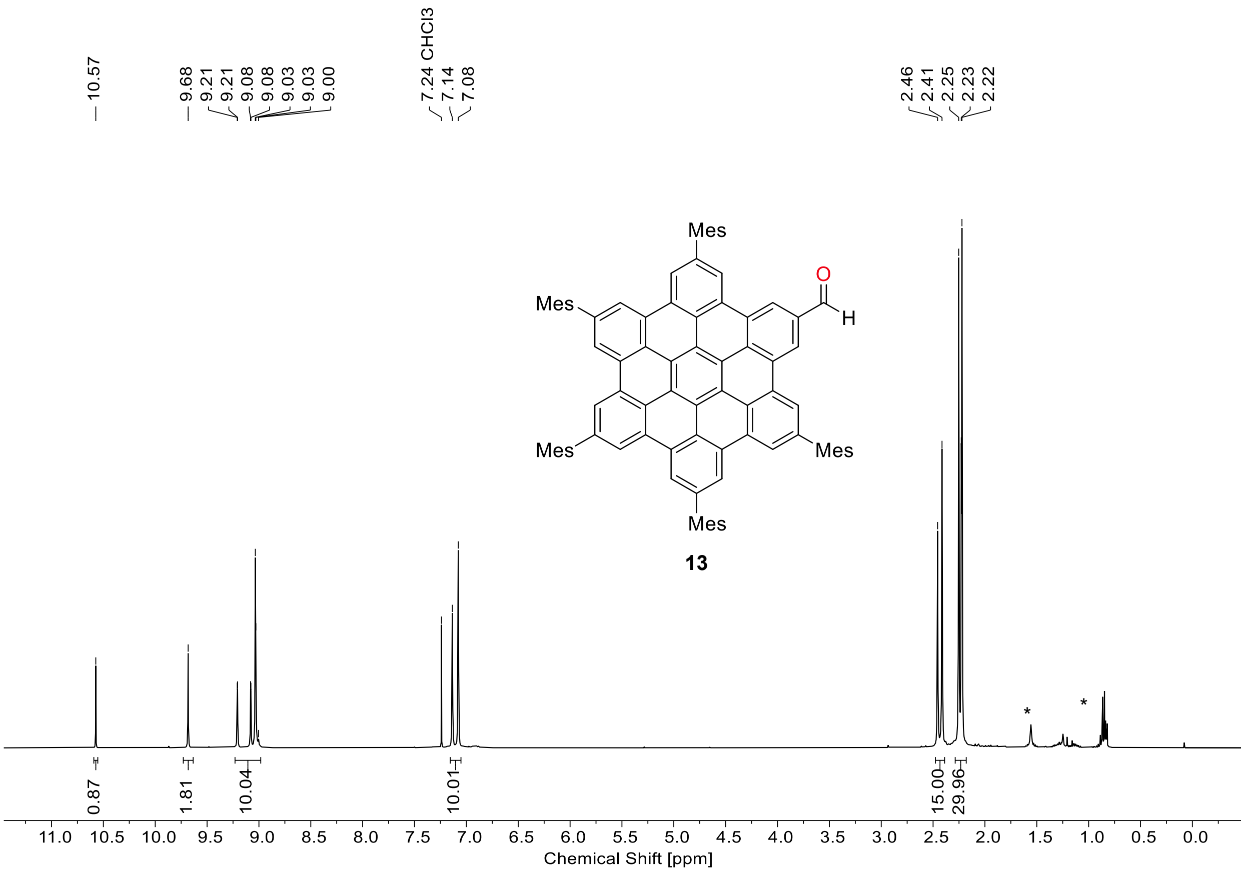

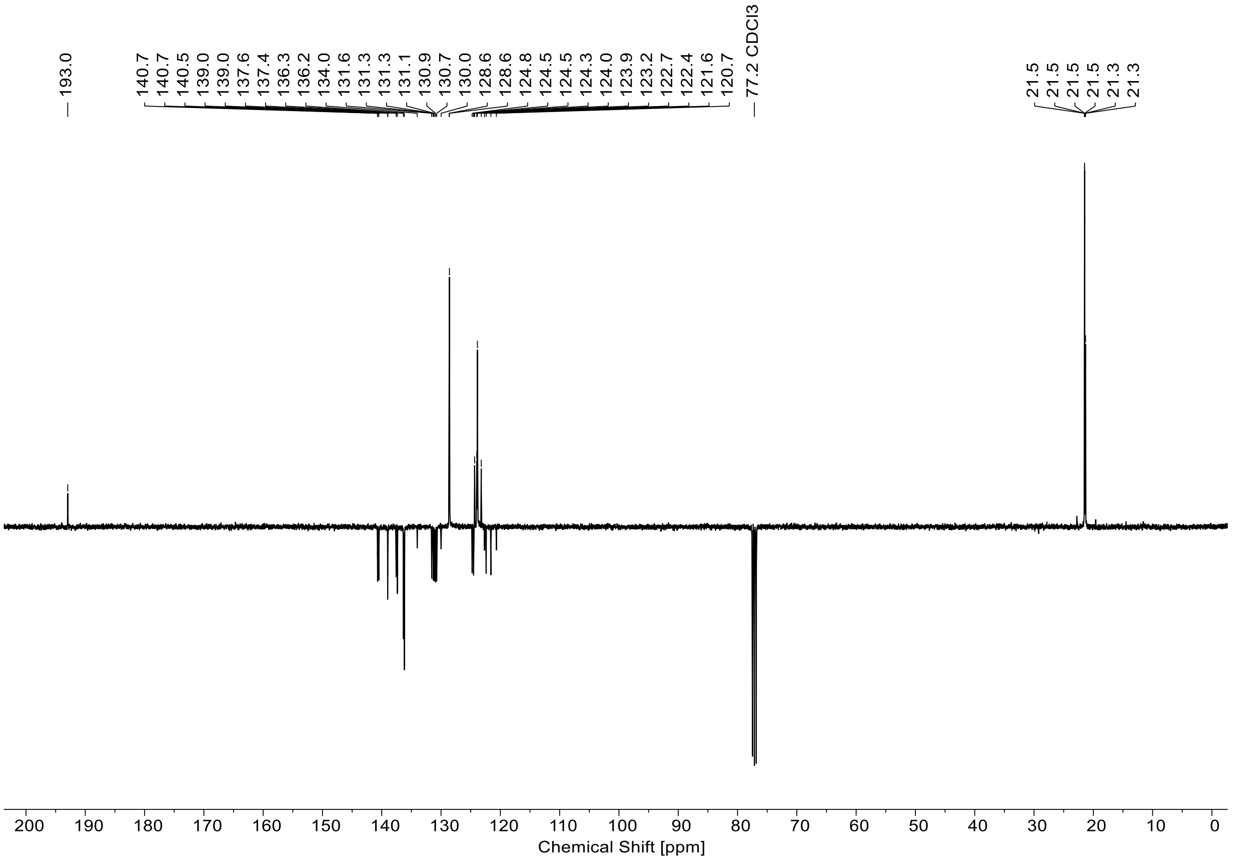

*Figure S13: ^{1}H NMR (400 MHz, rt, $CDCl_3$) of **13**, stars indicate small residues of water and grease; bottom: DEPTq135 NMR (101 MHz, rt, $CDCl_3$) of **13**, CH/CH_3 up, CH_2/Cquart. down.*

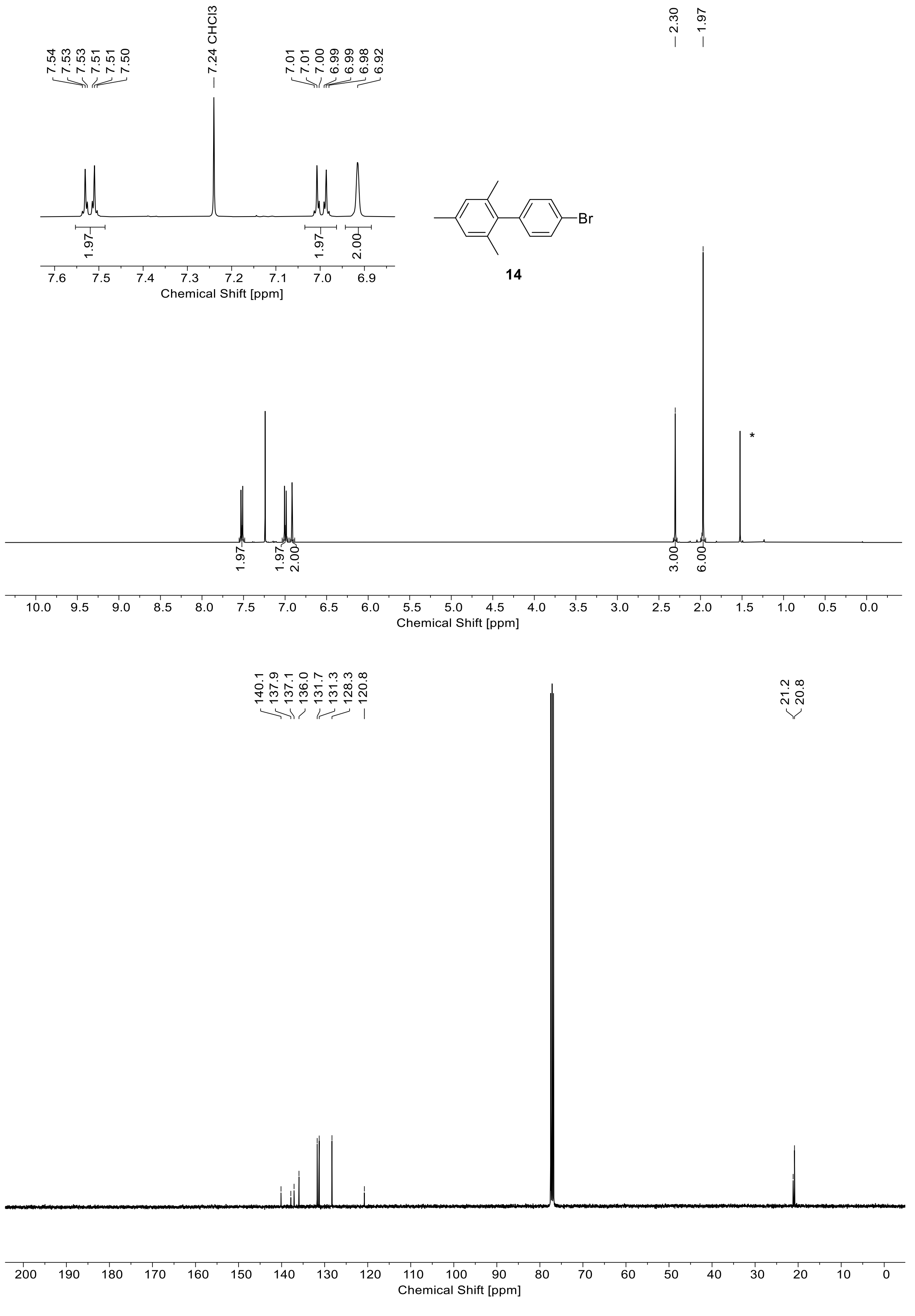

*Figure S14 Top: ^{1}H NMR (400 MHz, rt, $CDCl_3$) of **14**, the star indicates a residue of water; bottom: ^{13}C NMR (101 MHz, rt, $CDCl_3$) of **14**.*

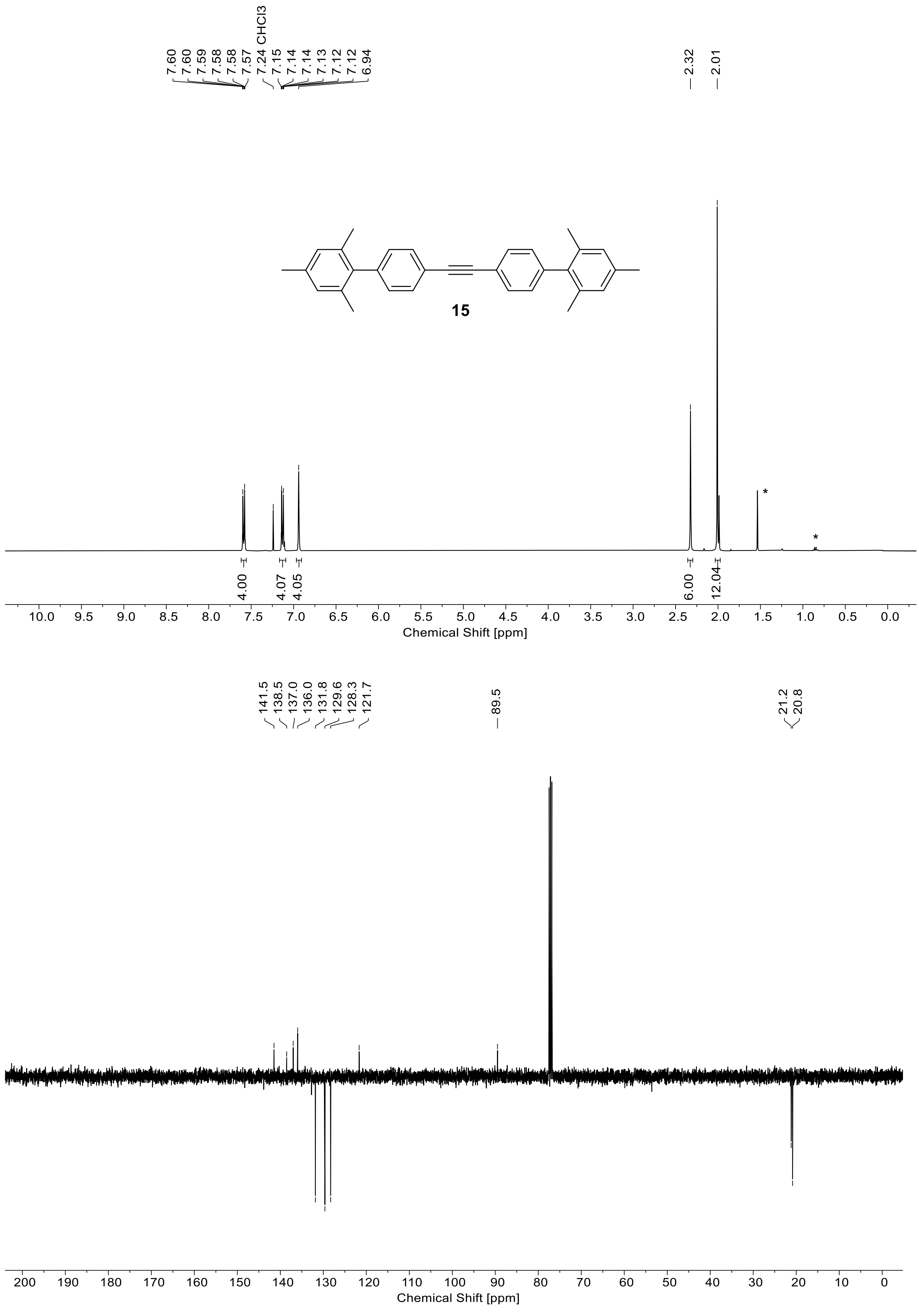

*Figure S15 Top: ^{1}H NMR (400 MHz, rt, $CDCl_3$) of **15**, stars indicate small residues of water and grease; bottom: inverted DEPTq135 NMR (101 MHz, rt, $CDCl_3$) of **15**, CH/CH_3 down, CH_2/Cquart. up.*

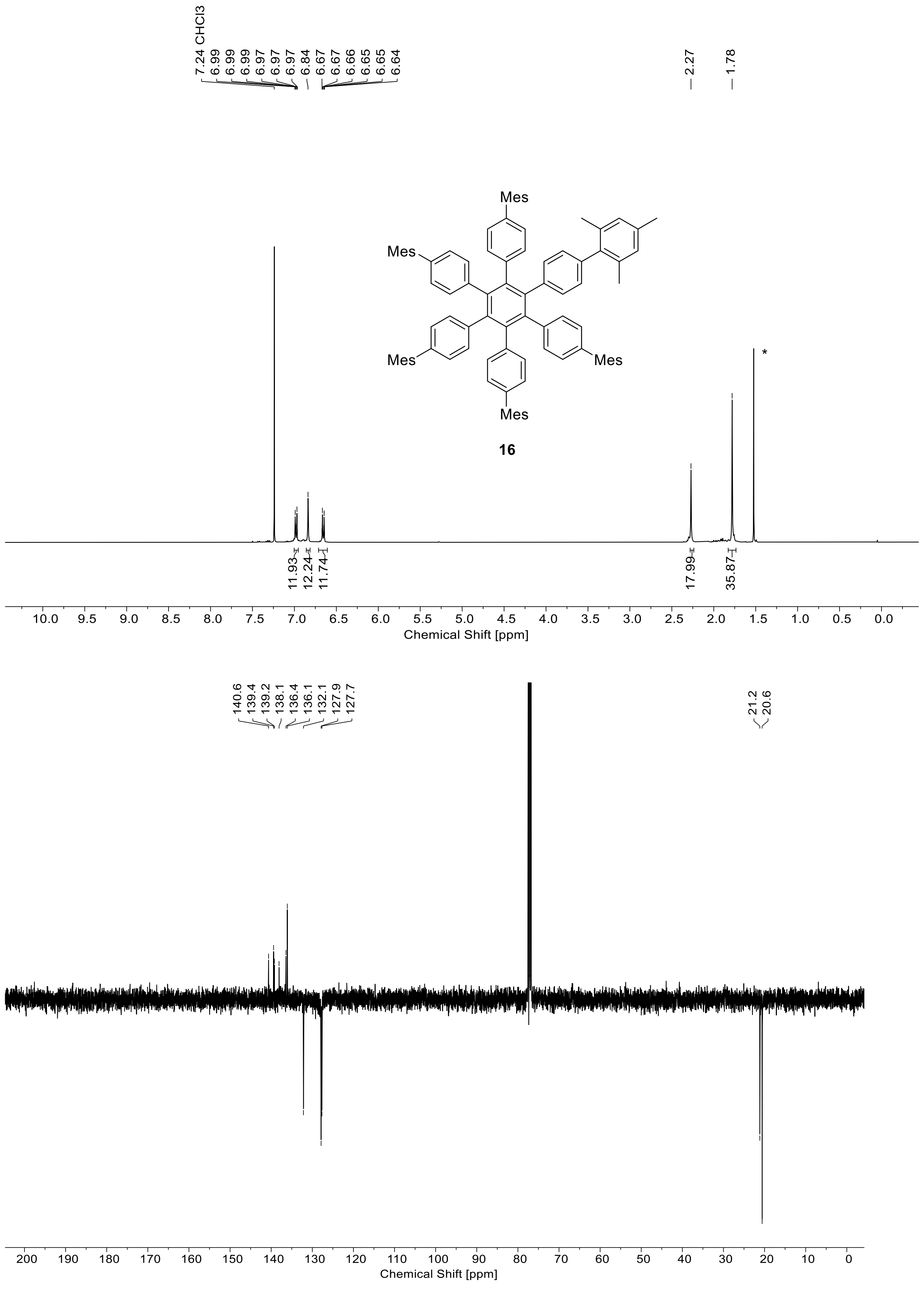

Figure S16 Top: ${}^{1}H$ NMR (400 MHz, rt, $CDCl_3$) of ***16****, stars indicate small residues of water and silicone grease; bottom: inverted DEPTQ135 NMR (101 MHz, rt, $CDCl_3$) of* ***16****, CH/CH_3 down, CH_2/Cquart. up.*

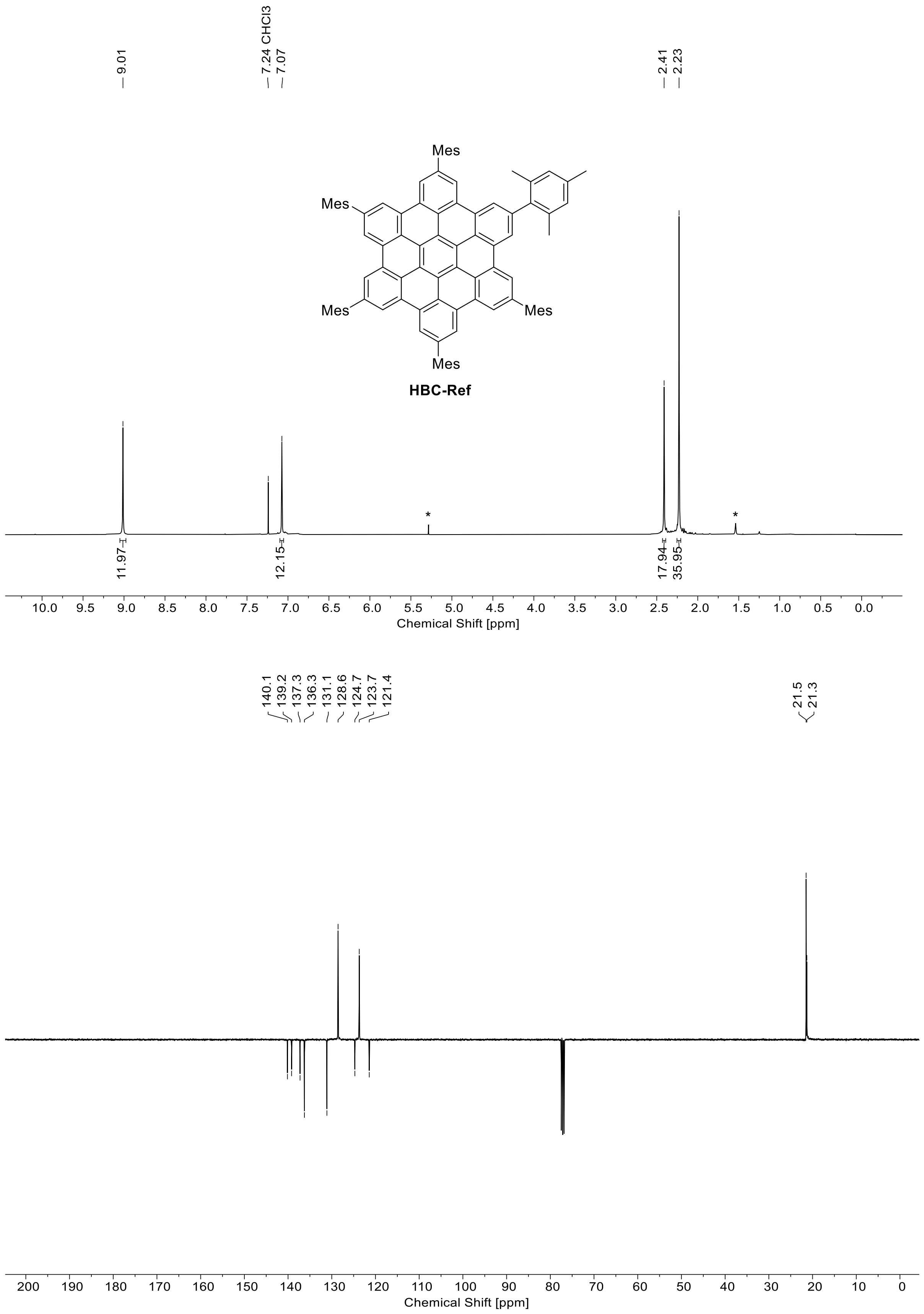

*Figure S17 Top: ^{1}H NMR (400 MHz, rt, $CDCl_3$) of **HBC-Ref**, stars indicate small residues of DCM and water; bottom: DEPTQ135 NMR (101 MHz, rt, $CDCl_3$) of **HBC-Ref**, CH/CH_3 up, CH_2/Cquart. down.*

MS spectra

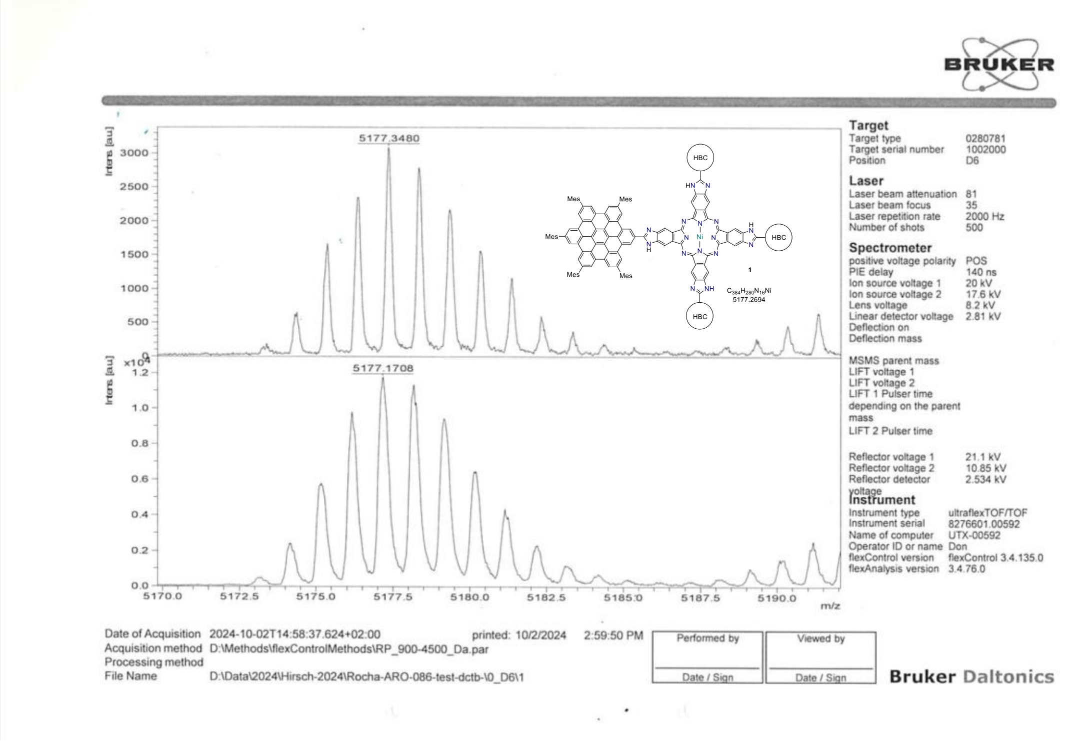

*Figure S18: MS (MALDI) of HBC-Pc **1**.*

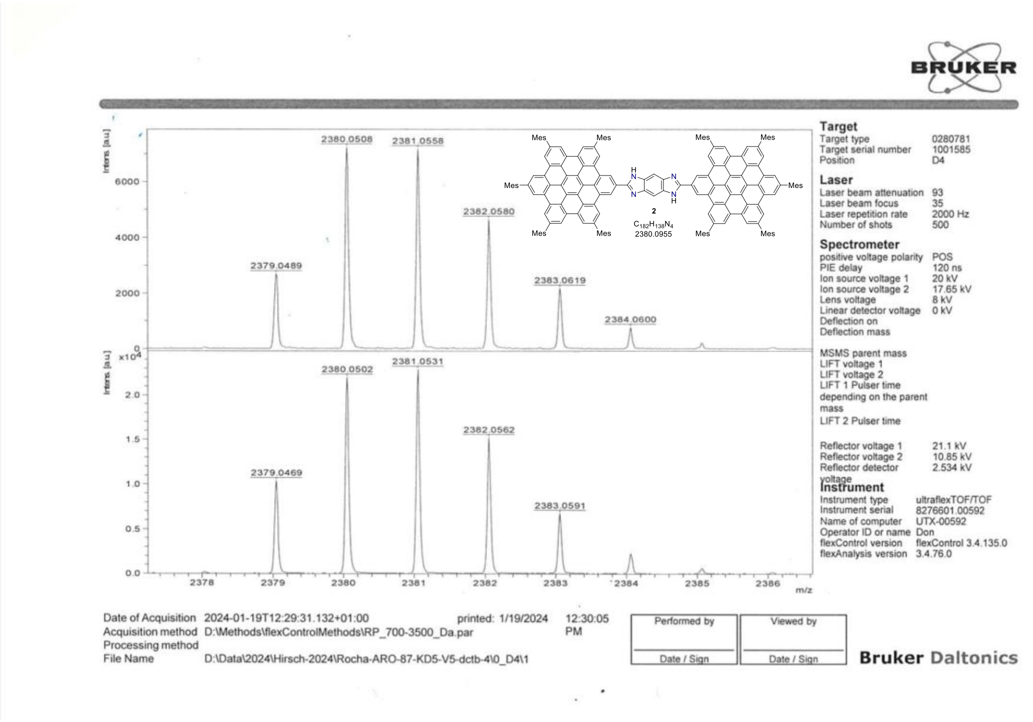

*Figure S19: MS (MALDI) of HBC **2**.*

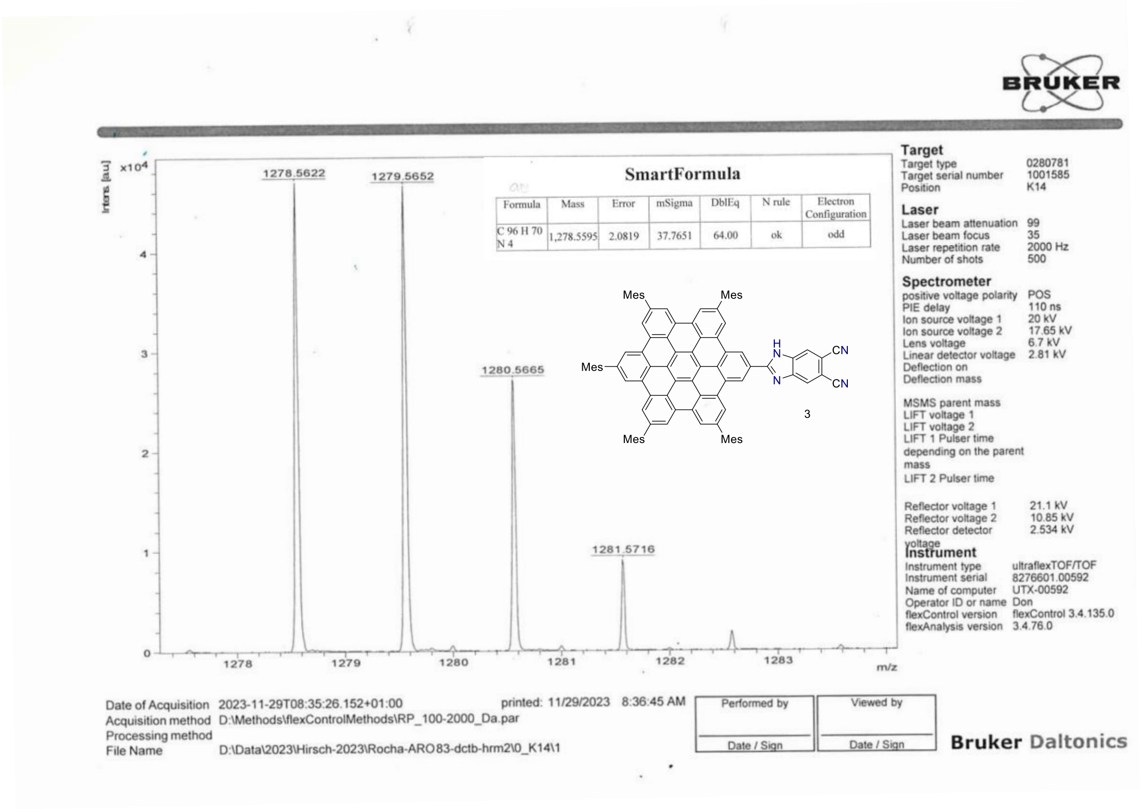

*Figure S20: HRMS (MALDI) of HBC **3**.*

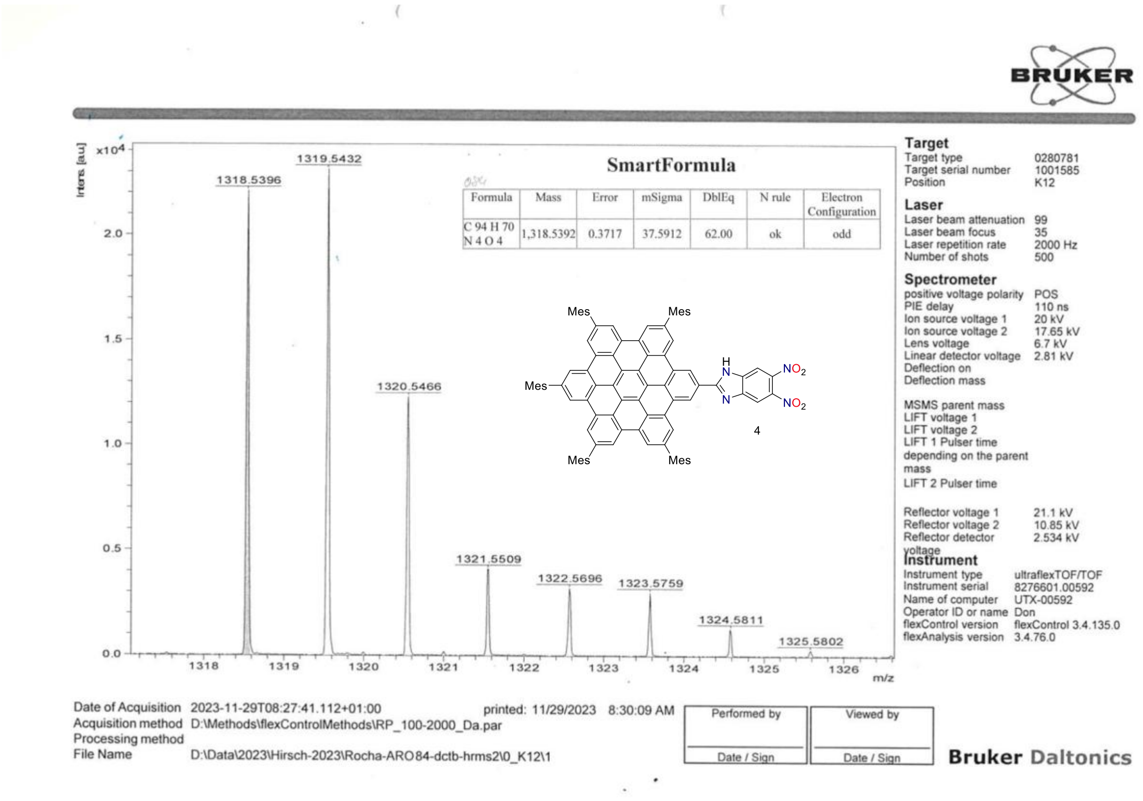

*Figure S21: HRMS (MALDI) of HBC **4**.*

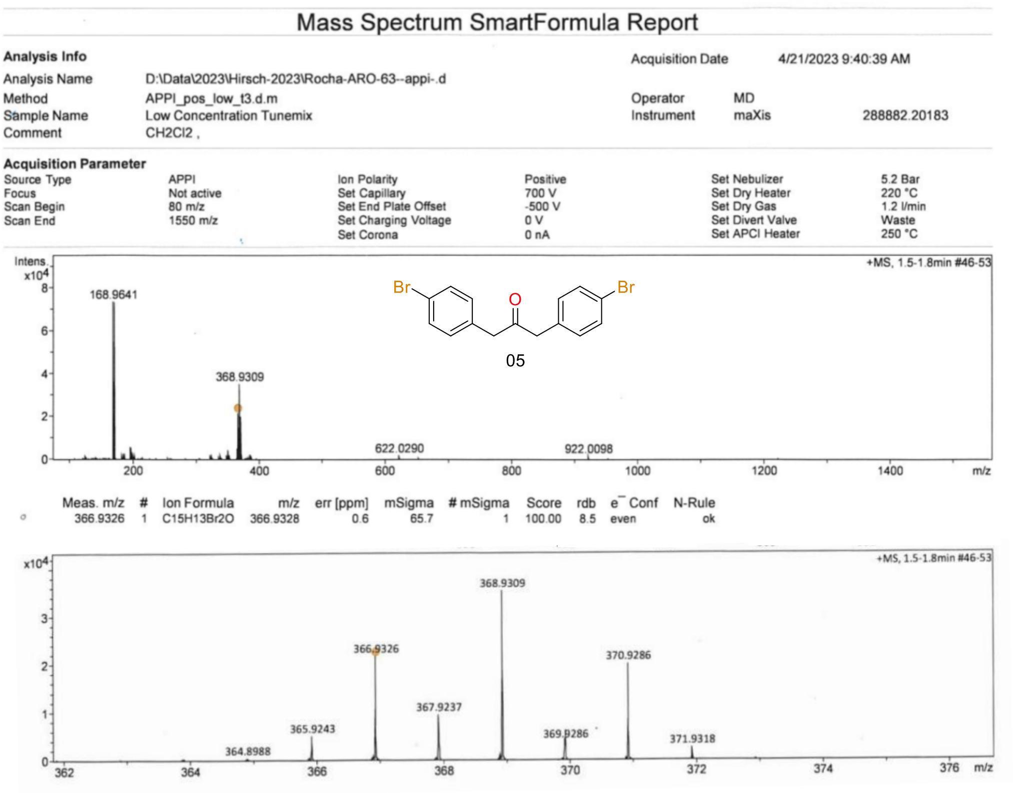

*Figure S22: HRMS (APPI) of molecule **5**.*

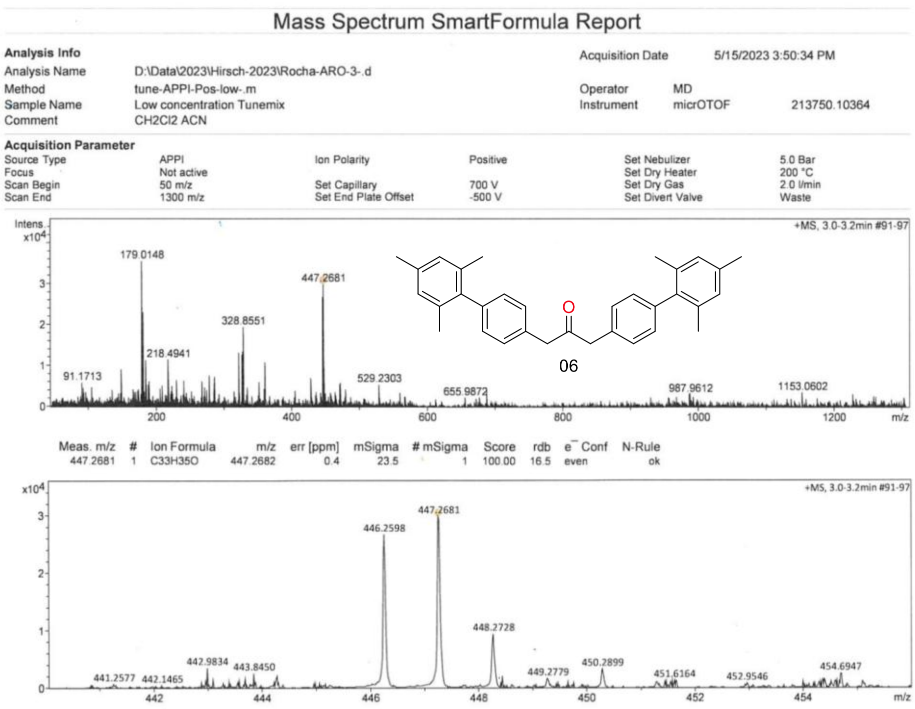

*Figure S23: HRMS (APPI) of molecule **6**.*

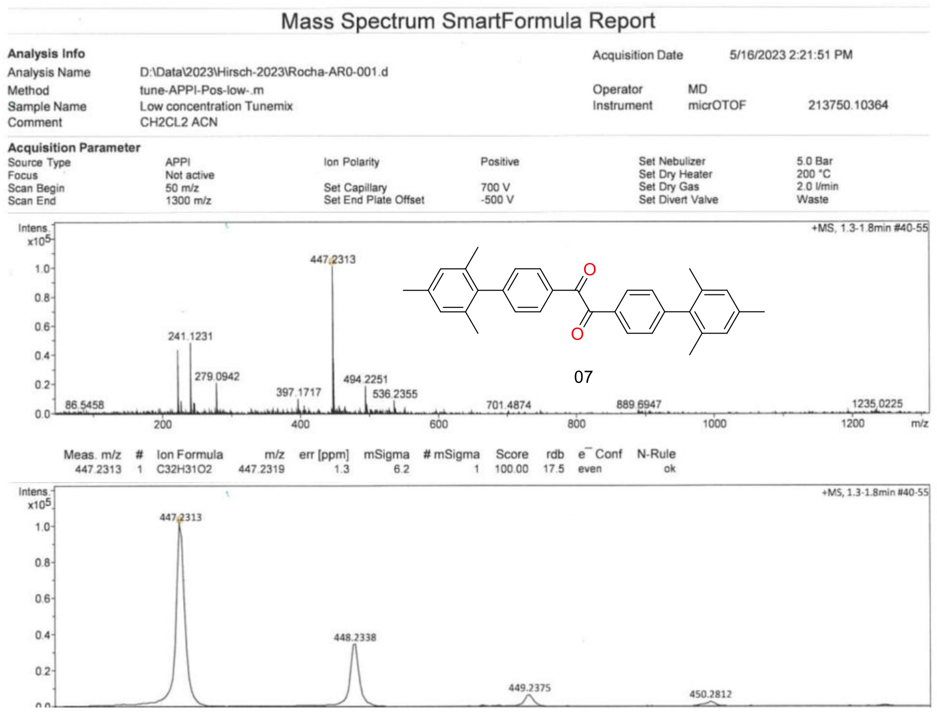

Figure S24: HRMS (APPI) of molecule **7**.

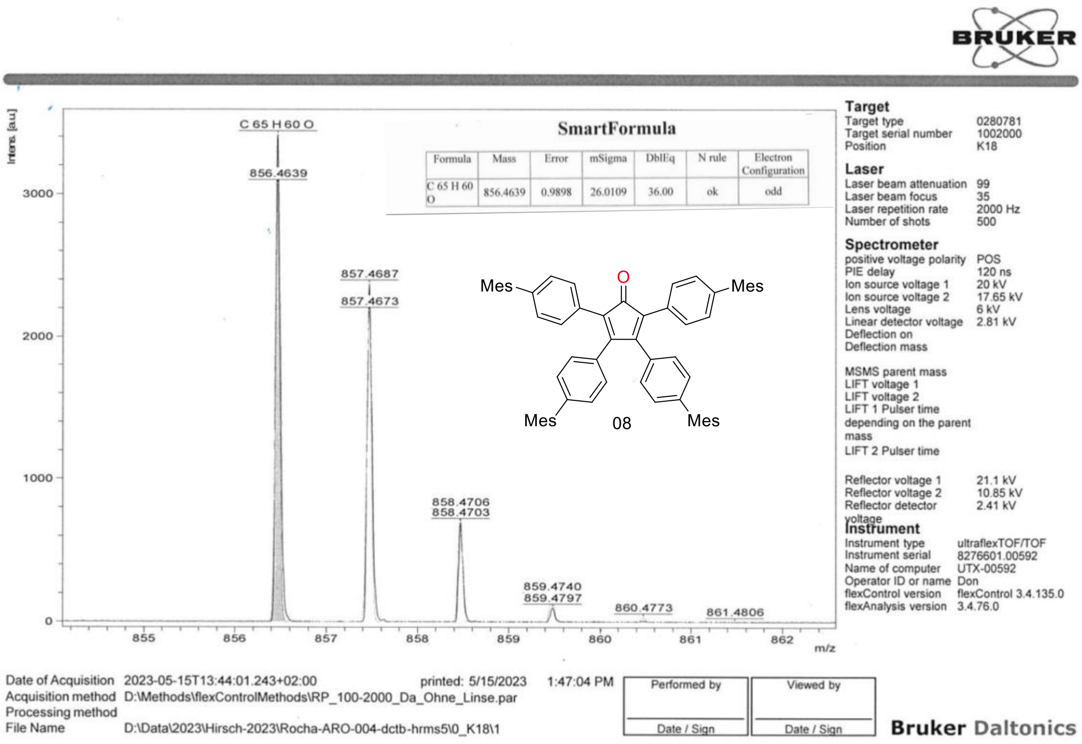

Figure S25: HRMS (MALDI) of molecule **8**.

Mass Spectrum SmartFormula Report

Analysis Info

Analysis Name	D:\Data\2025\Hirsch-2025\Rocha-ARO-011-appi-.d	Acquisition Date	4/14/2025 11:48:53 AM
Method	APPI_pos_low_t3.d.m	Operator	MD
Sample Name	Low Concentration Tunemix	Instrument	maXis 288882.20183
Comment	CH2CL2		

Acquisition Parameter

Source Type	APPI	Ion Polarity	Positive	Set Nebulizer	5.2 Bar
Focus	Not active	Set Capillary	700 V	Set Dry Heater	220 °C
Scan Begin	80 m/z	Set End Plate Offset	-500 V	Set Dry Gas	1.2 l/min
Scan End	1550 m/z	Set Charging Voltage	0 V	Set Divert Valve	Waste
		Set Corona	0 nA	Set APCI Heater	250 °C

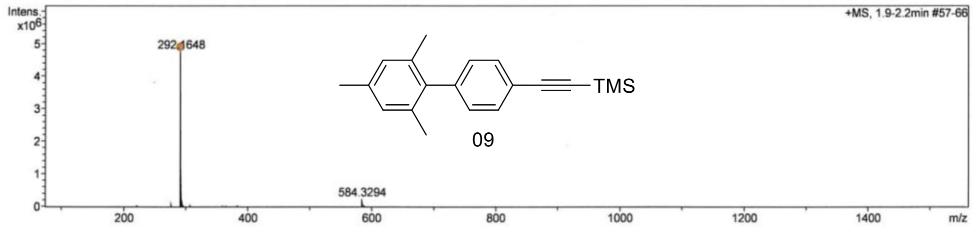

Meas. m/z	#	Ion Formula	m/z	err [ppm]	mSigma	# mSigma	Score	rdb	e⁻ Conf	N-Rule
292.1648	1	C20H24Si	292.1642	-2.2	42.6	1	100.00	10.0	odd	ok

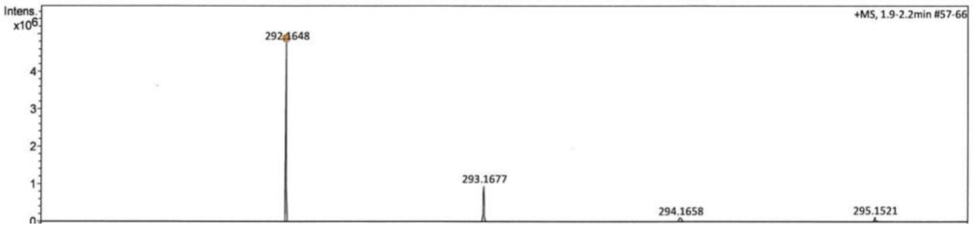

*Figure S26: HRMS (APPI) of molecule **9**.*

Mass Spectrum SmartFormula Report

Analysis Info

Analysis Name	D:\Data\2023\Hirsch-2023\Rocha-AR-15-.d	Acquisition Date	5/22/2023 2:37:54 PM
Method	tune-APPI-Pos-low-.m	Operator	MD
Sample Name	Low concentration Tunemix	Instrument	micrOTOF 213750.10364
Comment	CH2Cl2		

Acquisition Parameter

Source Type	APPI	Ion Polarity	Positive	Set Nebulizer	4.0 Bar
Focus	Not active			Set Dry Heater	200 °C
Scan Begin	50 m/z	Set Capillary	700 V	Set Dry Gas	2.0 l/min
Scan End	1300 m/z	Set End Plate Offset	-500 V	Set Divert Valve	Waste

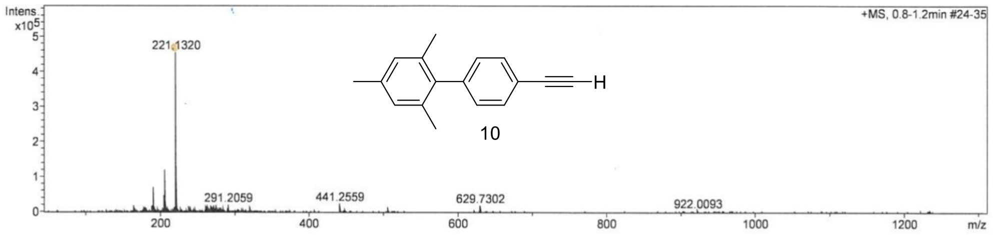

Meas. m/z	#	Ion Formula	m/z	err [ppm]	mSigma	# mSigma	Score	rdb	e⁻ Conf	N-Rule
221.1320	1	C17H17	221.1325	2.2	9.5	1	100.00	9.5	even	ok

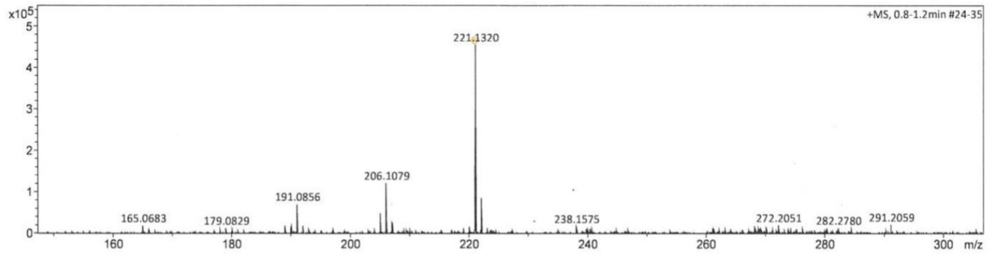

*Figure S27: HRMS (APPI) of molecule **10**.*

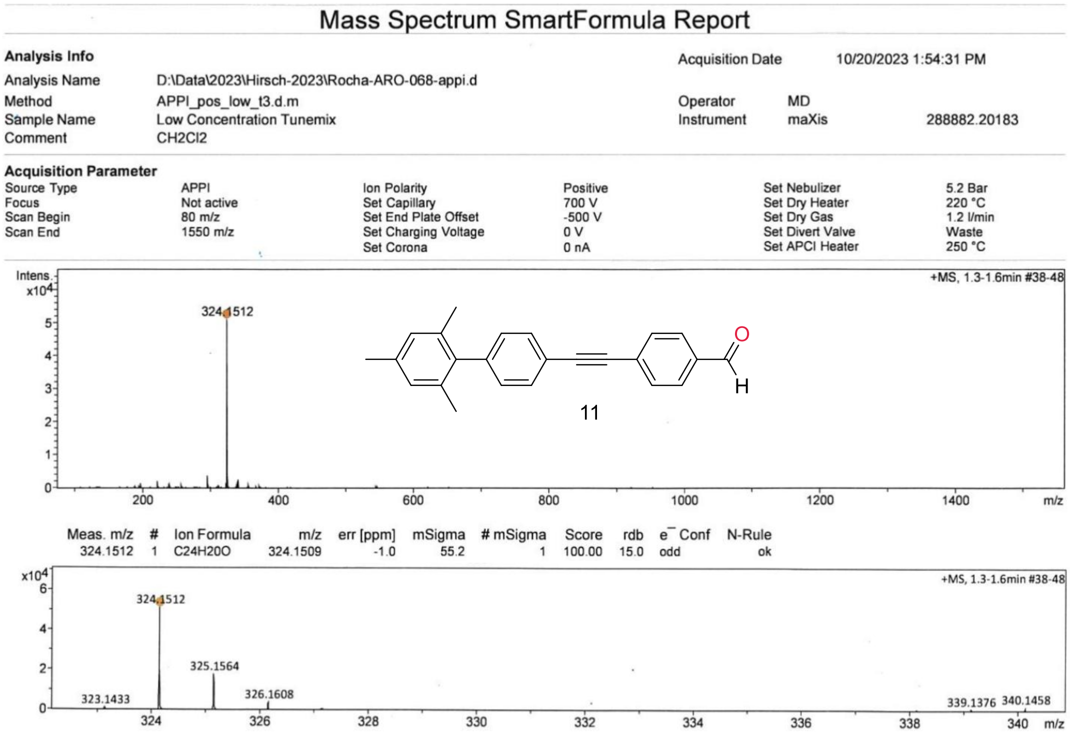

*Figure S28: HRMS (APPI) of molecule **11**.*

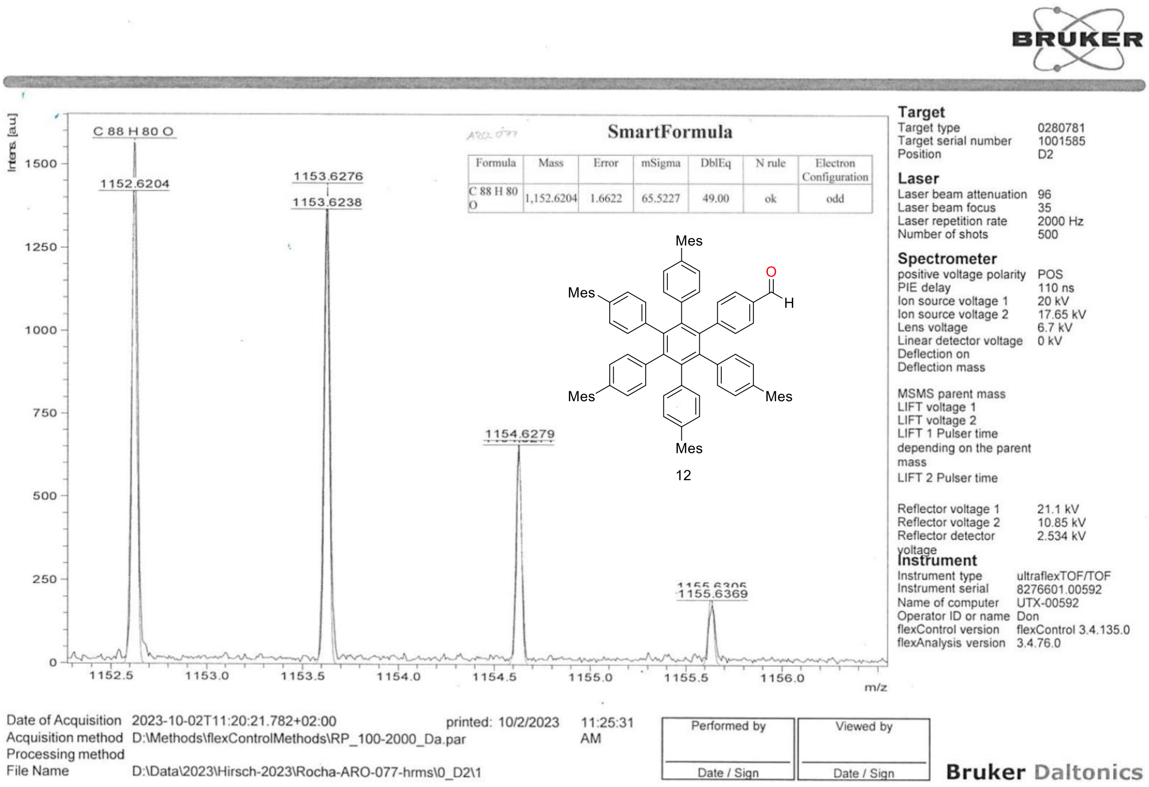

*Figure S29: HRMS (MALDI) of molecule **12**.*

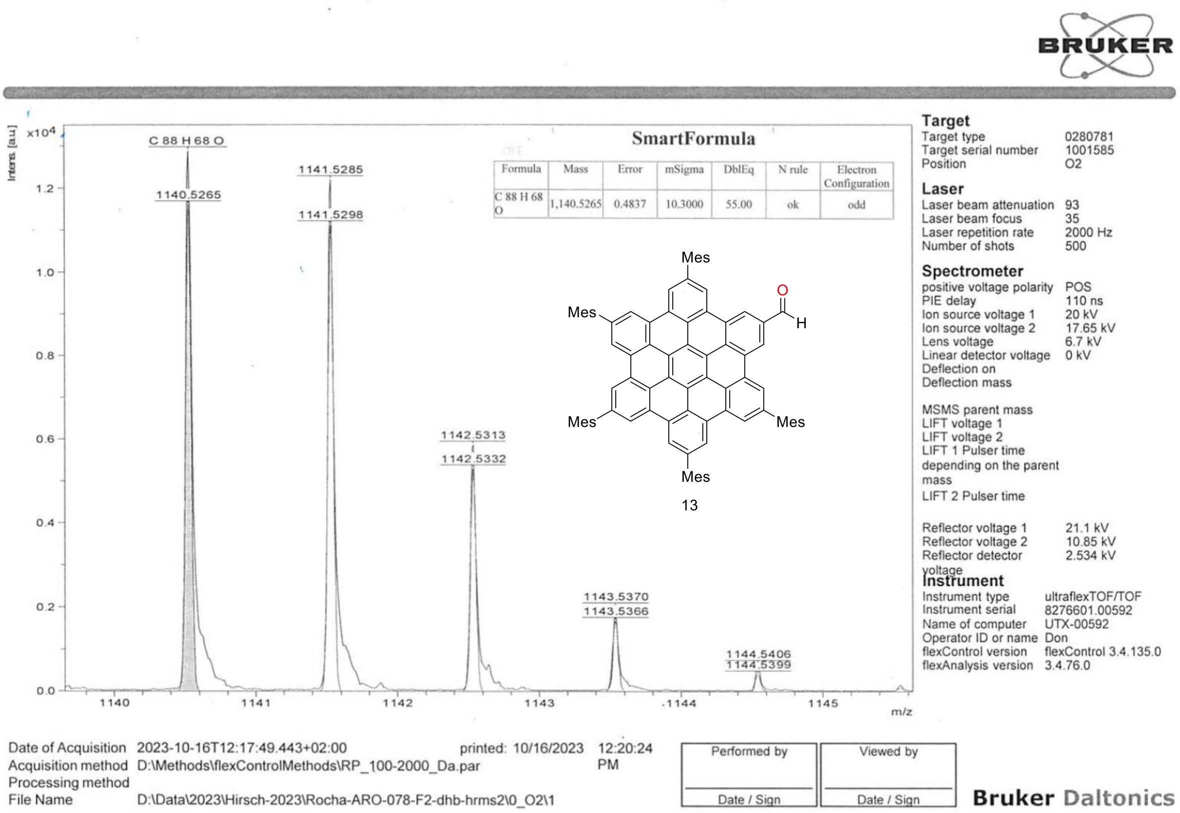

*Figure S30: HRMS (MALDI) of molecule **13**.*

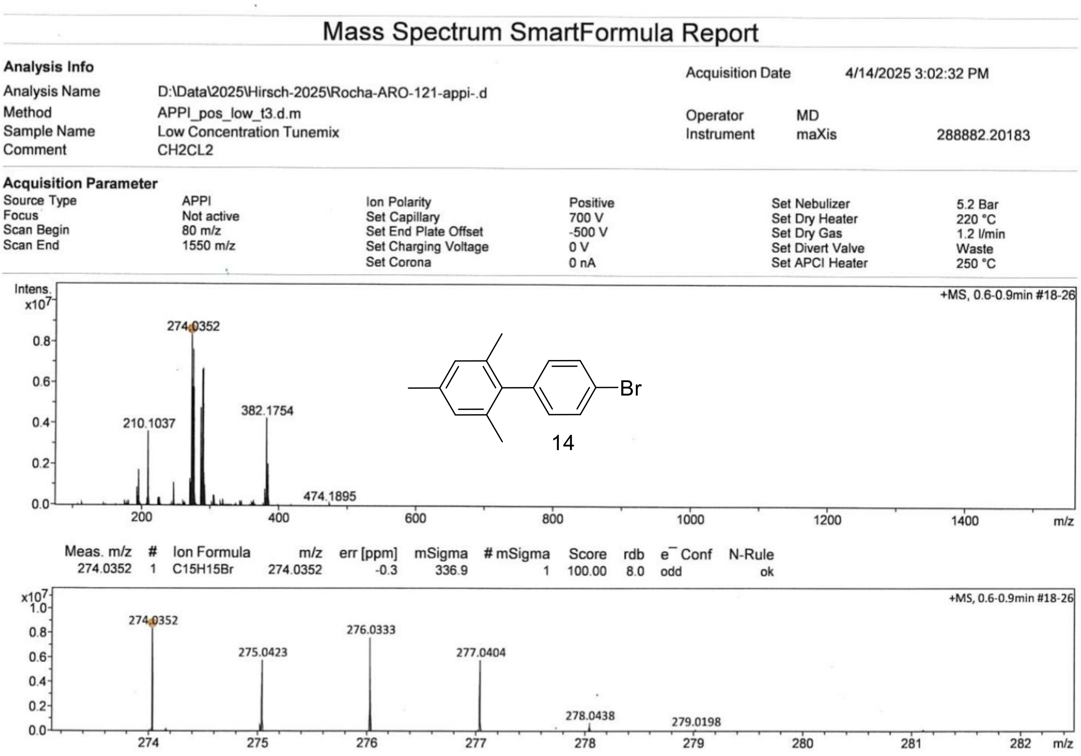

*Figure S31: HRMS (APPI) of molecule **14**.*

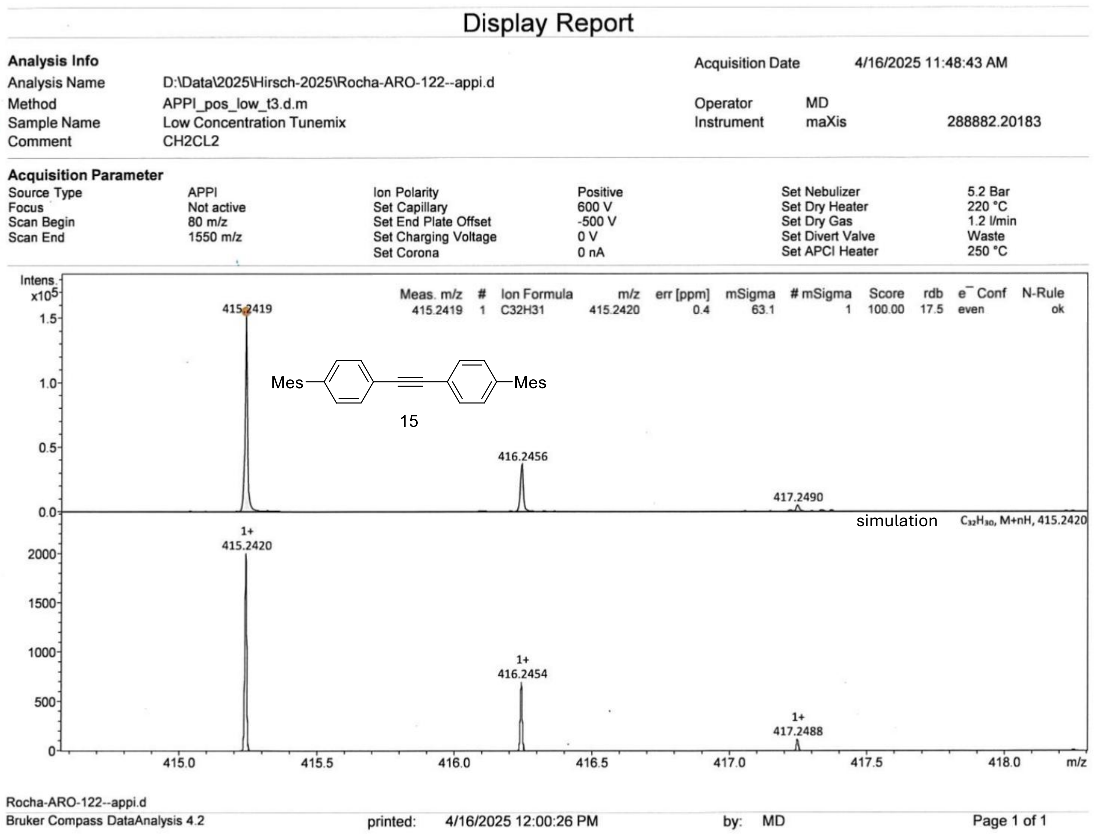

*Figure S32: HRMS (APPI) of molecule **15**.*

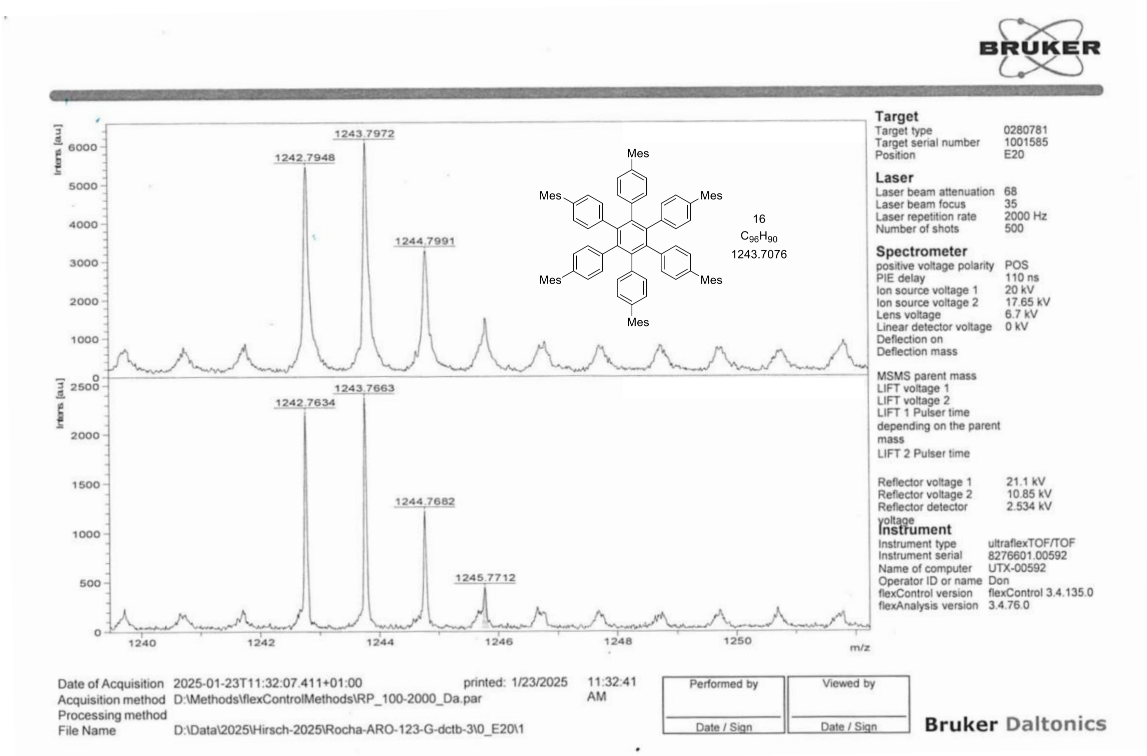

*Figure S33: MS (MALDI) of molecule **16**.*

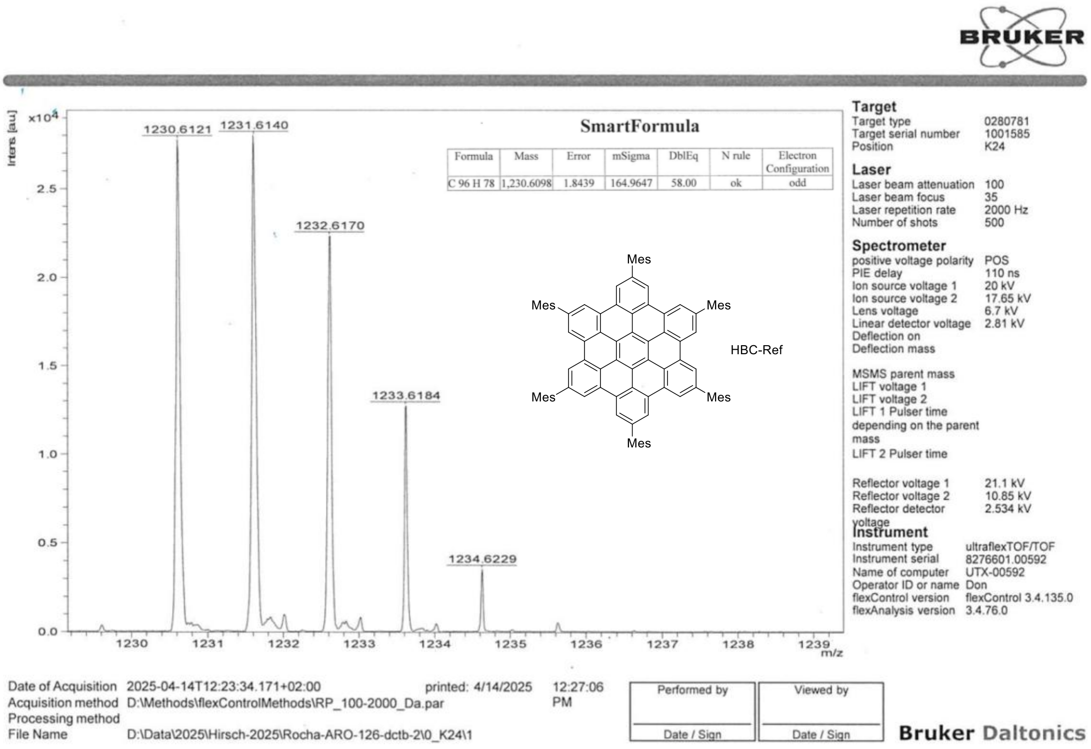

*Figure S34: HRMS (MALDI) of **HBC-Ref**.*

IR spectra

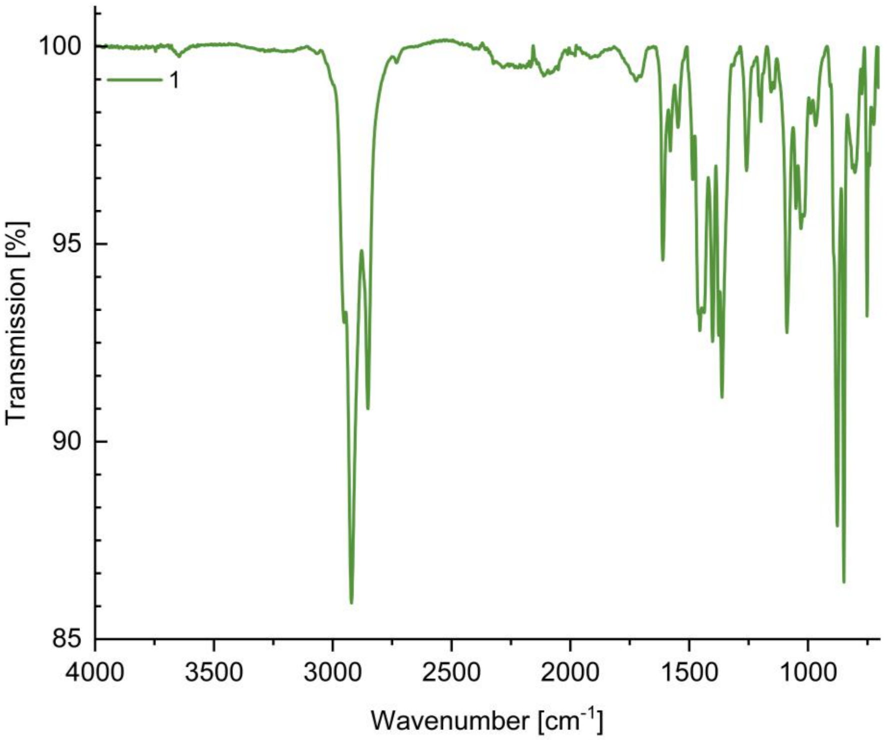

*Figure S35: ATR-IR spectrum of HBC-Pc **1**.*

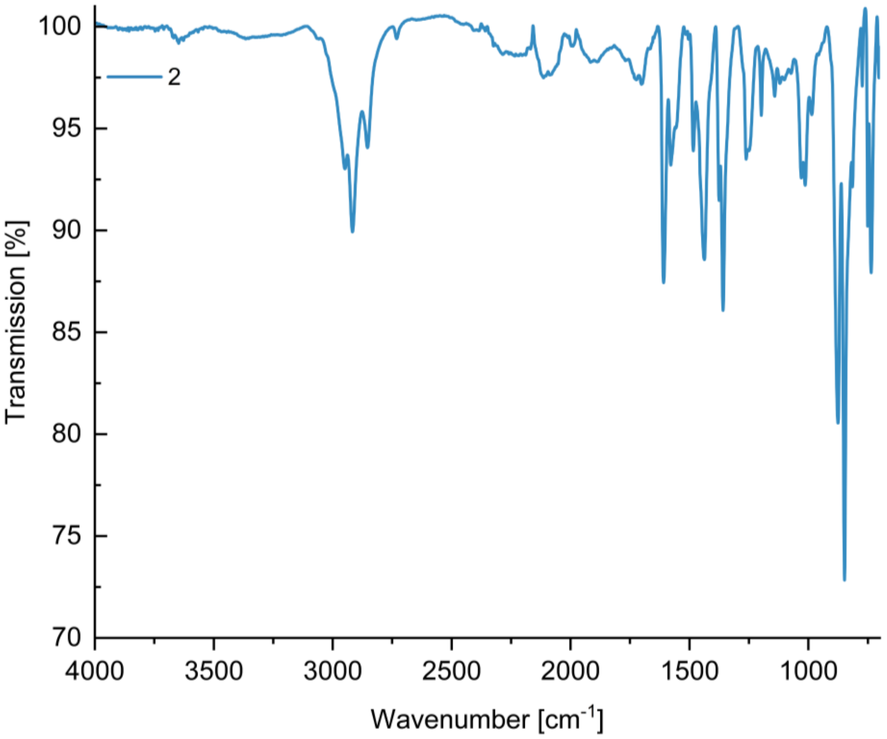

*Figure S36: ATR-IR spectrum of HBC **2**.*

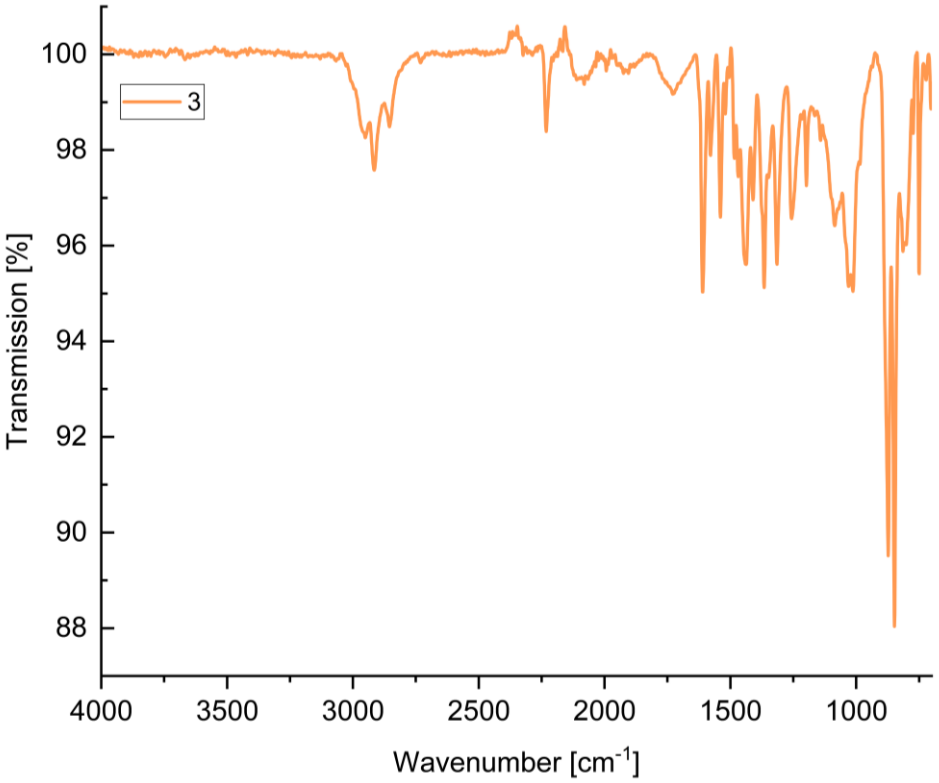

*Figure S37: ATR-IR spectrum of HBC **3**.*

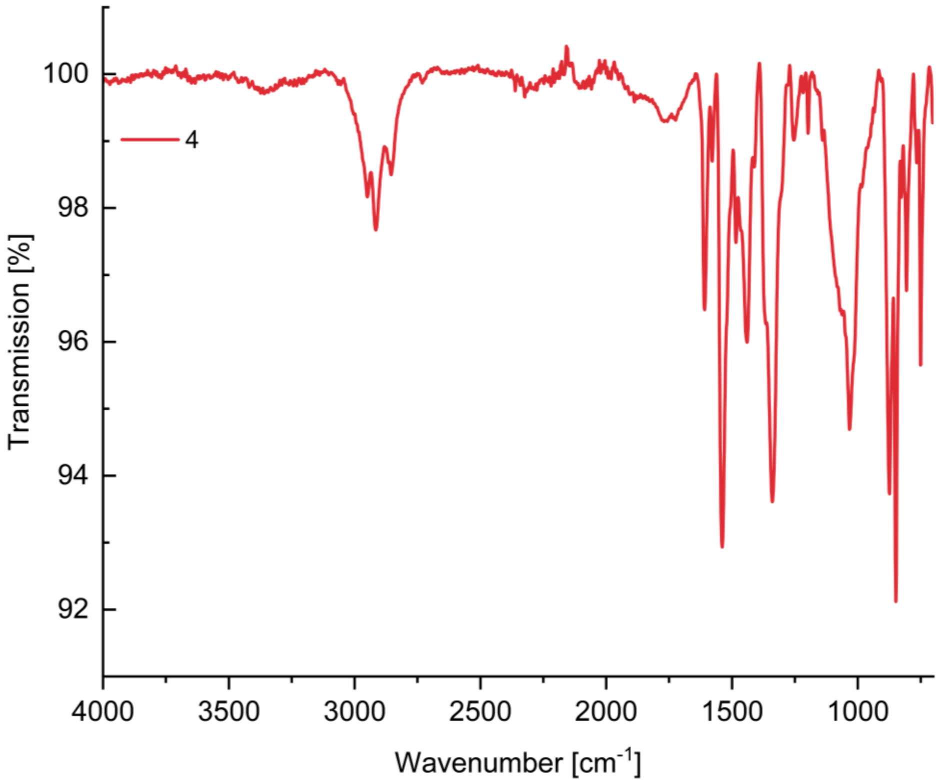

*Figure S38: ATR-IR spectrum of HBC **4**.*

Steady state absorption and emission measurements

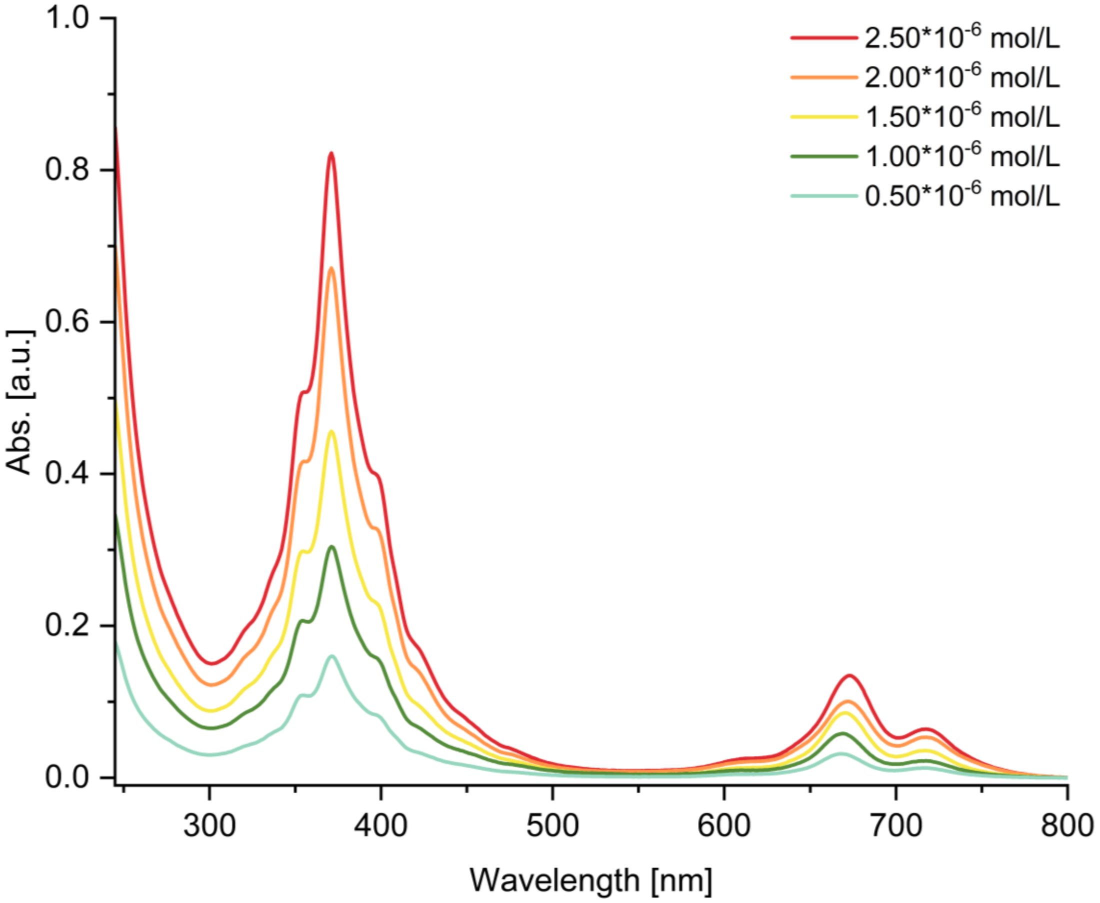

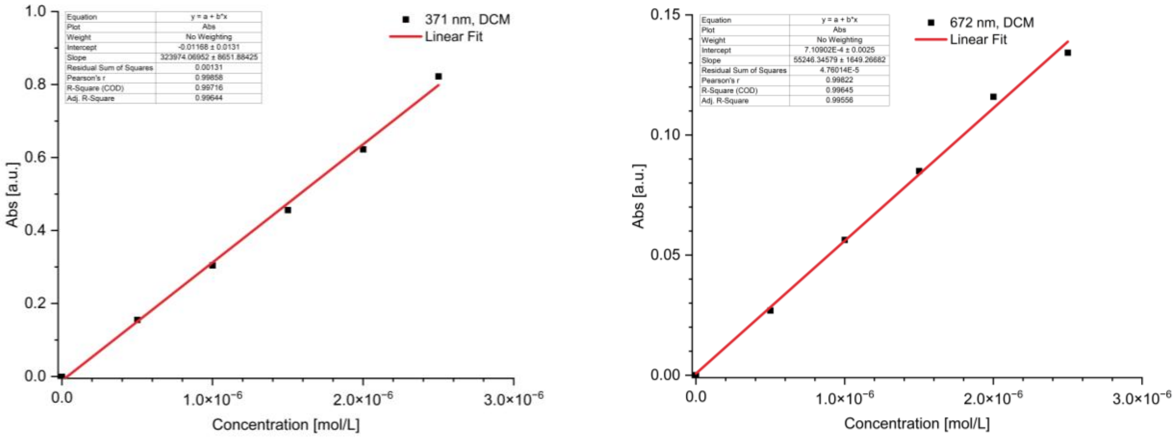

*Figure S39-1 (top): Steady state absorption spectra of HBC-Pc **1** in DCM: dilution row; S39-2 (bottom): resulting linear fits at the respective global and local maxima, left (371 nm) to right (672 nm);*

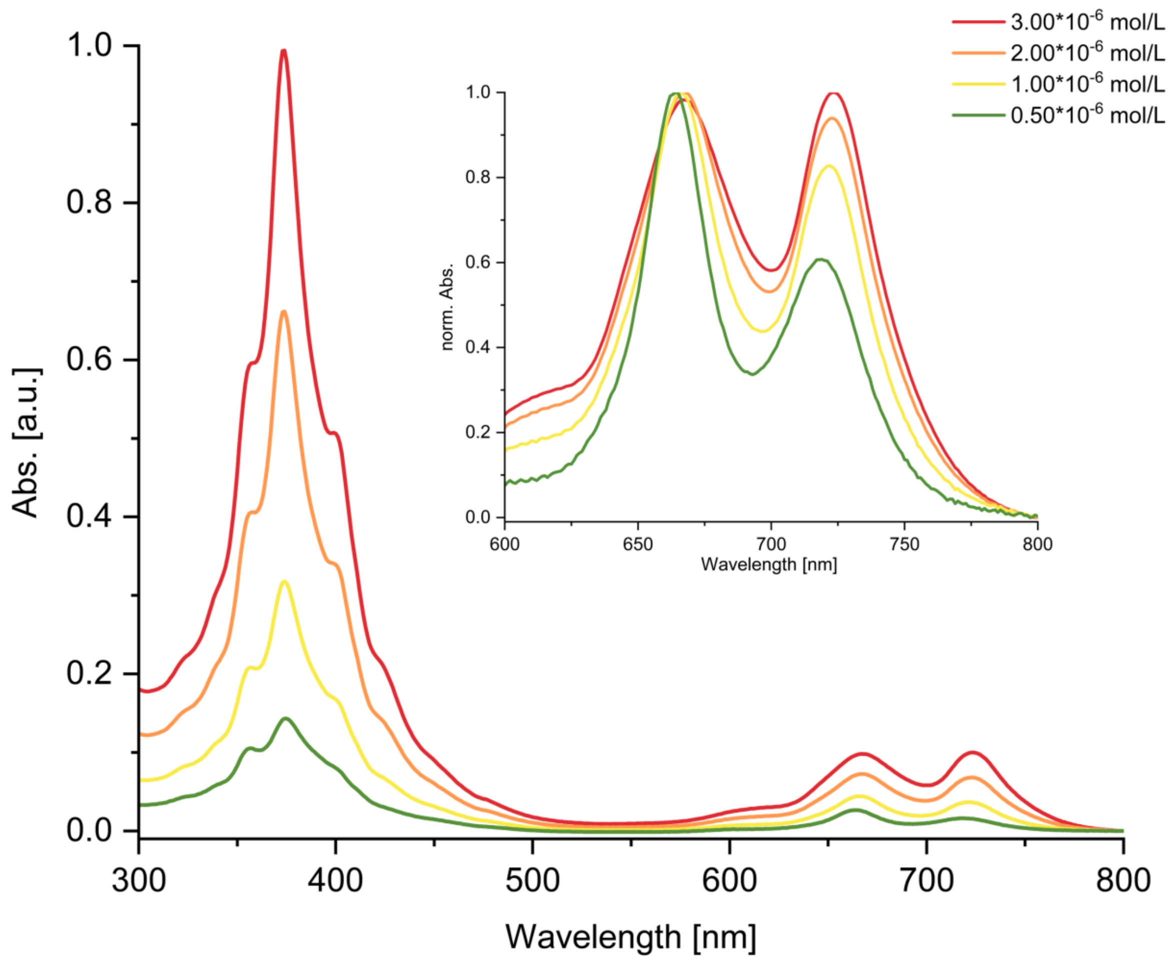

*S39-3: Steady state absorption spectra of HBC-Pc **1** in 1,2-dichlorobenzene: dilution row (large) and normalized spectra of the Pc Q-bands between 600-800 nm to highlight the concentration dependence of the second absorption band at 720 nm, which thus is likely caused by aggregation (inset).*

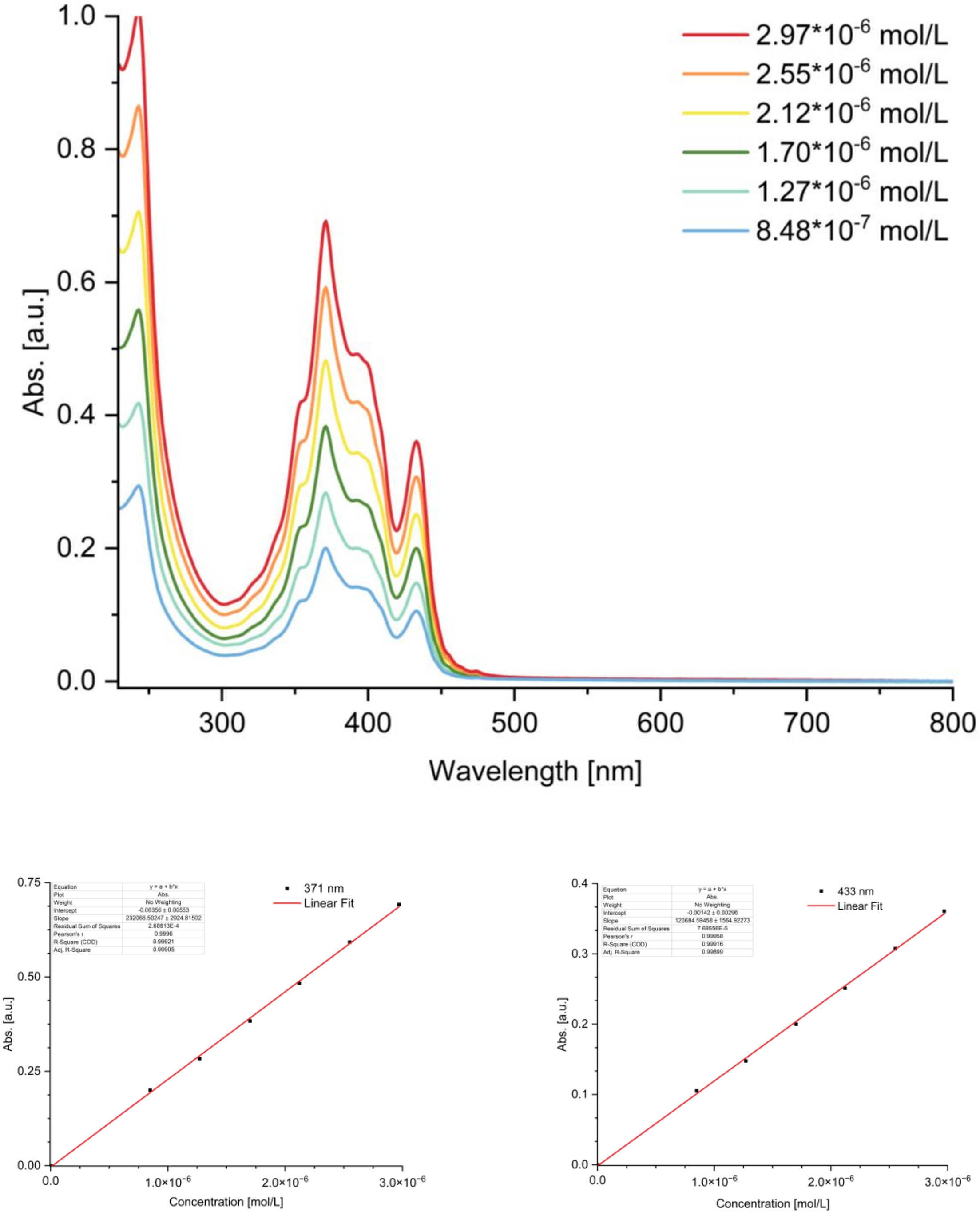

Figure S40: Steady state absorption spectra of HBC **2** *in DCM: dilution row (top) and resulting linear fits at the respective global and local maxima, left (371 nm) to right (433n nm).*

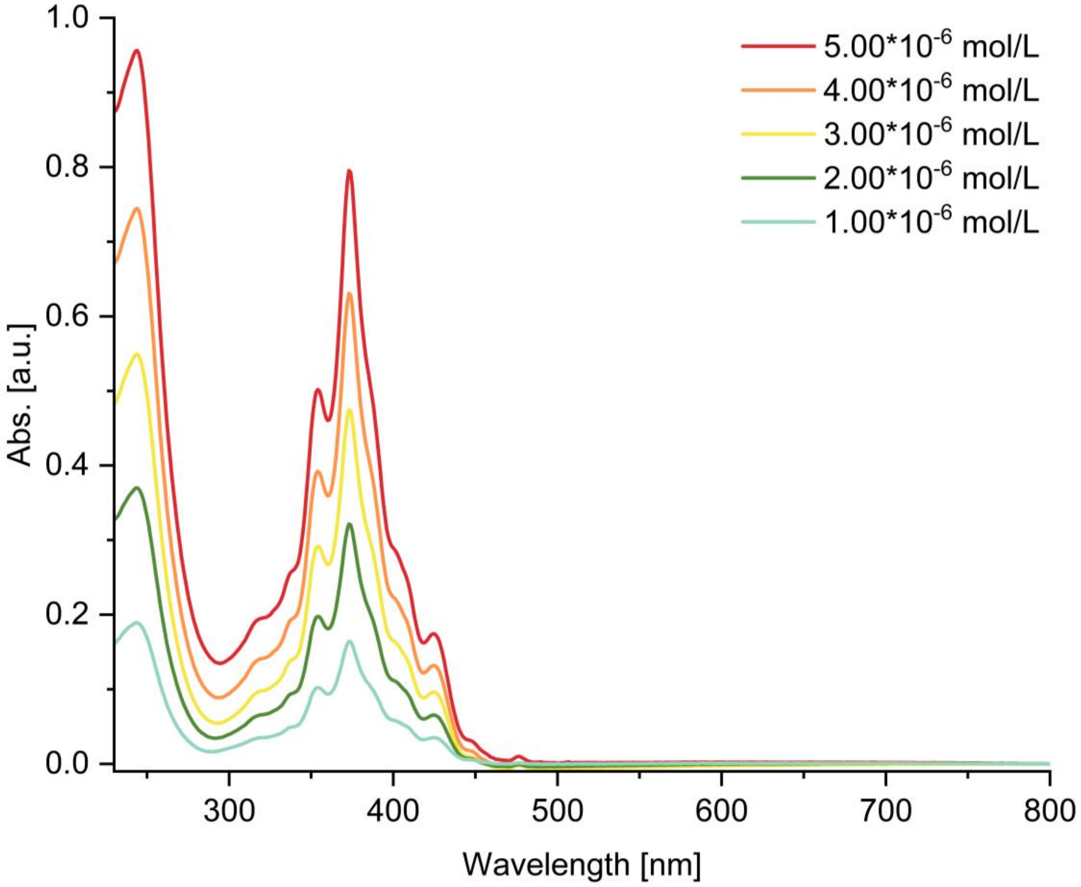

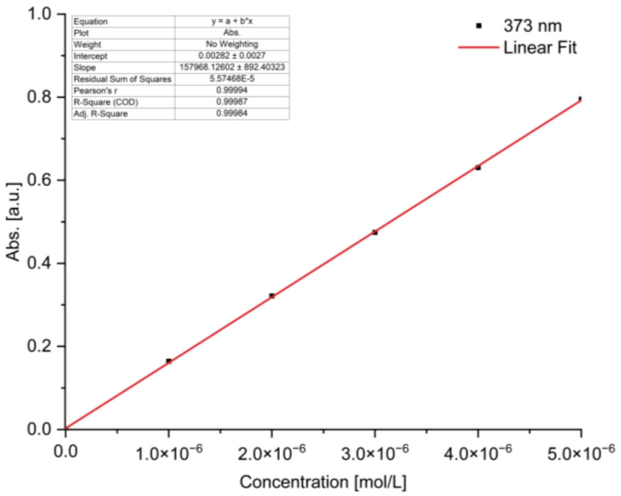

Figure S41: Steady state absorption spectra of HBC ***3*** *in DCM: dilution row (top) and resulting linear fit at the global maximum, 373 nm.*

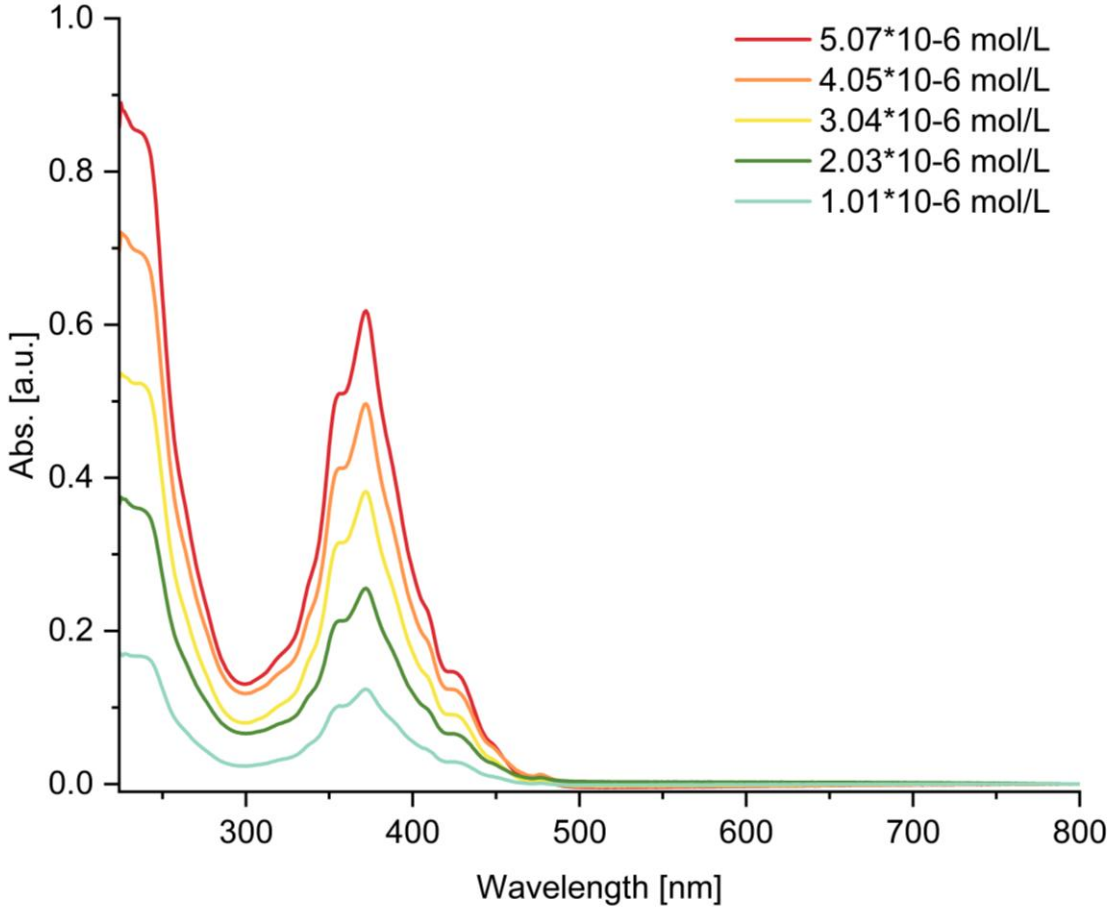

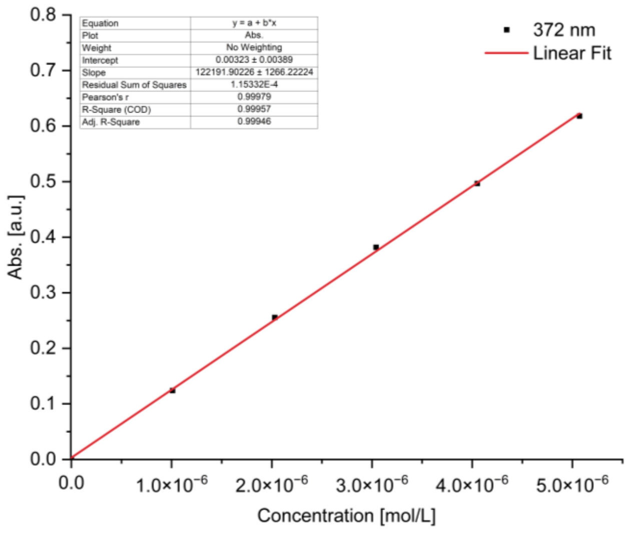

*Figure S42: Steady state absorption spectra of HBC **4** in DCM: dilution row (top) and resulting linear fit at the global maximum, 372 nm.*

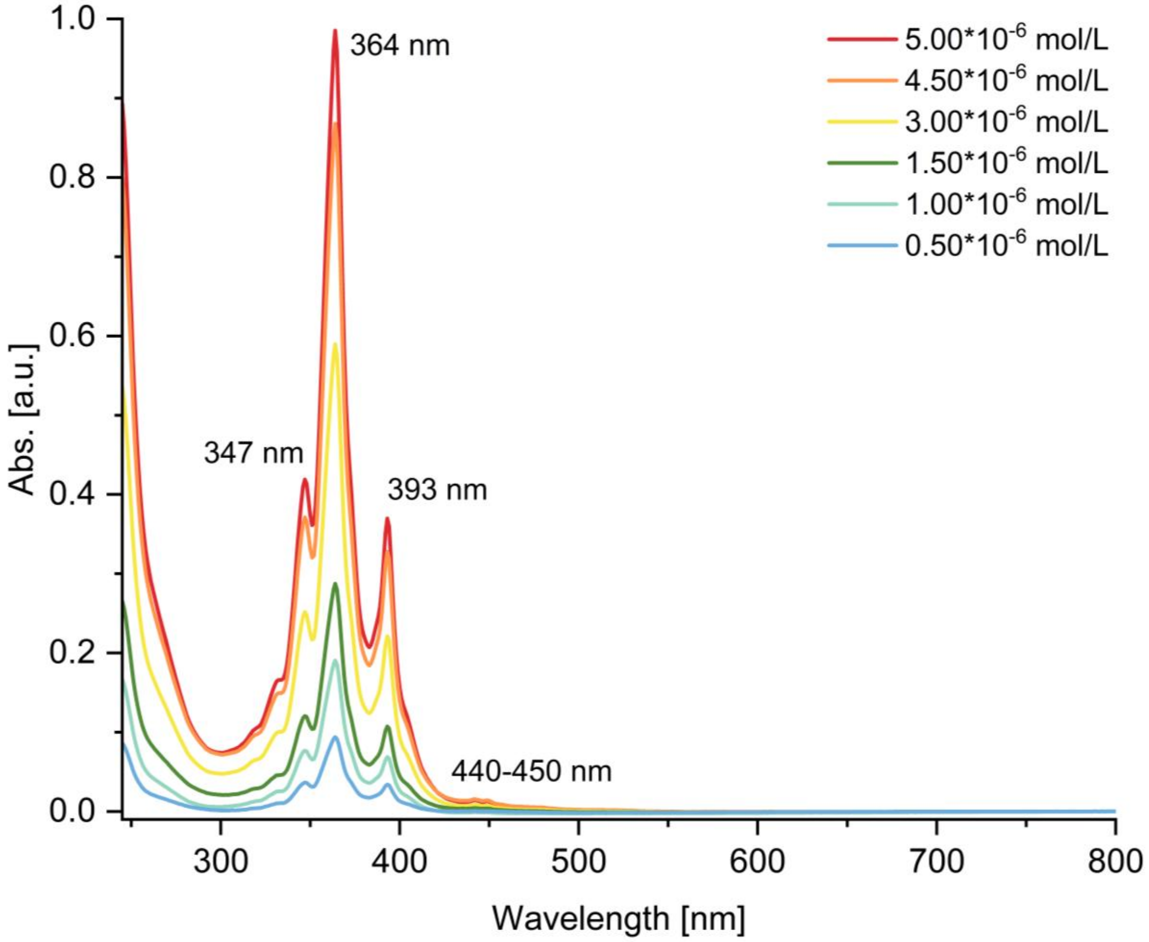

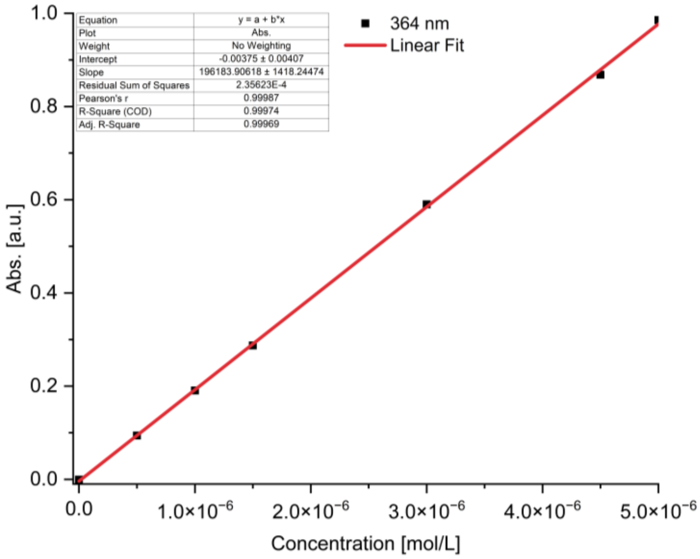

*Figure S43: Steady state absorption spectra of **HBC-Ref** in DCM: dilution row (top) and resulting linear fit at the global maximum, 364 nm.*

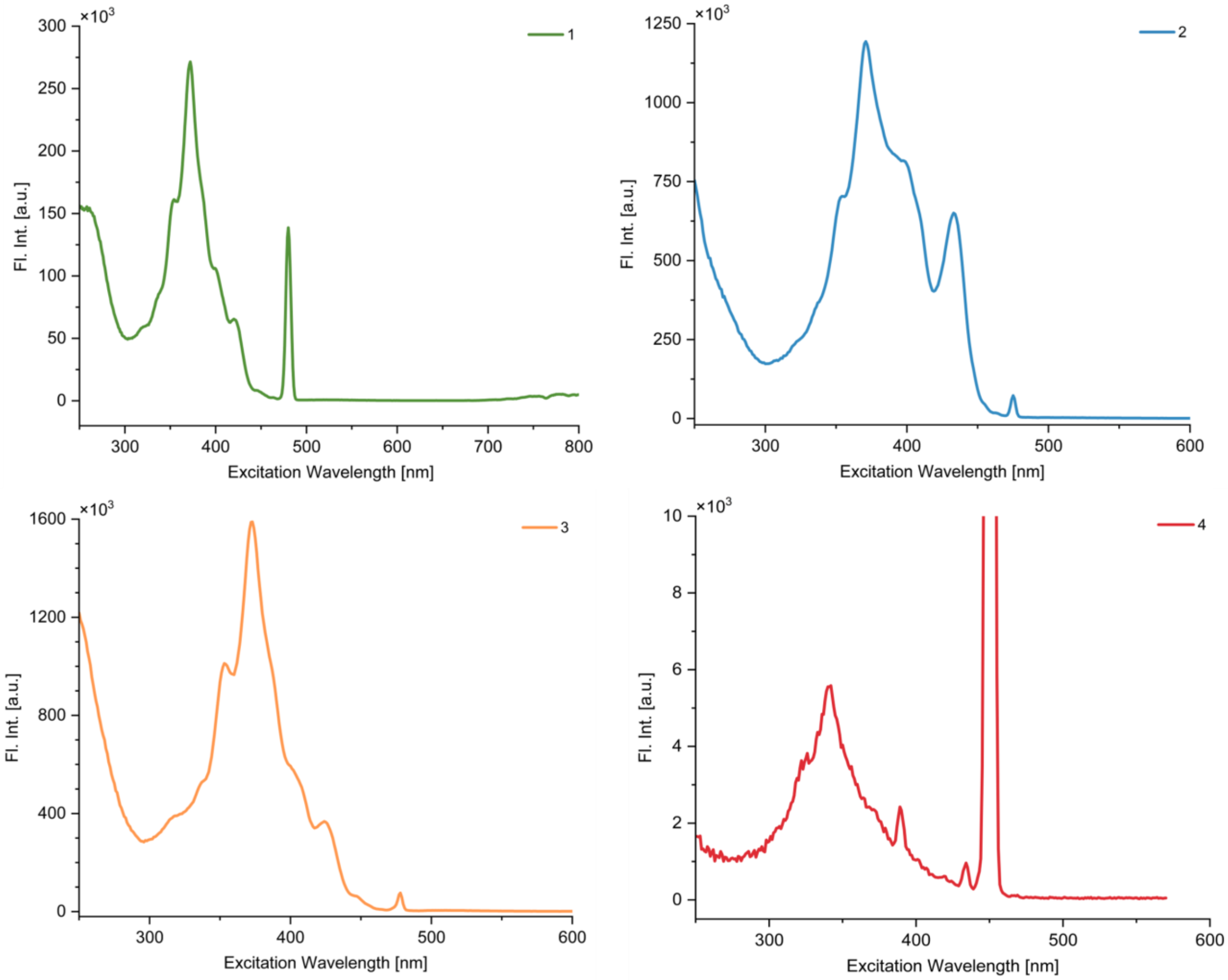

*Figure S44: Excitation spectra of HBCs **1-4** in DCM and c = 1×10^{-6} mol/L.*

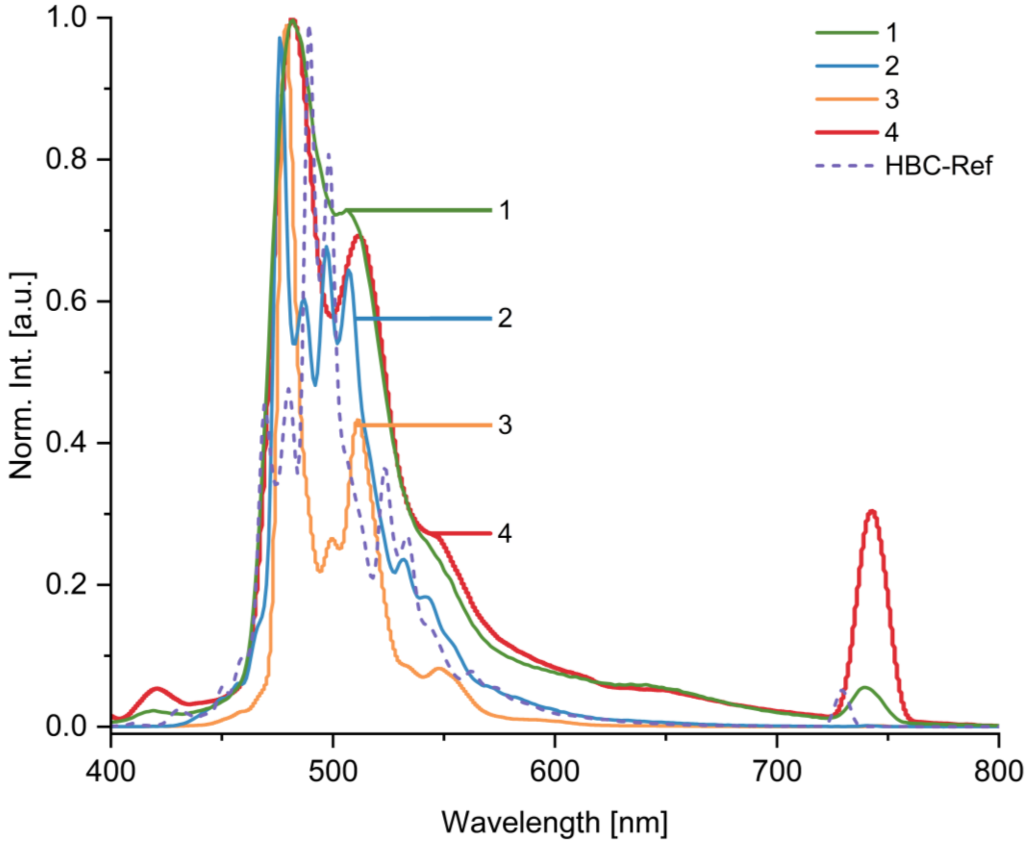

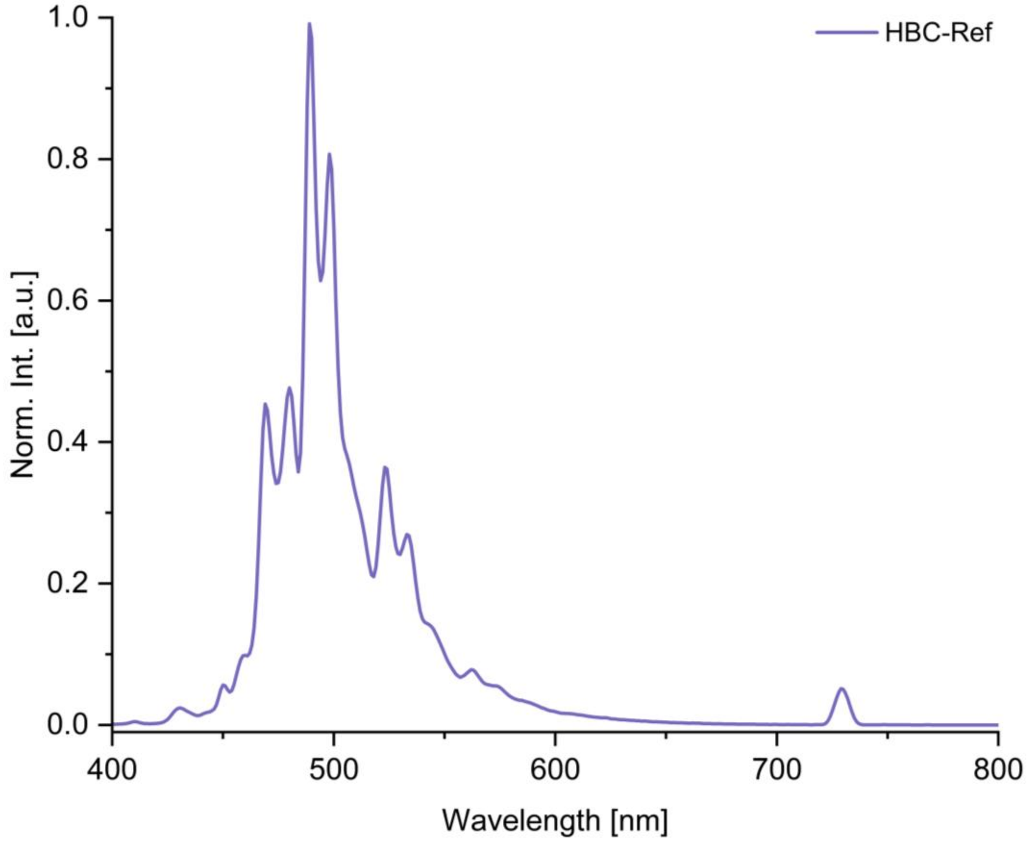

*Figure S45: Normalized steady state emission spectra of compounds **1-4** and **HBC-Ref** in DCM (top) and only **HBC-Ref** (bottom).*

Theoretical Investigation

Computational Methods

The molecules were built in the gas phase using Maestro 2024.[62] Next, geometry optimizations were performed within DFT calculations using the DEF2-SVP basis set and the BP86 density functional in combination with the with D4 dispersion correction like it is implemented in the Orca version 5.0.4.[63] The analysis of the excited states molecular orbitals was done with TDDFT at the CAM-B3LYP-D4 level using the DEF2-SVP basis set. For visualization of the charge density difference and prediction of the absorption spectrum we used Multiwfn, VMD and Python, respectively.[64,65]

Charge Density Difference Plots

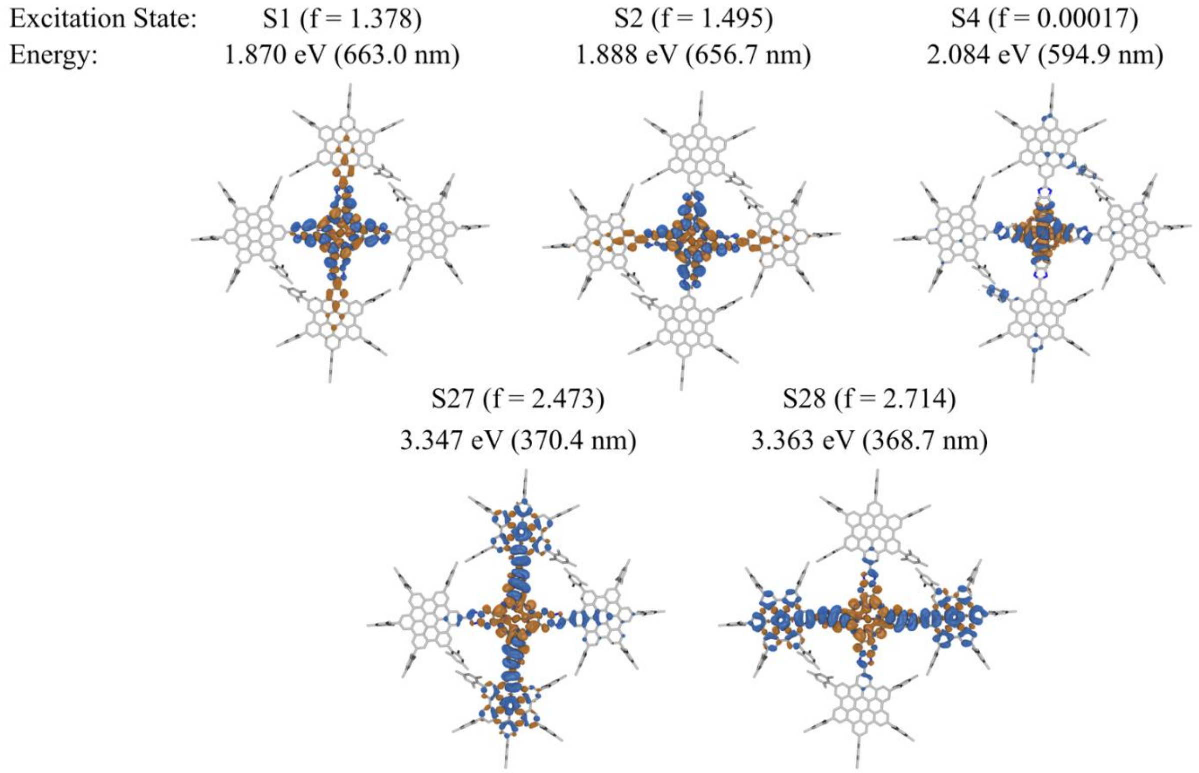

Figure S46: Charge density differences plots for selected excitation states of HBC-Pc ***1*** *(blue = negative, orange = positive, with iso values of ± 0.00001 for negative and positive isosurfaces respectively). Only nickel, carbon and nitrogen atoms are shown. The excitation states to S27 and S28 show strong oscillator strength in the high energy region and are mainly π-π* and inter-ligand charge transfers (ILCT). Excitation states to S1 and S2 have a little lower oscillator strength and are in low energy region. They show ligand to metal charge transfers (LMCT). Metal to ligand charge transfers (MLCT) are found in the energy region between 460 nm and 630 nm but with very low oscillator strengths of f < 0.0002. The energies to the states are taken after redshifting the calculated vacuum spectrum by 0.25 eV.*

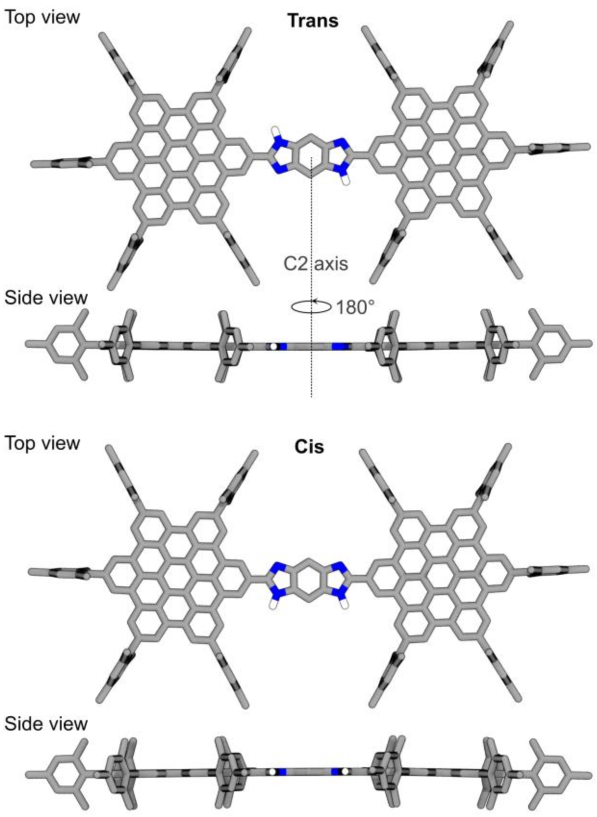

*Figure S47: Geometry of HBC **2** for the two tautomers, density functional theory calculated in vacuum (only carbon and nitrogen atoms are shown, for symmetrical reasons two hydrogen atoms are shown). The* trans- *structure shows a C2 symmetry through the central benzene (black dashed line). In the side view, one can see that the molecule is almost perfectly flat. The* cis- *structure is not as flat and lightly bent. The mesityl substituents are rotated only slightly for both tautomers. According to Boltzmann distribution on the calculated vacuum energies of the optimized geometries, the* trans-*tautomer is 7.484 times more likely than the* cis-*tautomer at 300 K.*

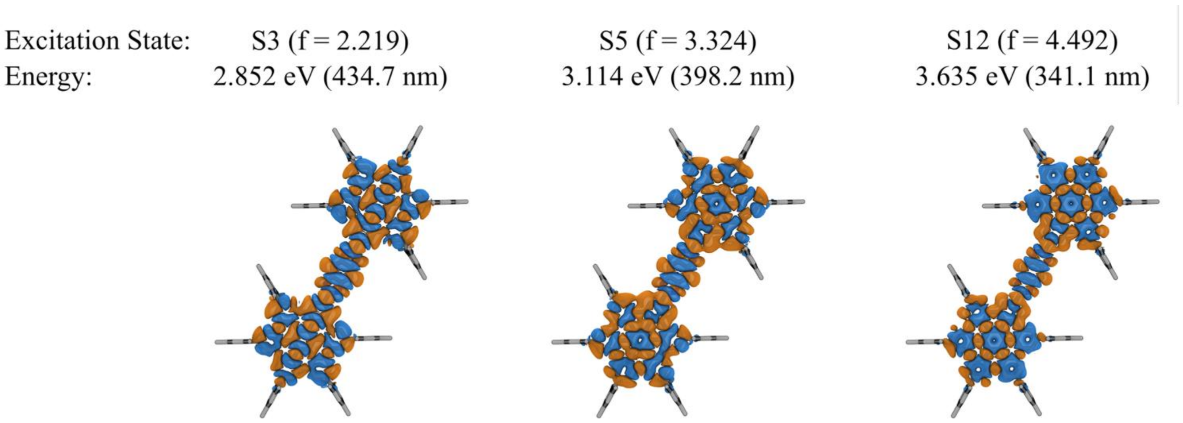

*Figure S48: Charge density differences plots for selected states (blue = negative, orange = positive, with iso values of ± 0.00001 for negative and positive isosurfaces respectively) of HBC **2**. Only carbon and nitrogen atoms are shown. The excitation to states S5 and S12 show very strong oscillator strength in the high energy region and are mainly π-π* and inter-ligand charge transfers (ILCT). Excitation state to S3 has a little lower oscillator strength but also strong π-π* and ILCT. The energies to the states are taken after redshifting the calculated vacuum spectrum by 0.422 eV.*

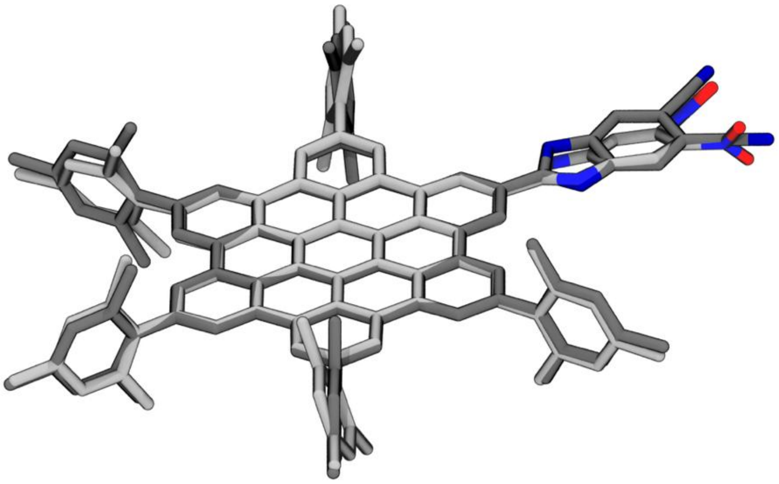

*Figure S49: Comparison of the density functional theory optimized geometries of HBC **3** (darkened) and HBC **4** in vacuum. Only carbon, nitrogen and oxygen atoms are shown. The HBC moieties of both molecules are largely congruent. The mesityl groups are partly rotated. In comparison to HBC **4**, the benzimidazole group of HBC **3** is somewhat lifted and tilted. The RMSD of the HBC moieties is 1.27 Å.*

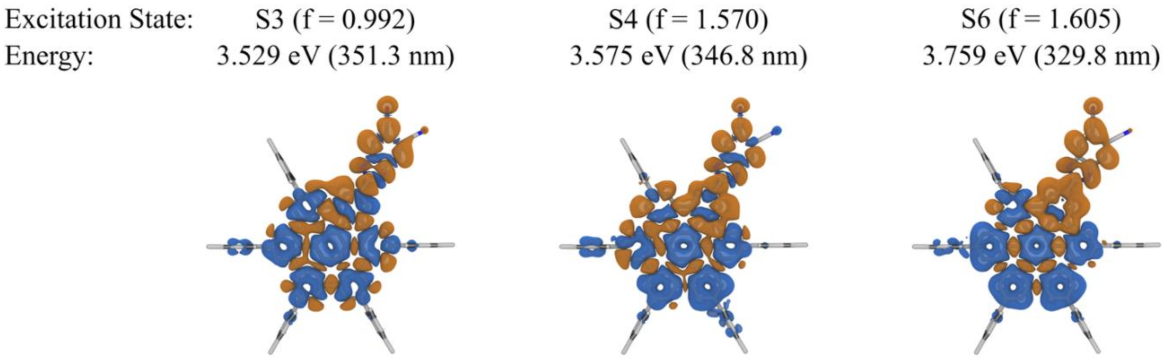

*Figure S50: Charge density differences plots for selected states (blue = negative, orange = positive, with iso values of ± 0.00001 for negative and positive isosurfaces respectively) of HBC **3**. Only carbon and nitrogen atoms are shown. The excitation states to S4 and S6 show strong oscillator strength in the high energy region and are mainly π-π* and inter-ligand charge transfers (ILCT). Excitation state to S3 has a little lower oscillator strength but also shows π-π* and ILCTs. The energies to the states are taken after redshifting the calculated vacuum spectrum by 0.45 eV.*

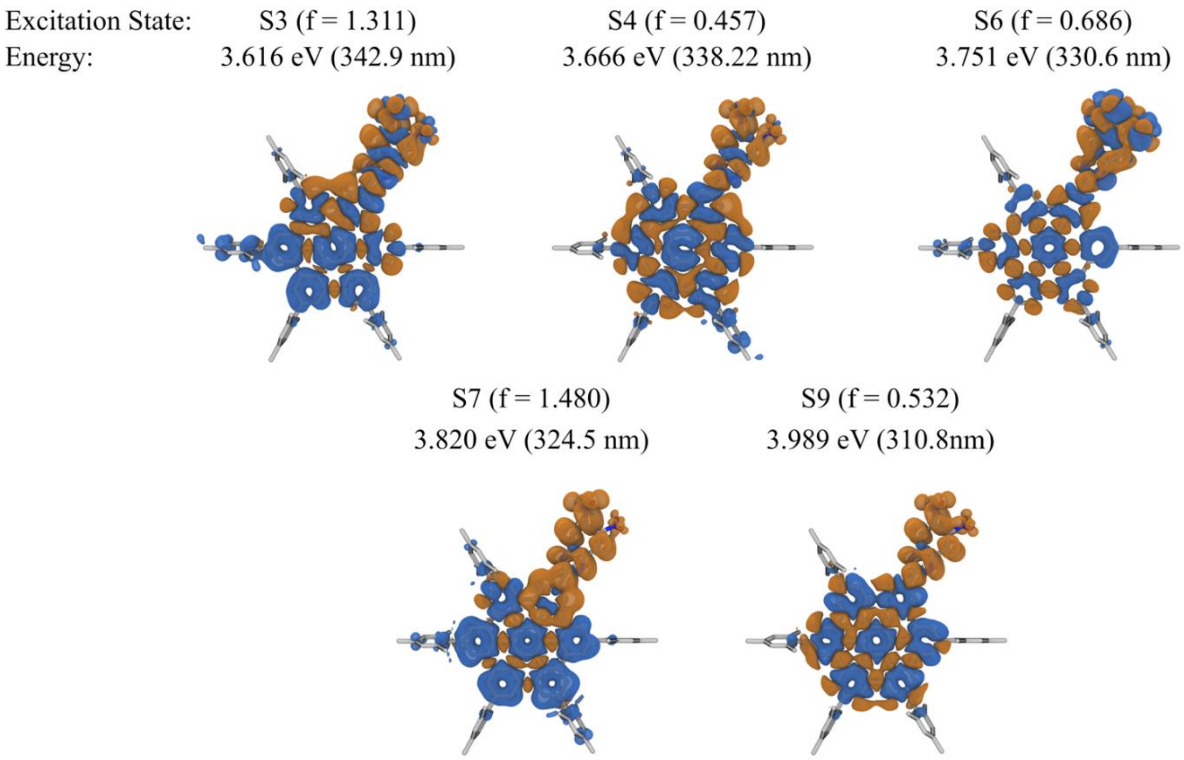

*Figure S51: Charge density differences plots for selected states (blue = negative, orange = positive, with iso values of ± 0.00001 for negative and positive isosurfaces respectively) of HBC **4**. Only carbon, nitrogen and oxygen atoms are shown. The excitation states to S3 and S7 show strong oscillator strength in the high energy region and are mainly π-π* and inter-ligand charge transfers (ILCT). Excitation states to S4 and 9 have a little lower oscillator strength but also strong π-π*. Excitation to S6 shows high ILCT on the NO_2-substituents and less strong π-π*. The energies to the states are taken after redshifting the calculated vacuum spectrum by 0.55 eV.*

TD-DFT Calculations

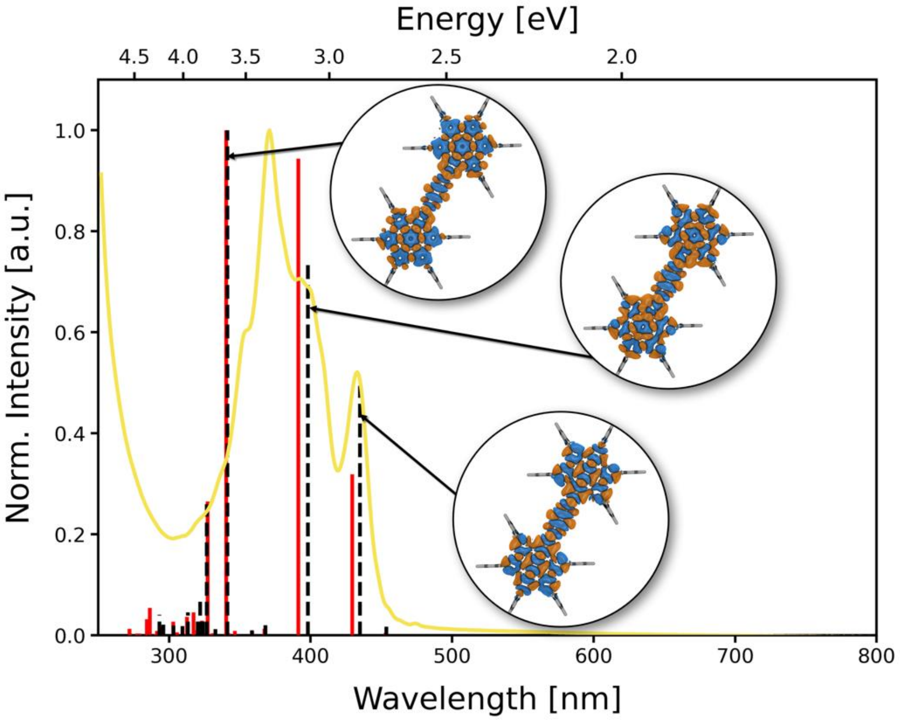

*Figure S52: Absorption spectrum for HBC **2** (yellow) compared to the calculated spectrum (black =* trans-*tautomer, red =* cis-*tautomer, see Figure S42 for structures of the tautomers) with charge density differences plots for selected transitions (negative = blue, positive = orange; only carbon and nitrogen atoms are shown). The calculation shows reasonably good matching in the lower energy region from ~390-440 nm with the measured absorption spectrum (redshifted by 0.422 eV). The signals with highest intensity are π-π* and inter-ligand charge transfers (ILCT). For illustration, a series of charge difference plots are shown for selected excitations for the trans tautomer with iso values of ± 0.00001 for negative and positive isosurfaces respectively. The* trans-*tautomer has been chosen for illustration because after Boltzmann the* trans-*tautomer is 7.484 times more likely than the* cis-*tautomer at 300 K. The signal with the highest intensity at 341.1 nm is ~0.3 eV blue shifted for tautomers in comparison to the main experimental peak at 371 nm. As the* trans-*tautomer is much more likely than the* cis- *and both geometries, especially the* trans-*tautomer, are very flat in comparison to all other compounds, it could be possible that HBC **2** is dimerizing and therefore responsible for the difference in the spectra.*

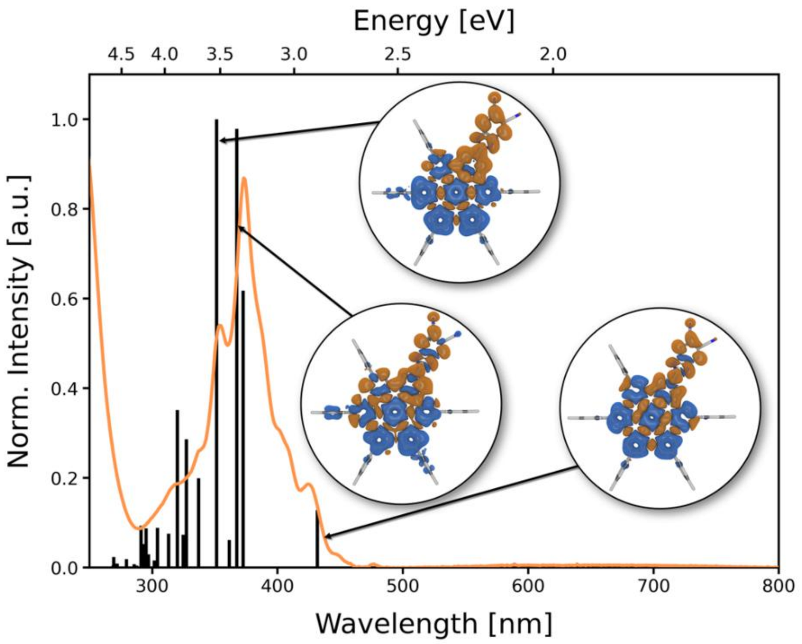

*Figure S53: Absorption spectrum of HBC **3** (orange) compared to the calculated spectrum (black) with charge density differences plots for selected transitions (negative = blue, positive = orange; only carbon and nitrogen atoms are shown). The calculation shows reasonably good matching with the measured absorption spectrum (redshifted by 0.45 eV). The signals with highest intensity are π-π* and inter-ligand charge transfers (ILCT). For illustration, a series of charge difference plots are shown for selected excitations with iso values of ± 0.00001 for negative and positive isosurfaces respectively.*

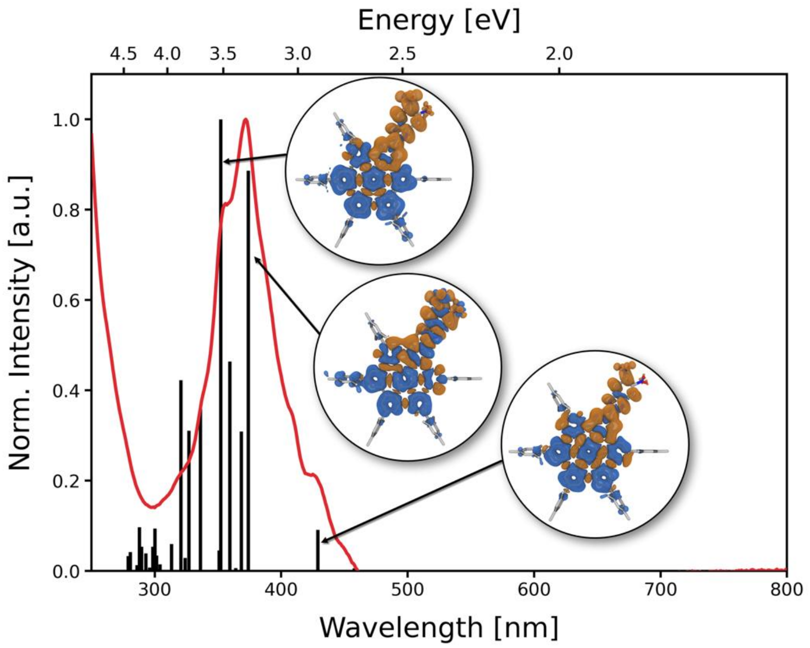

*Figure S54: Absorption spectrum of HBC **4** (red) compared to the calculated spectrum (black) with charge density differences plots for selected transitions (negative = blue, positive = orange; only carbon, nitrogen and oxygen atoms are shown). The calculation shows reasonably good matching with the measured absorption spectrum (redshifted by 0.55 eV). The signals with highest intensity are π-π* and inter-ligand charge transfers (ILCT). For illustration, a series of charge difference plots are shown for selected excitations with iso values of ± 0.00001 for negative and positive isosurfaces respectively*

Faraday Rotation Measurements

Faraday Rotation Measurements

Faraday rotation measurements of HBC-Pc **1** were carried out using a home-built spectrometer which simultaneously records magneto-optical rotation and absorption across the 450-1000 nm spectral range.[66] The magnetic field was swept in a sequence from 0 T to +0.5 T, then to -0.5 T, and finally back to 0 T. Solution-phase measurements were performed in toluene using a 1 mm quartz cuvette. Thin films were prepared by drop-casting a chloroform solution of HBC-Pc **1** onto high-precision #1.5H glass coverslips (170 ± 5 µm, Thorlabs CG15KH). After solvent evaporation, the films were annealed in chloroform vapor inside a sealed glass chamber and subsequently dried on a hot plate at 80 °C. Film thickness (150 ± 20 nm) was measured by profilometry using a Dektak 6M surface profiler.

Thin Films for Faraday Rotation measurements

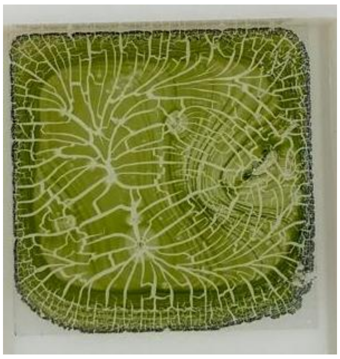

*Figure S55: Thin film of HBC-Pc **1** after drop casting from chloroform.*

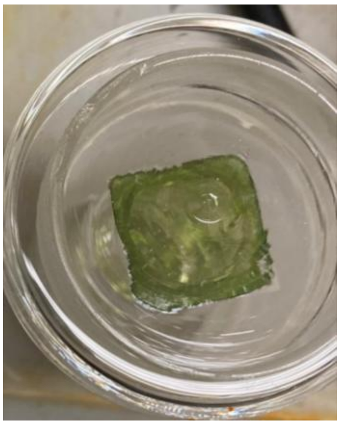

*Figure S56: Solvent annealing the HBC-Pc **1** film with chloroform vapors in a glass chamber.*

Photographs: Solutions in Ambient Light and UV-Irradiation

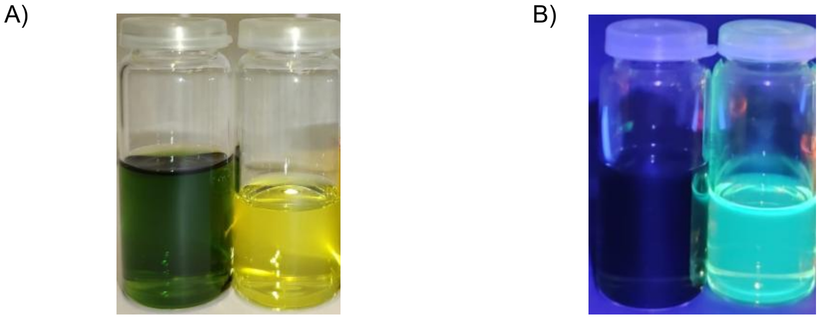

*Figure S57: A) HBC-Pc **1** (left vial) and HBC **2** (right vial) in ambient lighting; B) HBC-Pc **1** (left vial) and HBC **2** (right vial) under irradiation with 360 nm. All in DCM solution.*

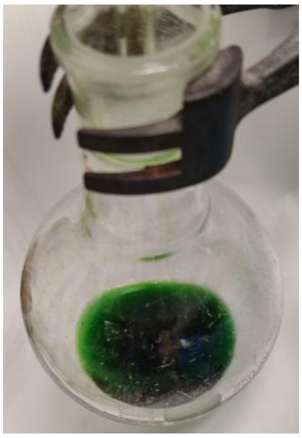

*Figure S58: Concentrated solution of HBC-Pc **1** in chloroform.*

References

[7] J. Buck, F. Hampel, A. Hirsch, "Triskelion-Shaped Hexabenzocoronenes: Synthesis and Characterization of Tris-Substituted HBC Derivatives" *Chem. Eur. J.* **2025**, *31*, e202404000.

[48] H. Jia, G. Zhuang, Q. Huang, J. Wang, Y. Wu, S. Cui, S. Yang, P. Du, "Synthesis of Giant π-Extended Molecular Macrocyclic Rings as Finite Models of Carbon Nanotubes Displaying Enriched Size-Dependent Physical Properties" *Chem. Eur. J.* **2020**, *26*, 2159.

[57] C. M. Cardona, W. Li, A. E. Kaifer, D. Stockdale, G. C. Bazan, "Electrochemical considerations for determining absolute frontier orbital energy levels of conjugated polymers for solar cell applications" *Adv. Mater.* **2011**, *23*, 2367.

[58] S. Trasatti, "The absolute electrode potential: an explanatory note (Recommendations 1986)" *Pure Appl. Chem.* **1986**, *58*, 955.

[59] Sauriat-Dorizon, H.; Maris, T.; Wuest, J. D.; Enright, G. D. "Molecular tectonics. Construction of porous hydrogen-bonded networks from bisketals of pentaerythritol" *J. Org. Chem.* **2003**, *68*, 240.

[60] R. Kurata, K. Kaneda, A. Ito, "Luminescent Superbenzene with Diarylamino and Diarylboryl Groups" *Org. Lett.* **2017**, *19*, 392.

[61] Y. Nakagawa, R. Sekiguchi, J. Kawakami, S. Ito, "Preparation of a large-sized highly flexible carbon nanohoop" *Org. Biomol. Chem.* **2019**, *17*, 6843.

[62] Schrödinger Release 2024: Maestro, Schrödinger LLC, New York, NY (United States of America), **2024**.

[63] F. Neese, "Software update: The ORCA program system—Version 5.0" *Wiley Interdiscip. Rev. Comput. Mol. Sci.* **2022***,* 12:e1606.

[64] W. Humphrey, A. Dalke, K. Schulten, "VMD: visual molecular dynamics" *J. Mol. Graph.* **1996**, *14*, 33.

[65] T. Lu, F. Chen, "Multiwfn: a multifunctional wavefunction analyzer" *J. Comput. Chem.* **2012**, *33*, 580.

[66] P. Wang, I. Jeon, Z. Lin, M. D. Peeks, S. Savagatrup, S. E. Kooi, T. van Voorhis, T. M. Swager, "Insights into Magneto-Optics of Helical Conjugated Polymers" *J. Am. Chem. Soc.* **2018**, *140*, 6501.
